# Long-Term Effect of Weight Regain Following Behavioral Weight Management Programs on Cardiometabolic Disease Incidence and Risk: Systematic Review and Meta-Analysis

**DOI:** 10.1161/CIRCOUTCOMES.122.009348

**Published:** 2023-03-28

**Authors:** Jamie Hartmann-Boyce, Annika Theodoulou, Jason L. Oke, Ailsa R. Butler, Anastasios Bastounis, Anna Dunnigan, Rimu Byadya, Linda J. Cobiac, Peter Scarborough, F.D. Richard Hobbs, Falko F. Sniehotta, Susan A. Jebb, Paul Aveyard

**Affiliations:** 1 Nuffield Department of Primary Care Health Sciences (J.H.-B., A.T., A.R.B., A.B., R.B., F.D.R.H., S.A.J., P.A.), University of Oxford, United Kingdom.; 2 Nuffield Department of Population Health, Centre on Population Approaches for Non-Communicable Disease Prevention (L.J.C.), University of Oxford, United Kingdom.; 3 Nuffield Department of Population Health, Oxford Biomedical Research Centre (P.S.), University of Oxford, United Kingdom.; 4 NIHR Oxford Biomedical Research Centre, Oxford University Hospitals NHS Foundation Trust, United Kingdom (J.L.O.).; 5 Division of Epidemiology and Public Health, School of Medicine, University of Nottingham, United Kingdom (A.B.).; 6 Oxford University Hospitals NHS Foundation Trust, United Kingdom (A.D.).; 7 Royal Free London NHS Foundation Trust, United Kingdom (A.D.).; 8 United Nations World Food Programme, Cox’s Bazar, Bangladesh, India (R.B.).; 9 Faculty of Medical Sciences, Population Health Sciences Institute, Newcastle University, United Kingdom (F.F.S.).

**Keywords:** adult, cardiovascular diseases, glycated hemoglobin, meta-analysis, obesity, systematic review, weight loss

## Abstract

Behavioral weight management programs (BWMPs) enhance weight loss in the short term, but longer term cardiometabolic effects are uncertain as weight is commonly regained. We assessed the impact of weight regain after BWMPs on cardiovascular risk factors, diabetes, and cardiovascular disease.

**Methods:**

Trial registries, 11 databases, and forward-citation searching (latest search, December 19) were used to identify articles published in English, from any geographical region. Randomized trials of BWMPs in adults with overweight/obesity reporting cardiometabolic outcomes at ≥12 months at and after program end were included. Differences between more intensive interventions and comparator groups were synthesized using mixed-effects, meta-regression, and time-to-event models to assess the impact of weight regain on cardiovascular disease incidence and risk.

**Results:**

One hundred twenty-four trials reporting on ≥1 cardiometabolic outcomes with a median follow-up of 28 (range, 11–360) months after program end were included. Median baseline participant body mass index was 33 kg/m^2^; median age was 51 years. Eight and 15 study arms (7889 and 4202 participants, respectively) examined the incidence of cardiovascular disease and type 2 diabetes, respectively, with imprecise evidence of a lower incidence for at least 5 years. Weight regain in BWMPs relative to comparators reduced these differences. One and 5 years after program end, total cholesterol/HDL (high-density lipoprotein) ratio was 1.5 points lower at both times (82 studies; 19 003 participants), systolic blood pressure was 1.5 mm mercury and 0.4 mm lower (84 studies; 30 836 participants), and HbA1c (%) 0.38 lower at both times (94 studies; 28 083 participants). Of the included studies, 22% were judged at high risk of bias; removing these did not meaningfully change results.

**Conclusions:**

Despite weight regain, BWMPs reduce cardiometabolic risk factors with effects lasting at least 5 years after program end and dwindling with weight regain. Evidence that they reduce the incidence of cardiovascular disease or diabetes is less certain. Few studies followed participants for ≥5 years.

**Registration:**

URL: https://www.crd.york.ac.uk/PROSPERO/; Unique identifier: CRD42018105744.

**What is Known**

- Behavioral weight management programs enhance weight loss in the short term, but longer term effects on cardiometabolic disease incidence and risk of weight loss interventions after treatment stops are uncertain as weight is commonly regained.

**What the Study Adds**

- We systematically reviewed 124 trials reporting change in cardiovascular risk factor, diabetes, or cardiovascular disease that followed participants after the end of the behavioral weight management program. The median follow-up was 28 (range, 11–360) months after program end.
- There was clear evidence that, compared with lower intensity behavioral weight management programs or control groups, intervention lowered cardiovascular risk factors at program end, and this improvement was apparent for at least 5 years, albeit diminishing with greater weight regain in the behavioral weight management program than comparator groups.
- The evidence suggested that the same was true for cardiovascular disease and diabetes but was too sparse to make high-certainty conclusions.

**See Editorial by Pagidipati et al**

Obesity is a major risk factor for premature morbidity and mortality worldwide, primarily driven by cardiovascular disease (CVD).^1^ There are linear associations between adiposity and adverse lipid profile, blood pressure, and insulin resistance that largely explain the higher risk of CVD in people with excess adiposity.^2^ Offering treatment for overweight and obesity is recommended in guidelines to prevent CVD.^3^ There is good evidence that weight loss during treatment programs lowers blood pressure and glycemia and improves lipid profile.^4–6^ However, weight loss is commonly followed by weight regain, and some observational studies suggest this weight change pattern may increase cardiovascular risk,^7^ but data from randomized trials are lacking.

Individual trials are commonly powered to measure effects on weight loss and individually lack power to assess the impact on cardiometabolic risk factors and disease incidence. Here, we draw on a large systematic review of trials that examined weight change after program end of behavioral weight management programs (BWMPs) to conduct a meta-analysis of the legacy effects on cardiovascular risk factors and on the incidence of CVD. We did not aim to estimate the impact of particular interventions but of interventions that led to weight loss, which, once they cease, are likely to be followed by weight regain. Two hundred and forty-nine trials could be included in the meta-analysis of weight regain. Together, more intensive interventions led to −2.8 kg (95% CI, −3.2 to −2.4) greater weight loss at program end, and thereafter, weight regain occurred at 0.12 to 0.32 kg/year more than comparator, with the estimate depending on model choice.^8^ With few and particular exceptions, there was little evidence that program characteristics altered the rate of weight regain.^9^ Here, we assessed whether weight regain after the programs finished was associated with change in cardiometabolic risk and incident disease. The aim was not to assess the effectiveness of any particular intervention but to assess the effects of interventions that aim to enhance weight loss which, once withdrawn, are typically followed by weight regain.

## Methods

The detailed methods are provided in the preregistered published protocol.^10^ The review had several outcomes, and this report examines cardiometabolic risk factors and incident cardiovascular and cardiovascular-related disease. The extracted data are available to others on reasonable request. Ethical review by an institutional review board was not sought as this is secondary research.

### Search

We searched for randomized controlled trials of BWMPs versus any comparator in clinical trial registries and 11 electronic databases in September 2018 using terms relating to obesity, weight loss, diet, exercise, behavior change, and terms relevant to BWMPs. We searched a specialized register of weight loss trials hosted by the University of Aberdeen. Searches were run since inception but restricted to full articles published in English. We contacted the authors for supplementary information. Before analysis (December 2019), we ran a forward-citation search for follow-up studies of included trials.

### Eligibility Criteria

We included randomized controlled trials of BWMPs for adults (≥18 years) with overweight or obesity at the study start (body mass index of ≥25 or ≥23 kg/m^2^ in Asian populations). Comparators had to be another BWMP, an intervention of lesser intensity, or no intervention, thus allowing us to compare interventions achieving greater weight loss against those achieving less. Trials of multiple risk factor interventions and interventions or control groups that also included medication or surgery were excluded. Our focus was on long-term outcomes after BWMPs, so studies had to follow participants for ≥12 months from baseline and measure weight change at program end and afterward. Program end was not always clearly defined, so we defined it as the point at which the intervention intensity markedly stepped down (eg, when contact became less frequent than once every 2 months or when a step change in frequency or author-defined shift from weight loss to weight maintenance begins; see the protocol for more detail).^10^ We confined this analysis to studies reporting cardiometabolic outcomes, namely incidence of cardiovascular morbidity/mortality (including both primary and secondary prevention), incidence/remission of type 2 diabetes and hypertension, and changes in systolic blood pressure (SBP), serum cholesterol, blood glucose, and insulin measures. We extracted data on weight change after program end to assess its association with cardiometabolic indicators.

### Screening, Data Extraction, and Risk-of-Bias Assessments

Two reviewers independently screened studies for inclusion against the eligibility criteria using Covidence review management software.^11^ The team developed a bespoke database for data extraction, which was piloted and agreed. Data extraction and risk-of-bias assessment were conducted by one reviewer and checked by a second reviewer. Risk of bias was assessed for random sequence generation, allocation concealment, blinding of outcome assessment, attrition, and other risk of bias.^12^ Any discrepancies throughout the screening, extraction, and critical appraisal processes were resolved by discussion, sometimes involving the whole team.

### Data Synthesis

We calculated change in outcomes from baseline for outcomes at program end and at each time point after program end for all arms. Standardized mean differences (SMDs) were used where variables measured the same construct (eg, plasma glucose and HbA1c as measurements of glycemic control) to enhance power; they were then back converted to a common unit for illustrative purposes. For cholesterol, these were combined such that higher values represented higher cardiovascular risk. Where multiple measures were available, we preferred total cholesterol/HDL (high-density lipoprotein) ratio, followed by total cholesterol. We extracted results reported by authors; in nearly all included studies, this meant that we used complete case or multiple imputation data. For dichotomous outcomes (disease incidence and remission), we used the definitions used by study authors.

We calculated pooled weighted averages at program end to put the results in context, but our focus was on events and risk factors beyond program end. For each arm, we calculated the difference in incidence or mean risk factor between BWMP and its comparator at each time point after program end. Thus, negative values indicate that people in BWMPs have a lower incidence or lower cardiometabolic risk, zero represents no difference, and positive values that the incidence or risk factor is higher in people randomized to BWMP than comparator. We compared BWMPs to their comparator, providing the comparator was either no intervention, a minimal intervention, or a lower intensity BWMP. Our aim here was to examine the impact of weight loss and subsequent regain on cardiometabolic outcomes, not to estimate the effect of particular programs on these outcomes.

We analyzed these data using 3 methods to assess whether the results were sensitive to choice of synthesis method. The 3 methods were as follows:

Mixed model with a random intercept for each study, regressing the difference in mean outcome between intervention and comparator at every time reported in follow-up after program end. This was the primary analysis incorporating all data points nested within arms but was unweighted by study precision.^13^
Meta-regression against time since program end, assuming linear increases in outcomes plotted as baseline (program end) value and outcome at the longest follow-up only. This weighted studies by their variance (precision).^14^
Kaplan-Meier plot of time to event, with failure represented by return of the intervention value to that of the comparator group.

We fitted models allowing for a curvilinear effects with time, but these did not improve fit, and we removed these terms for parsimony. Models 1 and 2, therefore, yielded linear slope coefficients. Given that there was usually a difference in favor of BWMPs at program end, negative values imply that the difference incidence or mean value between BWMP grew larger with time, zero represented a constant difference, and positive values that the difference between the BWMP and comparator declined with time. We graphed these slopes to ease interpretation.

We also used meta-regression to examine whether a decrease in weight difference between BWMP compared with control, that is, faster regain in BWMP arms than in control arms, was associated with incidence of, or risk factors for, cardiometabolic disease.

Preregistered sensitivity analyses excluded studies at high risk of bias in any domain. All analyses were performed in R 4.0.2.^15^

## Results

### Search Results

Our initial searches retrieved 17 085 references, 4482 of which progressed to full-text screening. The most common reason for exclusion at full-text stage was follow-up duration of <12 months (Figure S1). An additional 246 relevant references were identified through forward-citation searching and the screening of trial websites of large studies. Eight hundred and seventy-nine references representing 330 studies met our inclusion criteria. Authors of 53 included studies provided additional data or information. One hundred and twenty-four studies provided data on changes in cardiometabolic disease incidence or risk factors and were included here.^16–138^

### Characteristics of Included Studies

Table 1 shows summary data for included studies. The median body mass index of participants at baseline was 33 kg/m^2^, and median age was 51 years. Detail on individual studies can be found in Table S1 (primary references), Table S2 (risk of bias assessments summary), Table S3 (risk of bias assessments), Table S4 (key characteristics), Table S5 (baseline demographics), and Table S6 (intervention characteristics). Programs typically lasted 7 months, and length of follow-up throughout refers to time since program end. Studies had on average 28 months follow-up after program end (range, 11–360 months).

**Table 1.**
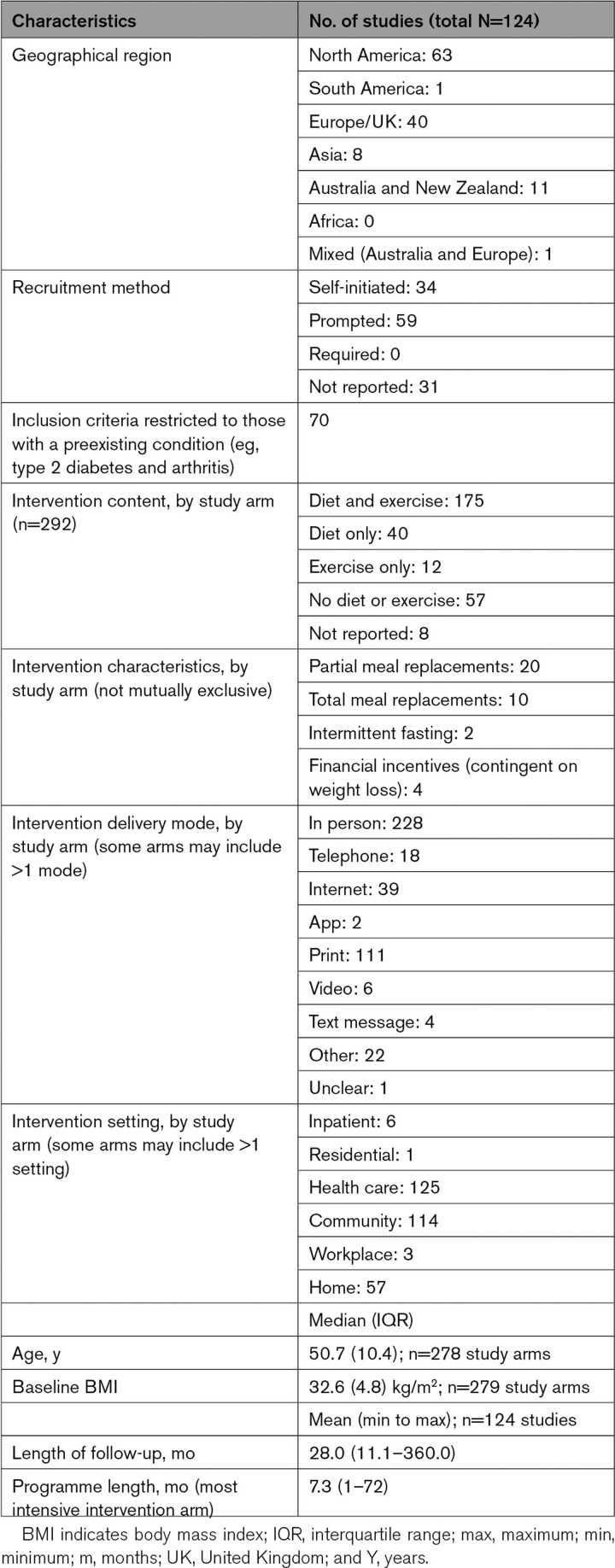
Summary Information on Characteristics of Studies Contributing to Statistical Analyses

### Risk of Bias

Fifty-two percent of studies were at unclear risk of bias, primarily because they did not fully report randomization procedures, 27% at low risk and 22% at high risk (Table S2). Judgements for each study with reasons are in Table S3.

### Effects of Interventions

#### Incidence of CVD

Eight studies (7889 participants) had data on cardiovascular morbidity or mortality at, or after, program end (longest follow-up, 288 months). The mean weight difference at program end was −2.2 (SD, 1.8) kg (Table 2). There was no evidence that weight gain in BWMP relative to that in the comparator was associated with changing incidence of CVD. The estimated difference in incidence for 1 kg regain in intervention relative to the comparator group was −10.3 (−41.9 to 21.4)/1000 person-months.

**Table 2.**
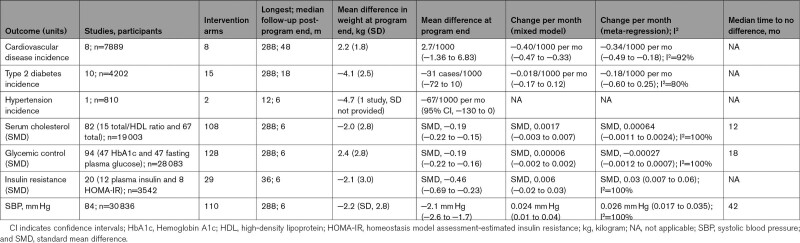
Summary Outcome Data (Estimates Presented With 95% CIs)

At program end, there was also no evidence that the observed incidence of CVD was higher in intervention than comparator at 2.7/1000 person-months (−1.36 to 6.83; Table 2), but only 2 studies reported data at this time point. However, the fitted incidence from the random effects model (accounting for data from all studies at all time points) favored intervention over comparator with a difference in incidence of −15.4/1000 person-months at program end.

**Figure 1.**
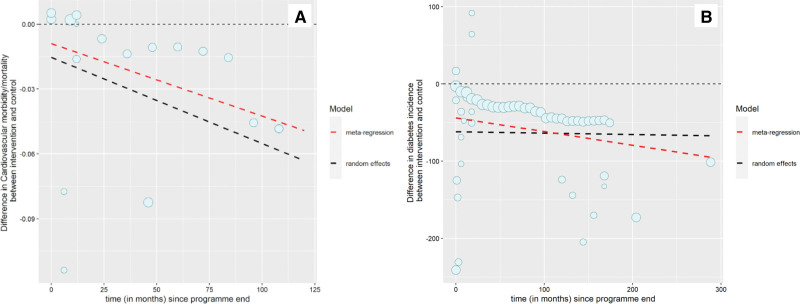
**Difference in disease incidence. A**, Cardiovascular disease incidence (cases per 1000 per month) between intervention and comparator arms by time since program end. **B**, Type 2 diabetes incidence (cases per 1000 per month) between intervention and comparator arms by time since program end. Dot size is proportional to the number of participants in each study. Lines represent estimates of average trend from random effects and meta-regression.

After program end, the incidence was estimated to decline relative to the comparator group by −0.40/1000 person-months (−0.47 to −0.33; Figure 1A; Table 2). This means that the predicted CVD incidence 1 year after BWMP program end would be −20.2/1000 person-months and at 5 years −39.3/1000 person-months lower than in comparator groups. The results using meta-regression were similar, giving a slope coefficient predicting a decline in incidence of CVD with time at −0.34/1000 person-months (−0.49 to −0.19; Figure 1A; Table 2). Only 4 studies remained after removing studies at high risk of bias, so sensitivity analyses were not conducted.

#### Incidence and Remission of Type 2 Diabetes

Fifteen intervention arms from 10 studies (4202 participants) reported incidence of type 2 diabetes (hereafter, diabetes; longest follow-up, 288 months post-program end). The mean difference in weight between intervention and comparator at program end was −4.1 (SD, 2.5) kg (Table 2). There was no evidence that weight change was associated with diabetes incidence difference (estimated change per kilogram of weight difference, 0.0022/1000 person-months [95% CI, −0.0072 to 0.012]).

There was also no evidence that the observed mean incidence of diabetes at program end in people randomized to BWMP was lower than comparator (5 studies), with a mean difference in incidence (95% CI) of −31 cases per 1000 people (−72 to 10; Table 2). The modeled incidence (accounting for all other data points) was lower at −62/1000 person-months at program end and was estimated to stay approximately constant, with a slope coefficient of −0.018/1000 person-months (−0.17 to 0.12) in the random effects model (Figure 1B). This gave a predicted lower incidence of type 2 diabetes 1 year after program end of −62/1000 person-months and 5 years after of −63/1000 person-months in people randomized to BWMP than to comparator groups. The meta-regression predicted a slightly greater advantage over time for BWMP than random effects (Figure 1B). Sensitivity analyses removing studies at high risk of bias left 7 studies with maximum follow-up of 18 months. These analyses gave estimates of trend that implied that the incidence of diabetes would return toward that in the comparator group. The slope coefficients from the random effects and meta-regression models were 0.7/1000 person-months (−4.5 to 9.3) and 5.2/1000 person-months (−2.8 to 13.0; Table 2).

Two studies reported diabetes remission at program end.^64,130^ One study reported a nonsignificant difference in remission rates of 5/1000 person-months (95% CI, −11/1000 to 25/1000 person-months) and one reported a significantly higher rate of remission in the intervention arm (risk difference, −5/1000 [95% CI, −9 to −0/1000] person-months). No studies reported remission after program end, and hence, we could not analyze the association of weight regain with diabetes remission.

#### Incidence and Remission of Hypertension

Two intervention arms from a single study provided follow-up data on hypertension incidence beyond program end compared with minimal control.^24^ Given there was only one study, we did not estimate the association between weight change after program end and difference in incidence of hypertension. Average difference in hypertension incidence was −67/1000 person-months (95% CI, −130 to 0) at program end and −28/1000 person-months (95% CI, −93 to 37) at 12 months after program end (Table 2).

Four studies reported hypertension remission data at program end (n=1266). There was no evidence of a difference in hypertension remission between arms at program end (pooled mean, −11 per 1000 [95% CI, −390 to 370]) or at the last follow-up time (estimated difference in hypertension remission, 230 per 1000 [95% CI, −330 to 780]).

#### Cholesterol

One hundred and eight intervention arms from 82 studies (n=19 003) were included. The longest follow-up was 288 months. The mean weight difference at program end was −2.0 kg (2.8), and observed SMD in lipid indices was −0.19 ([95% CI, −0.22 to −0.15] equating to a reduction in total cholesterol/HDL ratio [median (interquartile range)] of 1.2 [1.0–1.4]; Table 2). There was evidence that weight regain was associated with change in cholesterol. Each kilogram gained in the intervention group relative to the control group decreased the difference in favor of BWMP relative to control by 0.034 (0.022–0.047).

**Figure 2.**
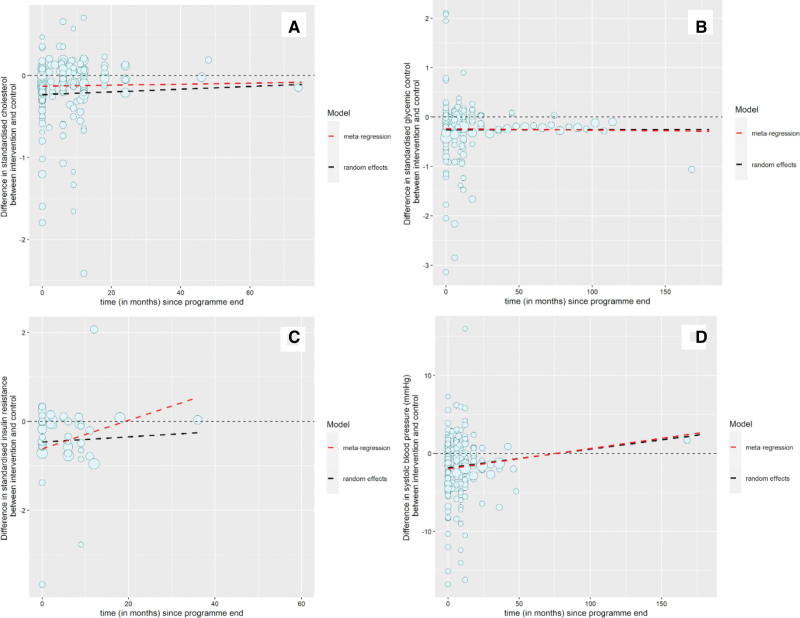
**Difference in cardiovascular disease risk factors. A**, Difference in standardized mean lipid change between intervention and comparator arms by time since program end. **B**, Difference in standardized mean glycemic control change between intervention and comparator arms by time since program end. **C**, Difference in standardized mean insulin resistance change between intervention and comparator arms by time since program end. **D**, Difference in systolic blood pressure change between intervention and comparator arms by time since program end. Dot size is proportional to the number of participants in each study. Lines represent estimates of average trend from random effects and meta-regression.

Forty studies had follow-up data beyond program end, and using these, the modeled SMD at program end was −0.23. After program end, there was no evidence that cholesterol in people randomized to BWMP returned to that of the comparator group, although the central estimate implied a convergence. The random effects coefficient was 0.0017 per month (−0.003 to 0.007; Figure 2A; Table 2). The meta-regression results gave a similar slope estimate (Figure 2A; Table 2). Thus, from the random effects model, the predicted SMD at 1 and 5 years after program end was the same, at −0.23 lower in BWMP than control groups, equivalent to 1.5 lower total cholesterol/HDL ratio. From Kaplan-Meier analysis, the median time for the difference in lipid indices to return to the comparator group was 12 months (Figure S2). Sensitivity analyses removing studies at high risk of bias did not meaningfully alter findings from any of the 3 models (Table S7). Thus, the random effects and meta-regression models favored at least a 5-year reduction in adverse lipid profile for people randomized to BWMP versus comparators, while Kaplan-Meier suggested around 1 year.

#### Glycemic Control

One hundred and twenty-eight intervention arms from 94 studies (n=28 083) reported data on HbA1c (47 studies) or fasting plasma glucose (47 studies), pooled as SMD. The longest follow-up was 288 months. The mean weight difference between BWMP and comparator at program end was −2.4 (SD, 2.8) kg. There was no evidence of an association between weight regain and SMD in glycemic control. For each kilogram of weight regain in BWMP compared with comparator, the estimated change in SMD of glycemic control was 0.00071 (−0.010 to 0.012).

The observed SMD in glycemic control at program end was −0.19 (−0.22 to −0.16), equivalent to a median (interquartile range) difference in HbA1c (%) of 0.18 to 0.37 (Table 2). Using random effects modeling incorporating all data, the modeled SMD at program end was −0.26, equivalent to an HbA1c (%) of 0.25 to 0.51. There was no evidence that the improved glycemic control in BWMPs changed with time, with a slope coefficient of 0.000057 (−0.0021 to 0.0022; Figure 2B; Table 2). The meta-regression coefficient was similar: 0.00027 (−0.0012 to 0.0007; Figure 2B; Table 2). Thus, the modeled estimate was that glycemic control at 1 year would be −0.26 and at 5 years −0.26 lower in BWMPs than comparator. The Kaplan-Meier analysis suggested that the median time for glycemic control to return to control was 18 months (Figure S3). Sensitivity analyses removing studies at high risk of bias did not meaningfully change the estimates, with no evidence that the benefit of BWMP on glycemic control changed with time (Table S7). Thus, the random effects and meta-regression models favored at least a 5-year reduction in glycemic control for people randomized to BWMP versus comparators, whereas Kaplan-Meier suggested around 1.5 years.

#### Insulin Resistance

Twenty-nine intervention arms from 20 studies including 3542 participants reported data on insulin (plasma insulin or HOMA-IR). The longest follow-up was 36 months, and the mean difference in weight change at program end was −2.1 (3.0) kg (Table 2). There was evidence that weight regain was associated with change in insulin resistance. For every kilogram participants in BWMPs gained relative to the control group, insulin resistance decreased by −0.062 (95% CI, −0.11 to −0.016). Removing studies at high risk of bias meant this counterintuitive finding, which was no longer statistically significant (0.054 [95% CI, −0.022 to 0.13]).

The observed average (95%) difference between BWMP and control in SMD of insulin resistance was −0.46 (−0.69 to −0.23) at program end (Table 2). Modeling from the random effects model, the estimate at program end was −0.46. Thereafter, there was no evidence that the slope changed with time, with a random effects coefficient of 0.006 per month (−0.02 to 0.03; Figure 2C; Table 2). This would predict that at 1 year after program end, insulin resistance would be −0.46 and at 3 years −0.45 lower after BWMPs than the comparator. The meta-regression estimate was somewhat different, predicting a return of BWMP to comparator by 20 months, with a slope coefficient of 0.032 per month (0.007–0.057; Figure 2C; Table 2). In Kaplan-Meier analysis, the median time to return to no difference from control could not be estimated as fewer than half of the studies reached this. Thus, the random effects model favored at least a 3-year reduction in insulin resistance for people randomized to BWMP versus comparators, whereas meta-regression ≈2 years and Kaplan-Meier at least 3 years.

#### Systolic Blood Pressure

One hundred and ten intervention arms from 84 studies with 30 836 participants reported data on SBP with the longest follow-up of 288 months. The mean difference in weight between BWMP and comparator at program end was −2.2 (SD, 2.8) kg (Table 2). The observed mean (95% CI) SBP at program end was −2.1 (−2.6 to −1.7) mm Hg lower in BWMPs than comparators, very similar to the modeled estimate from random effects modeling of −1.8 mm (−2.6 to −1.1; Table 2). There was strong evidence that weight regain was associated with a reduction in the advantage of BWMP over comparator. For every kilogram regained in the BWMP relative to comparator, the blood pressure difference between BWMP and comparator reduced by 0.45 (95% CI, 0.36–0.54) mm Hg. Removing studies at high risk of bias did not significantly alter estimates (Table S7).

Random effects modeling suggested that SBP would converge on the comparator after program end at 0.024 mm Hg per month (0.011–0.037), with nearly identical estimates from meta-regression (Figure 2D; Table 2). The modeled SBP difference between BWMP and comparator at 1 year was −1.5 mm Hg and at 5 years −0.4 mm Hg. In Kaplan-Meier analysis, the median time to return to no difference from comparator was estimated at 42 months (Figure S4). Thus, the random effects and meta-regression models favored a 6-year reduction in SBP for people randomized to BWMP versus comparators, whereas Kaplan-Meier suggested around 3.5 years.

## Discussion

This is the largest ever synthesis of extant evidence of the long-term impact of weight regain following BWMPs on cardiometabolic disease and risk factors. We found relatively few studies that examined the incidence of CVD or diabetes beyond program end but those that had suggested that the incidence was lower while the observation continued (up to 24 years). Too few studies examined incidence and remission of hypertension or remission from diabetes to draw reliable conclusions. Far more data were available on risk factors for cardiometabolic disease, measured by glycemic control, cholesterol, and blood pressure. Each risk factor was lower at program end following BWMP than for the comparator, and this advantage persisted through follow-up, typically for at least 3 and commonly at least 5 years, though these estimates varied by risk factor and by analysis method. For all but glycemic control, there was evidence that over time, weight regain following BWMPs relative to the comparator groups reduced the cardiometabolic risk factor reductions seen in BWMP relative to control groups.

This large review has limitations, partly as a result of it aiming to be a comprehensive overview of the long-term cardiometabolic effects of regain following the end of BWMPs. First, the large amount of handsearching meant our search took place in 2019, and the process of data extraction, contacting authors, and analysis meant we conducted a limited update, identifying new publications of subsequent data from the studies already included in an attempt to increase the duration of follow-up, where data were particularly scant. Studies meeting our inclusion criteria first published after December 2019 were excluded. Likewise, we excluded studies in languages other than English. These decisions themselves should not bias the outcomes under investigation but mean a few studies are likely to have been missed.

Our results also include substantial heterogeneity (as demonstrated, in part, by high I^2^ values, which were for the most part driven by magnitude rather than direction of effect). In aiming for a comprehensive synthesis, we a priori planned methods to pool different measures of the same construct, such as HbA1c and fasting plasma glucose. These measures will move in the same direction, but not necessarily to the same extent, and so pooling may have introduced some heterogeneity, but pooling improved precision and clarity of the answer. We also pooled studies that compared a BWMP to no intervention, a minimal intervention, or a more substantial but lower intensity BWMP. We did so because our aim was to assess the long-term, post-program effect of BWMP-induced weight loss followed by weight regain on cardiometabolic risk and, in particular, what happens long term after weight loss has ceased and weight is regained. This was not designed to test the effectiveness of particular BWMPs, and the results might be broadly applicable to any weight loss intervention including pharmacotherapy where the intervention is pursued for some months then withdrawn. The estimates of weight loss provided here or in our companion review should not, therefore, be taken as estimates of treatment effect of BWMPs. The aim was to assess whether, and how quickly, the established short-term weight loss and cardiometabolic benefits are eroded by weight regain. This heterogeneity of comparison would affect point estimates at a particular point, but there is little evidence or reason to believe that heterogeneity of interventions or comparisons affect weight the trajectory of regain and thereby affecting change in cardiometabolic disease or risk factors after program end.^9^

The bulk of evidence we have assembled relates to cardiometabolic risk factors, with only a little evidence on disease outcomes themselves, where evidence was sparse and conclusions more uncertain. The key to interpreting these data is evidence that changing cardiometabolic risk factors will eventually translate to differences in disease incidence and mortality. For instance, the Food and Drug Administration mandates that new diabetes medications are now tested in cardiovascular outcomes trials following evidence that rosiglitazone led to adverse cardiovascular outcomes despite reducing blood glucose.^139^ These effects, however, came about through adverse effects on increasing cardiovascular volume and adverse effects on LDL (low-density lipoprotein) cholesterol, rather than directly caused by glucose lowering, which might otherwise have been expected to reduce the incidence of CVD.^140^ BWMPs lower all cardiovascular risk factors and, in the short-term, have been shown to reduce all-cause mortality,^141^ so this should allay some concerns that the evidence mostly relates to proxy outcomes, where there is incontrovertible evidence that lowering blood pressure and improving lipid profile reduce cardiovascular risk.^142–144^

Evidence we present in a companion review suggests that weight loss following a BWMP leads to at least a 5-year reduction in population mean weight from BWMPs compared with no or minimal weight loss intervention.^8^ Data on risk factors presented here suggest a similar trajectory for cardiometabolic risk factors, in line with data that weight regain reduces the difference in cardiometabolic risk.^4–6^ Thus, BWMPs appear to lead to a temporary reduction in exposure to cardiometabolic risk factors that may last several, perhaps 5, years. Evidence suggests that these temporary reductions in risk factors are likely to lead to lifetime benefits of reduced incidence of CVD. For example, large, well-known studies (included in our review) have found that even though weight is regained following BWMPs, reductions in diabetes incidence persist at 13 to 15 years.^145,146^ There is also clear evidence from WOSCOPS (West of Scotland Coronary Prevention Study) that lowering LDL concentration for only 5 years resulted in a 20-year reduction in CVD (and thereby all-cause mortality), though the reductions observed in WOSCOPS are greater in magnitude than those observed in our analyses.^147^ The evidence that a temporary period of blood pressure reduction reduces CVD is less clear, but evidence from animal models and estimates from trials suggest similar legacy effects from even temporary reductions in blood pressure.^148–150^

These observed reductions in cardiovascular risk factors were observed from BWMPs relative to comparators (sometimes active treatments) of around 2 to 3 kg. Other analyses of this data set showed that some programs give much larger end-of-program weight losses compared with no weight loss support; for example, programs providing meal replacement.^9^ While larger initial weight loss is associated with faster weight regain,^9^ the initial advantage in weight loss was modeled to last at least 5 years. Taken together, data suggest that achieving larger initial weight losses is likely to reduce cardiometabolic risk to a greater extent—a benefit that may well last 5 years.

In summary, temporary interventions to achieve weight loss, such as BWMPs, lower cardiometabolic risk factors and may reduce the incidence of CVD and diabetes. This reduction in risk of exposure to adverse lipids, higher blood pressure, and dysglycemia lasts for several years after a BWMP compared with a lower intensity comparator but gradually erodes as weight is regained. This evidence reinforces the value of such programs to reduce the risk of CVD. It should reassure clinicians and patients that support for weight management will reduce their risk of premature morbidity and weight regain is unlikely to erode the lifetime benefits.

## Article Information

### Acknowledgments

The authors would like to thank the people living with overweight and obesity who shaped this work at the outset and Anna Whiting and Philippa Seeber for serving as patient and public involvement advisors on this work. Nia Roberts, subject librarian, designed and executed searches. Alison Avenell, Brian Taylor, and colleagues at Aberdeen provided invaluable support and advice throughout. Initial work on database construction was funded by the National Institute for Health Research HTA grant number 15/09/04, Review of Behaviour and Lifestyle Interventions for Severe Obesity: an Evidence Synthesis (REBALANCE). The authors thank Sarah King, Mary Logan, and Yolanda Warren for their help with study screening; Alex and Louis Robinson for their help with data entry; and Helen Parretti, Stephan Dombrowski, and Nerys Astbury for sharing data extraction forms from previous reviews. The authors are very grateful to the many authors who answered their queries and provided additional data for their analyses. J. Hartmann-Boyce, P. Aveyard, S.A. Jebb, F.F. Sniehotta, F.D.R. Hobbs, P. Scarborough, and J.L. Oke conceived and designed the review. J. Hartmann-Boyce, R. Byadya, A. Theodoulou, A.R. Butler, A. Bastounis, and A. Dunnigan conducted screening. J. Hartmann-Boyce, A. Theodoulou, A.R. Butler, A. Bastounis, and A. Dunnigan conducted data extraction and assessed studies for risk of bias. J.L. Oke conducted the main statistical analyses. L.J. Cobiac and P. Scarborough designed and conducted cost-effectiveness analyses. J. Hartmann-Boyce prepared the first draft of the review, with further input from A. Theodoulou, P. Aveyard, and S.A. Jebb. All authors contributed to the interpretation and final write-up.

### Sources of Funding

This research was funded by the British Heart Foundation, PG/17/68/33247, and the National Institute for Health Research (NIHR) Oxford Biomedical Research Centre (BRC) Obesity, Diet and Lifestyle theme. J. Hartmann-Boyce, P. Aveyard, S.A. Jebb, and F.D.R. Hobbs are partly funded by NIHR Oxford BRC. J. Hartmann-Boyce is also partly funded by an NIHR Cochrane Programme Grant. P. Aveyard and S.A. Jebb are also funded by NIHR Oxford Applied Research Centre. P. Aveyard is an NIHR senior investigator. F.D.R. Hobbs also acknowledges part-funding from the National Institute for Health Research School for Primary Care Research, the NIHR Applied Research Collaboration Oxford, and the NIHR Oxford MedTech and In-Vitro Diagnostics Co-Operative. P. Scarborough is funded by a BHF fellowship (FS/15/34/31656). The views expressed are those of the authors and not necessarily those of the British Heart Foundation, the National Health Service, the NIHR, or the Department of Health and Social Care. The study sponsors had no role in study design, collection, analysis, interpretation, writing, or the decision to submit the manuscript for publication.

### Disclosures

P. Aveyard and Jebb were investigators on a trial of a low-energy total diet replacement programme funded by the Cambridge Weight Plan. P. Aveyard spoke at a seminar at the Royal College of General Practitioners conference that was funded by Novo Nordisk. Neither of these led to personal payments. The other authors report no conflicts.

### Supplemental Material

Figures S1–S4

Tables S1–S7

## Supplementary Material
