# Supplementary material for: Long-Term Effect of Weight Regain Following Behavioral Weight Management Programs on Cardiometabolic Disease Incidence and Risk: Systematic Review and Meta-Analysis
Source: Circ Cardiovasc Qual Outcomes. 2023 Mar 28;16(4):e009348. doi: 10.1161/CIRCOUTCOMES.122.009348 (PMC10106109; doi:10.1161/CIRCOUTCOMES.122.009348)
Supplement: Supplementary file 1 [file hcq-16-e009348-s001.pdf]

SUPPLEMENTAL MATERIAL

Longer-term mental health outcomes following programmes: A systematic review with meta-analyses behavioural weight management

CONTENTS

Supplemental Figures and Figure Legend 2

Figure S1. PRISMA diagram of study flow 2

Figure S2. Kaplan Meier plot showing probability that study arm that had cholesterol difference at programme end is zero on follow-up. 3

Figure S3. Kaplan Meier plot showing probability that study arm that had glycaemic control difference at programme end is zero on follow-up....4

Figure S4. Kaplan Meier plot showing probability that study arm that had systolic blood pressure difference at programme end is zero on follow-up....5

Supplemental Tables....6

Table S1. Included studies reference list 6

Table S2. Risk of bias assessments summary....13

Table S3. Risk of bias of included studies....14

Table S4. Characteristics of included studies 57

Table S5. Baseline demographics....70

Table S6. Intervention characteristics....79

Table S7. Sensitivity analyses....122

Supplemental Figures and Figure Legend

Figure S1. PRISMA diagram of study flow

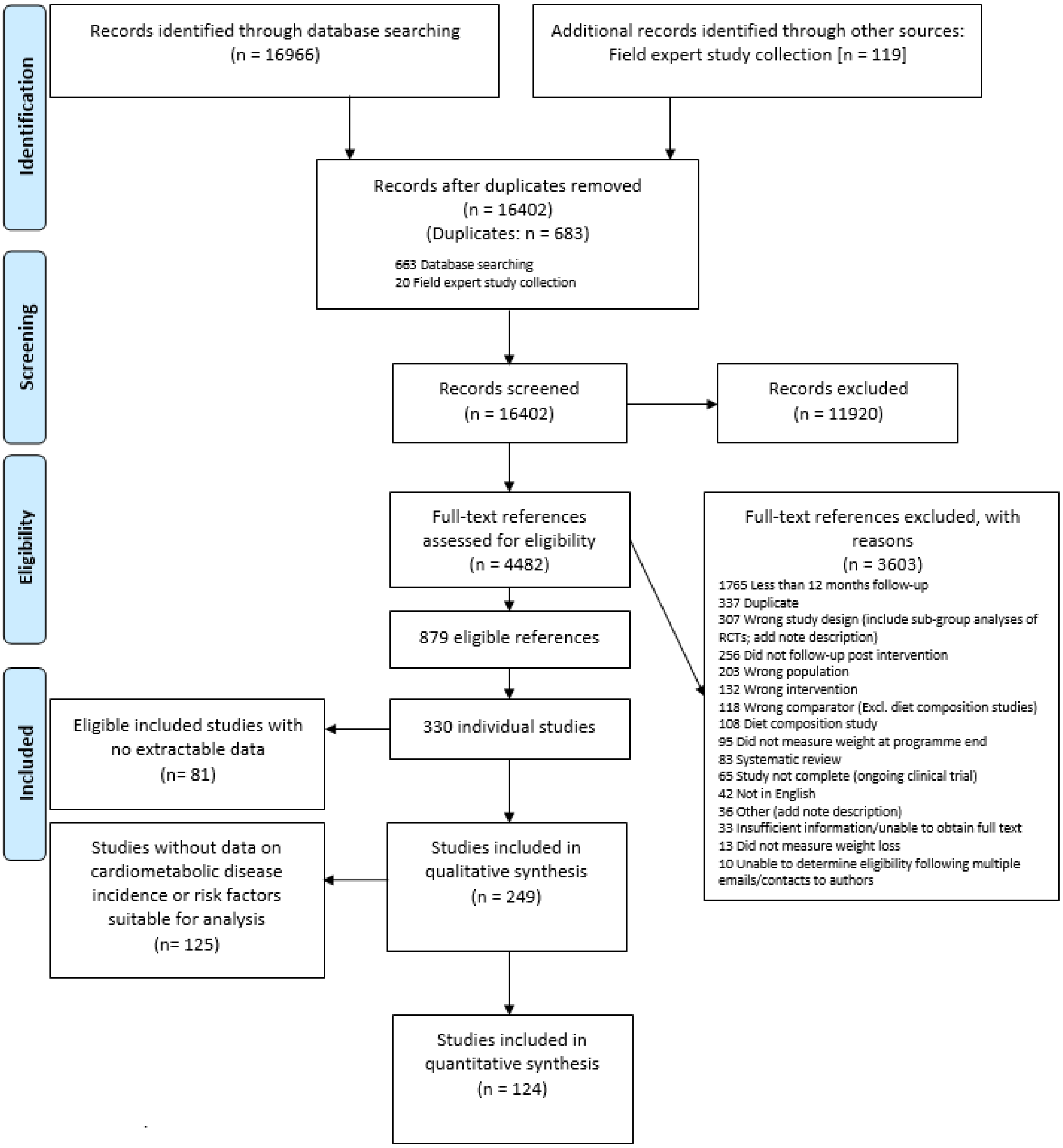

Figure S2. Kaplan Meier plot showing probability that study arm that had cholesterol difference at programme end is zero on follow-up.

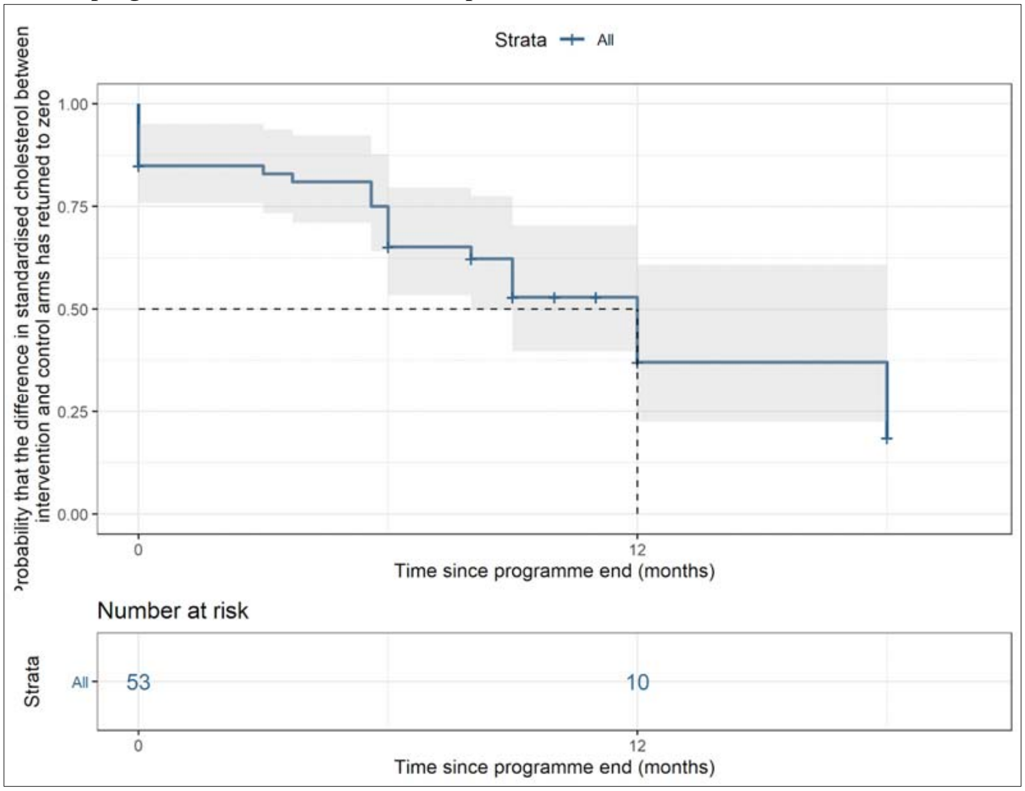

Figure S3. Kaplan Meier plot showing probability that study arm that had glycaemic control difference at programme end is zero on follow-up.

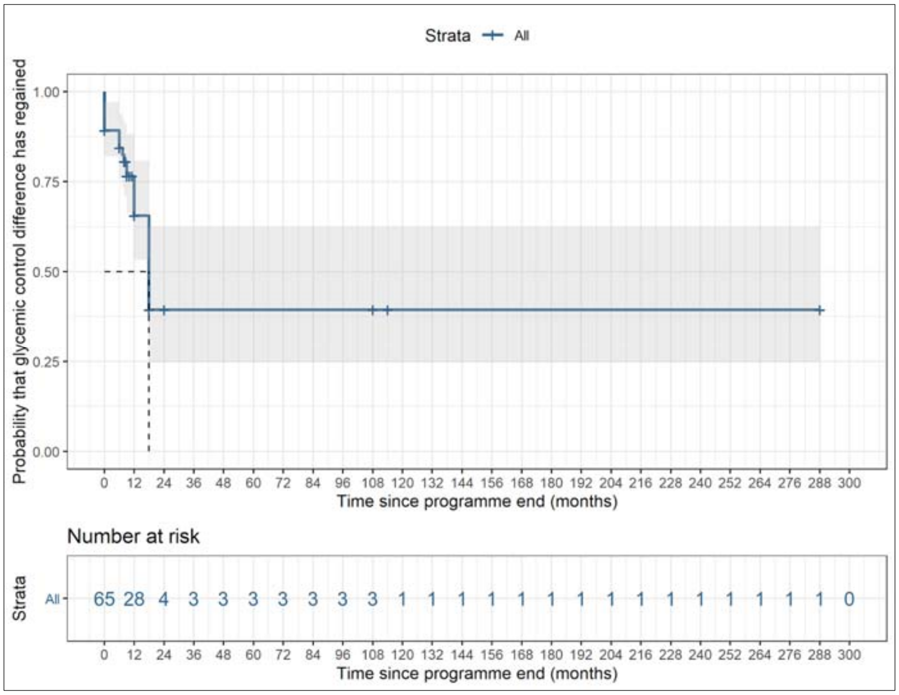

Figure S4. Kaplan Meier plot showing probability that study arm that had systolic blood pressure difference at programme end is zero on follow-up.

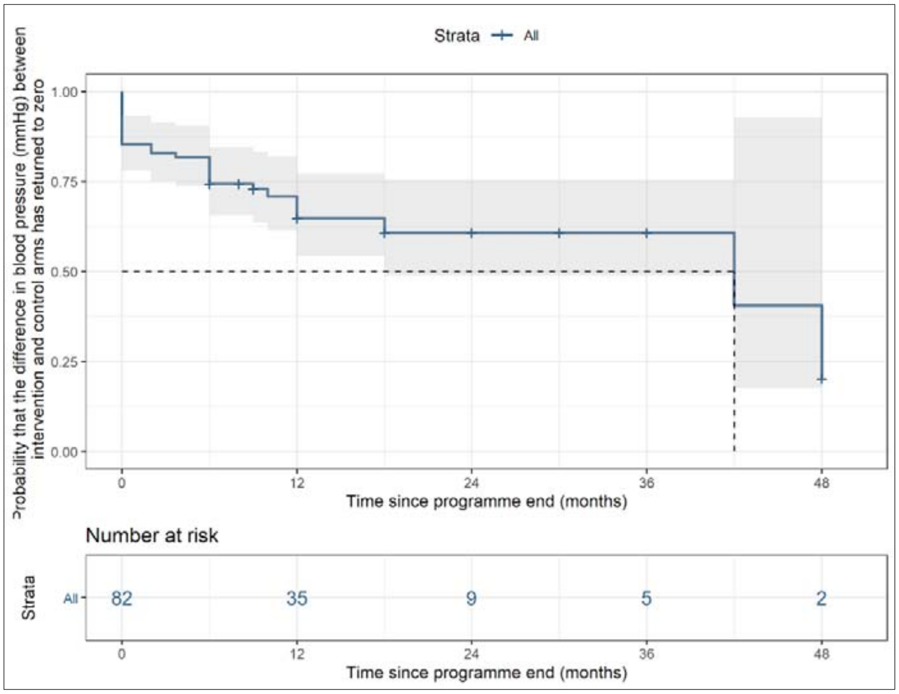

Supplemental Tables

Table S1. Included studies reference list

*Ref No. Reference number in main paper.

Study ID	Ref No.*	Primary reference
Abed 2013	16	Abed HS, Wittert GA, Leong DP, et al. Effect of weight reduction and cardiometabolic risk factor management on symptom burden and severity in patients with atrial fibrillation: a randomized clinical trial. *JAMA* 2013; **310**(19): 2050-60.
Ackermann 2011	17	Ackermann RT, Finch EA, Caffrey HM, Lipscomb ER, Hays LM, Saha C. Long-term effects of a community-based lifestyle intervention to prevent type 2 diabetes: the DEPLOY extension pilot study. *Chronic Illn* 2011; **7**(4): 279-90.
Ahern 2017	18	Ahern AL, Wheeler GM, Aveyard P, et al. Extended and standard duration weight-loss programme referrals for adults in primary care (WRAP): a randomised controlled trial. *Lancet* 2017; **389**(10085): 2214-25
Almanza -Aguilera 2018	19	Almanza-Aguilera E, Brunius C, Bernal-Lopez MR, et al. Impact in Plasma Metabolome as Effect of Lifestyle Intervention for Weight-Loss Reveals Metabolic Benefits in Metabolically Healthy Obese Women. *J Proteome Res* 2018; **17**(8): 2600-10.
Andersen 1999	20	Andersen RE, Wadden TA, Bartlett SJ, Zemel B, Verde TJ, Franckowiak SC. Effects of lifestyle activity vs structured aerobic exercise in obese women: a randomized trial. *JAMA* 1999; **281**(4): 335-40.
Anderson 2014	21	Anderson AS, Craigie AM, Caswell S, et al. The impact of a bodyweight and physical activity intervention (BeWEL) initiated through a national colorectal cancer screening programme: randomised controlled trial. *BMJ* 2014; **348**: g1823.
Appel 2011	22	Appel LJ, Clark JM, Yeh HC, et al. Comparative effectiveness of weight-loss interventions in clinical practice. *N Engl J Med* 2011; **365**(21): 1959-68.
Ard 2018	23	Ard JD, Gower B, Hunter G, et al. Effects of Calorie Restriction in Obese Older Adults: The CROSSROADS Randomized Controlled Trial. *J Gerontol A Biol Sci Med Sci* 2017; **73**(1): 73-80
Ard 2004	24	Ard JD, Grambow SC, Liu D, Slentz CA, Kraus WE, Svetkey LP. The effect of the PREMIER interventions on insulin sensitivity. *Diabetes Care* 2004; **27**(2): 340-7.
Ashley 2001	25	Ashley JM, St Jeor ST, Perumean-Chaney S, Schrage J, Bovee V. Meal replacements in weight intervention. *Obes Res* 2001; **9 Suppl 4**: 312s-20s.
Azar 2013	26	Azar KM, Xiao L, Ma J. Baseline obesity status modifies effectiveness of adapted diabetes prevention program lifestyle interventions for weight management in primary care. *Biomed Res Int* 2013; **2013**: 191209.
Bacon 2002	27	Bacon L, Keim NL, Van Loan MD, et al. Evaluating a 'non-diet' wellness intervention for improvement of metabolic fitness, psychological well-being and eating and activity behaviors. *Int J Obes Relat Metab Disord* 2002; **26**(6): 854-65.
Barnes 2017	28	arnes RD, Ivezaj V, Martino S, Pittman BP, Grilo CM. Back to Basics? No Weight Loss from Motivational Interviewing Compared to Nutrition Psychoeducation at One-Year Follow-Up. *Obesity (Silver Spring)* 2017; **25**(12): 2074-8.
Bartels 2015	29	Bartels SJ, Pratt SI, Aschbrenner KA, et al. Pragmatic replication trial of health promotion coaching for obesity in serious mental illness and maintenance of outcomes. *Am J Psychiatry* 2015; **172**(4): 344-52.
Beeken 2017	30	Beeken RJ, Leurent B, Vickerstaff V, et al. A brief intervention for weight control based on habit-formation theory delivered through primary care: results from a randomised controlled trial. *Int J Obes (Lond)* 2017; **41**(2): 246-54.
Bennett 2013	31	Bennett GG, Foley P, Levine E, et al. Behavioral treatment for weight gain prevention among black women in primary care practice: a randomized clinical trial. *JAMA Intern Med* 2013; **173**(19): 1770-7.
Bennett 2012	32	Bennett GG, Warner ET, Glasgow RE, et al. Obesity treatment for socioeconomically disadvantaged patients in primary care practice. *Arch Intern Med* 2012; **172**(7): 565-74.
Bertz 2012	33	Bertz F, Brekke HK, Ellegård L, Rasmussen KM, Wennergren M, Winkvist A. Diet and exercise weight-loss trial in

		lactating overweight and obese women. *Am J Clin Nutr 2012;* **96**(4): 698-705.
Bo 2007	34	Bo S, Ciccone G, Baldi C, et al. Effectiveness of a lifestyle intervention on metabolic syndrome. A randomized controlled trial. *J Gen Intern Med* 2007; **22**(12): 1695-703.
Burke 2005	35	Burke V, Beilin LJ, Cutt HE, Mansour J, Wilson A, Mori TA. Effects of a lifestyle programme on ambulatory blood pressure and drug dosage in treated hypertensive patients: a randomized controlled trial. *J Hypertens* 2005; **23**(6): 1241-9.
Chaiyasoot 2018	36	Chaiyasoot K, Sarasak R, Pheungruang B, et al. Evaluation of a 12-week lifestyle education intervention with or without partial meal replacement in Thai adults with obesity and metabolic syndrome: a randomised trial. *Nutr Diabetes* 2018; **8**(1): 23.
Chee 2017	37	Chee WSS, Gilcharan Singh HK, Hamdy O, et al. Structured lifestyle intervention based on a trans-cultural diabetes-specific nutrition algorithm (tDNA) in individuals with type 2 diabetes: a randomized controlled trial. *BMJ Open Diabetes Res Care* 2017; **5**(1): e000384.
Cheskin 2008	38	Cheskin LJ, Mitchell AM, Jhaveri AD, et al. Efficacy of meal replacements versus a standard food-based diet for weight loss in type 2 diabetes: a controlled clinical trial. *Diabetes Educ* 2008; **34**(1): 118-27.
Cheyette 2007	39	Cheyette C. Weight No More: a randomised controlled trial for people with type 2 diabetes on insulin therapy. *Pract Diab Int* 2007; **24**(9): 450-6.
Christensen 2012	40	Christensen JR, Overgaard K, Carneiro IG, Holtermann A, Søgaard K. Weight loss among female health care workers--a 1-year workplace based randomized controlled trial in the FINALE-health study. *BMC Public Health* 2012; **12**: 625.
Cole 2013	41	Cole RE, Boyer KM, Spanbauer SM, Sprague D, Bingham M. Effectiveness of prediabetes nutrition shared medical appointments: prevention of diabetes. *Diabetes Educ* 2013; **39**(3): 344-53.
Conroy 2015	42	Conroy MB, Sward KL, Spadaro KC, et al. Effectiveness of a physical activity and weight loss intervention for middle-aged women: healthy bodies, healthy hearts randomized trial. *J Gen Intern Med* 2015; **30**(2): 207-13.
Crowley 2017	43	Crowley MJ, Edelman D, Voils CI, et al. Jump starting shared medical appointments for diabetes with weight management: Rationale and design of a randomized controlled trial. *Contemp Clin Trials* 2017; **58**: 1-12.
Dalziel 2006	44	Dalziel K, Segal L, de Lorgeril M. A mediterranean diet is cost-effective in patients with previous myocardial infarction. *J Nutr* 2006; **136**(7): 1879-85.
Damschroder 2014	45	Damschroder LJ, Lutes LD, Kirsh S, et al. Small-changes obesity treatment among veterans: 12-month outcomes. *Am J Prev Med* 2014; **47**(5): 541-53.
Daubenmier 2016	46	Daubenmier J, Moran PJ, Kristeller J, et al. Effects of a mindfulness-based weight loss intervention in adults with obesity: A randomized clinical trial. *Obesity (Silver Spring)* 2016; **24**(4): 794-804.
Daumit 2013	47	Daumit GL, Dickerson FB, Wang NY, et al. A behavioral weight-loss intervention in persons with serious mental illness. *N Engl J Med* 2013; **368**(17): 1594-602.
deVos 2016	48	de Vos BC, Runhaar J, van Middelkoop M, Krul M, Bierma-Zeinstra SM. Long-term effects of a randomized, controlled, tailor-made weight-loss intervention in primary care on the health and lifestyle of overweight and obese women. *Am J Clin Nutr* 2016; **104**(1): 33-40.
Delahanty 2015	49	Delahanty LM, Dalton KM, Porneala B, et al. Improving diabetes outcomes through lifestyle change--A randomized controlled trial. *Obesity (Silver Spring)* 2015; **23**(9): 1792-9.
Djuric 2002	50	Djuric Z, DiLaura NM, Jenkins I, et al. Combining weight-loss counseling with the weight watchers plan for obese breast cancer survivors. *Obes Res* 2002; **10**(7): 657-65.
Duncan 2016	51	Duncan S, Goodyear-Smith F, McPhee J, Zinn C, Grøntved A, Schofield G. Family-centered brief intervention for reducing obesity and cardiovascular disease risk: A randomized controlled trial. *Obesity (Silver Spring)* 2016; **24**(11): 2311-8.
Eakin 2014	52	Eakin EG, Winkler EA, Dunstan DW, et al. Living well with diabetes: 24-month outcomes from a randomized trial of telephone-delivered weight loss and physical activity intervention to improve glycemic control. *Diabetes care* 2014; **37**(8): 2177-85.

Fernandez-Ruiz 2018	53	Fernández-Ruiz VE, Armero-Barranco D, Paniagua-Urbano JA, Sole-Agusti M, Ruiz-Sánchez A, Gómez-Marín J. Short-medium-long-term efficacy of interdisciplinary intervention against overweight and obesity: Randomized controlled clinical trial. *Int J Nurs Prac* 2018; **24**(6): e12690.
Fisher 2011	54	Fisher G, Hyatt TC, Hunter GR, Oster RA, Desmond RA, Gower BA. Effect of diet with and without exercise training on markers of inflammation and fat distribution in overweight women. *Obesity (Silver Spring)* 2011; **19**(6): 1131-6.
Foley 2016	55	Foley P, Steinberg D, Levine E, et al. Track: A randomized controlled trial of a digital health obesity treatment intervention for medically vulnerable primary care patients. *Contemp Clin Trials* 2016; **48**: 12-20.
Foster-Schubert 2012	56	Foster-Schubert KE, Alfano CM, Duggan CR, et al. Effect of diet and exercise, alone or combined, on weight and body composition in overweight-to-obese postmenopausal women. *Obesity (Silver Spring)* 2012; **20**(8): 1628-38.
Fuller 2012	57	Fuller NR, Lau NS, Denyer G, Caterson ID. A 12-month, randomised, controlled trial to examine the efficacy of the Korean diet in an Australian overweight and obese population - A follow up analysis. *Obes Res Clin Pract* 2012; **6**(4): e263-346.
Green 2015	58	Green CA, Yarborough BJ, Leo MC, et al. Weight maintenance following the STRIDE lifestyle intervention for individuals taking antipsychotic medications. *Obesity (Silver Spring)* 2015; **23**(10): 1995-2001.
Hageman 2017	59	Hageman PA, Pullen CH, Hertzog M, Pozehl B, Eisenhauer C, Boeckner LS. Web-Based Interventions Alone or Supplemented with Peer-Led Support or Professional Email Counseling for Weight Loss and Weight Maintenance in Women from Rural Communities: Results of a Clinical Trial. *J Obes* 2017; **2017**: 1602627.
Hardcastle 2013	60	Hardcastle SJ, Taylor AH, Bailey MP, Harley RA, Hagger MS. Effectiveness of a motivational interviewing intervention on weight loss, physical activity and cardiovascular disease risk factors: a randomised controlled trial with a 12-month post-intervention follow-up. *Int J Behav Nutr Phys Act* 2013; **10**: 40.
Harrigan 2016	61	Harrigan M, Cartmel B, Loftfield E, et al. Randomized Trial Comparing Telephone Versus In-Person Weight Loss Counseling on Body Composition and Circulating Biomarkers in Women Treated for Breast Cancer: The Lifestyle, Exercise, and Nutrition (LEAN) Study. *J Clin Oncol* 2016; **34**(7): 669-76.
Hunt 2014	62	Hunt K, Wyke S, Gray CM, et al. A gender-sensitised weight loss and healthy living programme for overweight and obese men delivered by Scottish Premier League football clubs (FFIT): a pragmatic randomised controlled trial. *Lancet* 2014; **383**(9924): 1211-21.
Irwin 2003	63	Irwin ML, Yasui Y, Ulrich CM, et al. Effect of exercise on total and intra-abdominal body fat in postmenopausal women: a randomized controlled trial. *JAMA* 2003; **289**(3): 323-30.
Jebb 2011	64	Jebb SA, Ahern AL, Olson AD, et al. Primary care referral to a commercial provider for weight loss treatment versus standard care: a randomised controlled trial. *Lancet* 2011; **378**(9801): 1485-92.
Jebb 2017	65	Jebb SA, Astbury NM, Tearne S, Nickless A, Aveyard P. Doctor Referral of Overweight People to a Low-Energy Treatment (DROPLET) in primary care using total diet replacement products: a protocol for a randomised controlled trial. *BMJ open* 2017; **7**(8): e016709.
Jenkins 2017	66	Jenkins DJA, Boucher BA, Ashbury FD, et al. Effect of Current Dietary Recommendations on Weight Loss and Cardiovascular Risk Factors. *J Am Coll Cardiol* 2017; **69**(9): 1103-12.
Katula 2013	67	Katula JA, Vitolins MZ, Morgan TM, et al. The Healthy Living Partnerships to Prevent Diabetes study: 2-year outcomes of a randomized controlled trial. *Am J Prev Med* 2013; **44**(4 Suppl 4): S324-32.
Katzer 2008	68	Katzer L, Bradshaw AJ, Horwath CC, Gray AR, O'Brien S, Joyce J. Evaluation of a "nondieting" stress reduction program for overweight women: a randomized trial. *Am J Health Promot* 2008; **22**(4): 264-74.
King 1989	69	King AC, Frey-Hewitt B, Dreon DM, Wood PD. Diet vs exercise in weight maintenance. The effects of minimal intervention strategies on long-term outcomes in men. *Arch Intern Med* 1989; **149**(12): 2741-6.
Knauper 2018	70	Knäuper B, Carrière K, Frayn M, et al. The Effects of If-Then Plans on Weight Loss: Results of the McGill CHIP Healthy Weight Program Randomized Controlled Trial. *Obesity (Silver Spring)* 2018; **26**(8): 1285-95.
Diabetes Prevention Program R G 2009	71	Knowler WC, Fowler SE, Hamman RF, et al. 10-year follow-up of diabetes incidence and weight loss in the Diabetes Prevention Program Outcomes Study. *Lancet* 2009; **374**(9702): 1677-86.<br><br>Diabetes Prevention Program Research Group. Long-term effects of lifestyle intervention or metformin on diabetes development and microvascular complications over 15-year follow-up: the Diabetes Prevention Program Outcomes

		Study. The Lancet Diabetes & Endocrinology. 2015;3(11):866-875. doi:10.1016/S2213-8587(15)00291-0
Kuller 2012	72	Kuller LH, Pettee Gabriel KK, Kinzel LS, et al. The Women on the Move Through Activity and Nutrition (WOMAN) study: final 48-month results. *Obesity (Silver Spring)* 2012; **20**(3): 636-43.
Kumanyika 2012	73	Kumanyika SK, Fassbender JE, Sarwer DB, et al. One-year results of the Think Health! study of weight management in primary care practices. *Obesity (Silver Spring)* 2012; **20**(6): 1249-57.
Ley 2004	74	Ley SJ, Metcalf PA, Scragg RK, Swinburn BA. Long-term effects of a reduced fat diet intervention on cardiovascular disease risk factors in individuals with glucose intolerance. *Diabetes Res Clin Pract* 2004; **63**(2): 103-12.
Li 2016	75	Li X, Cai X, Ma X, et al. Short- and Long-Term Effects of Wholegrain Oat Intake on Weight Management and Glucolipid Metabolism in Overweight Type-2 Diabetics: A Randomized Control Trial. *Nutrients* 2016; **8**(9).
Li 2005	76	Li Z, Hong K, Saltsman P, et al. Long-term efficacy of soy-based meal replacements vs an individualized diet plan in obese type II DM patients: relative effects on weight loss, metabolic parameters, and C-reactive protein. *Eur J Clin Nutr* 2005; **59**(3): 411-8
Lindstrom 2003	77	Lindström J, Louheranta A, Mannelin M, et al. The Finnish Diabetes Prevention Study (DPS): Lifestyle intervention and 3-year results on diet and physical activity. *Diabetes care* 2003; **26**(12): 3230-6.<br><br>Additional reference to this study in the main paper: 21. Lindström J, Peltonen M, Eriksson JG, Ilanne-Parikka P, Aunola S, Keinänen-Kiukaanniemi S, Uusitupa M, Tuomilehto J. Improved lifestyle and decreased diabetes risk over 13 years: long-term follow-up of the randomised Finnish Diabetes Prevention Study (DPS). Diabetologia. 2013 Feb;56(2):284-93. eng. Epub 2012/10/25. doi:10.1007/s00125-012-2752-5. Cited in: Pubmed; PMID 23093136.
Liss 2016	78	Liss DT, Finch EA, Gregory DL, Cooper A, Ackermann RT. Design and participant characteristics for a randomized effectiveness trial of an intensive lifestyle intervention to reduce cardiovascular risk in adults with type 2 diabetes: The I-D-HEALTH study. *Contemp Clin Trials* 2016; **46**: 114-21.
Little 2016	79	Little P, Stuart B, Hobbs FR, et al. An internet-based intervention with brief nurse support to manage obesity in primary care (POWeR+): a pragmatic, parallel-group, randomised controlled trial. *Lancet Diabetes Endocrinol* 2016; **4**(10): 821-8.
Manning 1994	80	Manning RM, Jung RT, Leese GP, Newton RW. The comparison of four weight reduction strategies aimed at overweight diabetic patients. *Diabet Med*; **12**(5): 409-15.
Mefferd 2007	81	Mefferd K, Nichols JF, Pakiz B, Rock CL. A cognitive behavioral therapy intervention to promote weight loss improves body composition and blood lipid profiles among overweight breast cancer survivors. *Breast Cancer Res Treat* 2007; **104**(2): 145-52.
Melchart 2017	82	Melchart D, Löw P, Wühr E, Kehl V, Weidenhammer W. Effects of a tailored lifestyle self-management intervention (TALENT) study on weight reduction: a randomized controlled trial. *Diabetes Metab Syndr Obes* 2017; **10**: 235-45.
Melin 2003	83	Melin I, Karlström B, Lappalainen R, Berglund L, Mohsen R, Vessby B. A programme of behaviour modification and nutrition counselling in the treatment of obesity: a randomised 2-y clinical trial. *Int J Obes Relat Metab Disord* 2003; **27**(9): 1127-35.
Menard 2005	84	Ménard J, Payette H, Baillargeon JP, et al. Efficacy of intensive multitherapy for patients with type 2 diabetes mellitus: a randomized controlled trial. *CMAJ* 2005; **173**(12): 1457-66.
Mengham 1999	85	Mengham L, Morris B, Palmer C, White A. Is intensive dietetic intervention effective for overweight patients with diabetes mellitus? A randomized controlled study in a general practice. *Pract Diab Int* 1999; **16**(1): 5-8.
Mensinger 2016	86	Mensinger JL, Calogero RM, Stranges S, Tylka TL. A weight-neutral versus weight-loss approach for health promotion in women with high BMI: A randomized-controlled trial. *Appetite* 2016; **105**: 364-74.
Mitsui 2008	87	Mitsui T, Shimaoka K, Tsuzuku S, Kajioka T, Sakakibara H. Gentle exercise of 40 minutes with dietary counseling is effective in treating metabolic syndrome. *Tohoku J Exp Med* 2008; **215**(4): 355-61
Moreno 2014	88	Moreno B, Bellido D, Sajoux I, et al. Comparison of a very low-calorie-ketogenic diet with a standard low-calorie diet in the treatment of obesity. *Endocrine* 2014; **47**(3): 793-805.
Morgan 2010	89	Morgan PJ, Lubans DR, Collins CE, Warren JM, Callister R. 12-month outcomes and process evaluation of the SHED-IT RCT: an internet-based weight loss program targeting men. *Obesity (Silver Spring)* 2011; **19**(1): 142-51.
Muggia 2014	90	Muggia C, Falchi AG, Michelini I, et al. Brief group cognitive behavioral treatment in addition to prescriptive diet

		versus standard care in obese and overweight patients. A randomized controlled trial. *e-SPEN* 2014; **9**(1): e26-e33.
Nakata 2014	91	Nakata Y, Okada M, Hashimoto K, Harada Y, Sone H, Tanaka K. Weight loss maintenance for 2 years after a 6-month randomised controlled trial comparing education-only and group-based support in Japanese adults. *Obes Facts* 2014; **7**(6): 376-87
Nanchahal 2012	92	Nanchahal K, Power T, Holdsworth E, et al. A pragmatic randomised controlled trial in primary care of the Camden Weight Loss (CAMWEL) programme. *BMJ open* 2012; **2**(3).
Ng 2015	93	Ng SSS, Chan RSM, Woo J, et al. A Randomized Controlled Study to Examine the Effect of a Lifestyle Modification Program in OSA. *Chest* 2015; **148**(5): 1193-203.
Nilsen 2011	94	Nilsen V, Bakke PS, Gallefoss F. Effects of lifestyle intervention in persons at risk for type 2 diabetes mellitus - results from a randomised, controlled trial. *BMC public health* 2011; **11**: 893.
Nordby 2012	95	Nordby P, Auerbach PL, Rosenkilde M, et al. Endurance training per se increases metabolic health in young, moderately overweight men. *Obesity (Silver Spring)* 2012; **20**(11): 2202-12.
Oldroyd 2006	96	Oldroyd JC, Unwin NC, White M, Mathers JC, Alberti KG. Randomised controlled trial evaluating lifestyle interventions in people with impaired glucose tolerance. *Diabetes Res Clin Pract* 2006; **72**(2): 117-27.
Pan 1997	97	Pan XR, Li GW, Hu YH, et al. Effects of diet and exercise in preventing NIDDM in people with impaired glucose tolerance. The Da Qing IGT and Diabetes Study. *Diabetes Care* 1997; **20**(4): 537-44.
Parikh 2010	98	Parikh P, Simon EP, Fei K, Looker H, Goytia C, Horowitz CR. Results of a pilot diabetes prevention intervention in East Harlem, New York City: Project HEED. *Am J Public Health* 2010; **100 Suppl 1**(Suppl 1): S232-9.
Pedersen 2013	99	Pedersen LR, Olsen RH, Frederiksen M, et al. Copenhagen study of overweight patients with coronary artery disease undergoing low energy diet or interval training: the randomized CUT-IT trial protocol. *BMC Cardiovasc Disord* 2013; **13**: 106.
Pettman 2009	100	Pettman TL, Buckley JD, Misan GM, Coates AM, Howe PR. Health benefits of a 4-month group-based diet and lifestyle modification program for individuals with metabolic syndrome. *Obes Res Clin Pract* 2009; **3**(4): 221-35.
Promrat 2010	101	Promrat K, Kleiner DE, Niemeier HM, et al. Randomized controlled trial testing the effects of weight loss on nonalcoholic steatohepatitis. *Hepatology* 2010; **51**(1): 121-9.
Provencher 2009	102	Provencher V, Bégin C, Tremblay A, et al. Health-At-Every-Size and eating behaviors: 1-year follow-up results of a size acceptance intervention. *J Am Diet Assoc* 2009; **109**(11): 1854-61.
Ridgeway 1999	103	Ridgeway NA, Harvill DR, Harvill LM, Falin TM, Forester GM, Gose OD. Improved control of type 2 diabetes mellitus: a practical education/behavior modification program in a primary care clinic. *South Med J* 1999; **92**(7): 667-72.
Rock 2015	104	Rock CL, Flatt SW, Byers TE, et al. Results of the Exercise and Nutrition to Enhance Recovery and Good Health for You (ENERGY) Trial: A Behavioral Weight Loss Intervention in Overweight or Obese Breast Cancer Survivors. *J Clin Oncol* 2015; **33**(28): 3169-76.
Rolls 2005	105	Rolls BJ, Roe LS, Beach AM, Kris-Etherton PM. Provision of foods differing in energy density affects long-term weight loss. *Obes Res* 2005; **13**(6): 1052-60.
Rolls 2017	106	Rolls BJ, Roe LS, James BL, Sanchez CE. Does the incorporation of portion-control strategies in a behavioral program improve weight loss in a 1-year randomized controlled trial? *Int J Obes (Lond)* 2017; **41**(3): 434-42.
Rosas 2015	107	Rosas LG, Thiyagarajan S, Goldstein BA, et al. The effectiveness of two community-based weight loss strategies among obese, low-income US Latinos. *J Acad Nutr Diet* 2015; **115**(4): 537-50.e2.
Ross 2012	108	Ross R, Lam M, Blair SN, et al. Trial of prevention and reduction of obesity through active living in clinical settings: a randomized controlled trial. *Arch Intern Med* 2012; **172**(5): 414-24.
Samaras 1997	109	Samaras K, Ashwell S, Mackintosh AM, Fleury AC, Campbell LV, Chisholm DJ. Will older sedentary people with non-insulin-dependent diabetes mellitus start exercising? A health promotion model. *Diabetes Res Clin Pract* 1997; **37**(2): 121-8.
Sattin 2016	110	Sattin RW, Williams LB, Dias J, et al. Community Trial of a Faith-Based Lifestyle Intervention to Prevent Diabetes Among African-Americans. *J Community Health* 2016; **41**(1): 87-96.

Schubel 2016	111	Schübel R, Graf ME, Nattenmüller J, et al. The effects of intermittent calorie restriction on metabolic health: Rationale and study design of the HELENA Trial. *Contemp Clin Trials* 2016; **51**: 28-33.
Seligman 2011	112	Seligman BG, Polanczyk CA, Santos AS, et al. Intensive practical lifestyle intervention improves endothelial function in metabolic syndrome independent of weight loss: a randomized controlled trial. *Metabolism* 2011; **60**(12): 1736-40.
Shikany 2013	113	Shikany JM, Thomas AS, Beasley TM, Lewis CE, Allison DB. Randomized controlled trial of the Medifast 5 & 1 Plan for weight loss. *Int J Obes (Lond)* 2013; **37**(12): 1571-8.
Snel 2012	114	Snel M, Sleddering MA, Vd Peijl ID, et al. Quality of life in type 2 diabetes mellitus after a very low calorie diet and exercise. *Eur J Intern Med* 2012; **23**(2): 143-9.
Stevens 1993	115	Stevens VJ, Corrigan SA, Obarzanek E, et al. Weight loss intervention in phase 1 of the Trials of Hypertension Prevention. The TOHP Collaborative Research Group. *Arch Intern Med* 1993; **153**(7): 849-58.
Stevens 2001	116	Stevens VJ, Obarzanek E, Cook NR, et al. Long-term weight loss and changes in blood pressure: results of the Trials of Hypertension Prevention, phase II. *Ann Intern Med* 2001; **134**(1): 1-11.
Sundfor 2018	117	Sundfør TM, Svendsen M, Tonstad S. Effect of intermittent versus continuous energy restriction on weight loss, maintenance and cardiometabolic risk: A randomized 1-year trial. *Nutr Metab Cardiovasc Dis* 2018; **28**(7): 698-706.
Tapsell 2017	118	Tapsell LC, Lonergan M, Batterham MJ, et al. Effect of interdisciplinary care on weight loss: a randomised controlled trial. *BMJ open* 2017; **7**(7): e014533.
TarragaMarcos 2017	119	Tárraga Marcos ML, Panisello Royo JM, Carbayo Herencia JA, Rosich Domenech N, Alins Presas J, Tárraga López PJ. Effect on the lipid parameters of an intervention to reduce weight in overweight and obese patients. *Clin Investig Arterioscler* 2017; **29**(3): 103-10.
Teeriniemi 2018	120	Teeriniemi AM, Salonurmi T, Jokelainen T, et al. A randomized clinical trial of the effectiveness of a Web-based health behaviour change support system and group lifestyle counselling on body weight loss in overweight and obese subjects: 2-year outcomes. *J Intern Med* 2018; **284**(5): 534-45.
ter Bogt 2009	121	ter Bogt NC, Bemelmans WJ, Beltman FW, Broer J, Smit AJ, van der Meer K. Preventing weight gain: one-year results of a randomized lifestyle intervention. *Am J Prev Med* 2009; **37**(4): 270-7.
Tsai 2010	122	Tsai AG, Wadden TA, Rogers MA, Day SC, Moore RH, Islam BJ. A primary care intervention for weight loss: results of a randomized controlled pilot study. *Obesity (Silver Spring)* 2010; **18**(8): 1614-8.
Tuomilehto 2009	123	Tuomilehto HP, Seppä JM, Partinen MM, et al. Lifestyle intervention with weight reduction: first-line treatment in mild obstructive sleep apnea. *Am J Respir Crit Care Med* 2009; **179**(4): 320-7.
van de Glind 2017	124	van de Glind I, Bunn C, Gray CM, et al. The intervention process in the European Fans in Training (EuroFIT) trial: a mixed method protocol for evaluation. *Trials* 2017; **18**(1): 356.
vanWier 2011	125	van Wier MF, Dekkers JC, Hendriksen IJ, et al. Effectiveness of phone and e-mail lifestyle counseling for long term weight control among overweight employees. *J Occup Environ Med* 2011; **53**(6): 680-6.
Vissers 2010	126	Vissers D, Verrijken A, Mertens I, et al. Effect of long-term whole body vibration training on visceral adipose tissue: a preliminary report. *Obes Facts* 2010; **3**(2): 93-100.
Volpe 2008	127	Volpe SL, Kobusingye H, Bailur S, Stanek E. Effect of diet and exercise on body composition, energy intake and leptin levels in overweight women and men. *J Am Coll Nutr* 2008 **27**(2): 195-208.
Weinstock 2013	128	Weinstock RS, Trief PM, Cibula D, Morin PC, Delahanty LM. Weight loss success in metabolic syndrome by telephone interventions: results from the SHINE Study. *J Gen Intern Med* 2013; **28**(12): 1620-8.
West 2007	129	West DS, DiLillo V, Bursac Z, Gore SA, Greene PG. Motivational interviewing improves weight loss in women with type 2 diabetes. *Diabetes Care* 2007; **30**(5): 1081-7.
The Look AHEAD Research Group 2010	130	Wing RR. Long-term effects of a lifestyle intervention on weight and cardiovascular risk factors in individuals with type 2 diabetes mellitus: four-year results of the Look AHEAD trial. *Arch Intern Med* 2010; **170**(17): 1566-75.
Wing 1988	131	Wing RR, Epstein LH, Paternostro-Bayles M, Kriska A, Nowalk MP, Gooding W. Exercise in a behavioural weight control programme for obese patients with Type 2 (non-insulin-dependent) diabetes. *Diabetologia* 1988; **31**(12): 902-9.<br>*One publication which reports two studies.*

Wing 1988b	132	Wing RR, Epstein LH, Paternostro-Bayles M, Kriska A, Nowalk MP, Gooding W. Exercise in a behavioural weight control programme for obese patients with Type 2 (non-insulin-dependent) diabetes. *Diabetologia* 1988; **31**(12): 902-9.<br>*One publication which reports two studies.*
Wing 1991	132	Wing RR, Marcus MD, Salata R, Epstein LH, Miaskiewicz S, Blair EH. Effects of a very-low-calorie diet on long-term glycemic control in obese type 2 diabetic subjects. *Arch Intern Med* 1991; **151**(7): 1334-40.
Wing 1998	133	Wing RR, Venditti E, Jakicic JM, Polley BA, Lang W. Lifestyle intervention in overweight individuals with a family history of diabetes. *Diabetes Care* 1998; **21**(3): 350-9.
Yannakoulia 2008	134	Yannakoulia M, Poulia KA, Mylona E, Kontogianni MD. Effectiveness of an intensive nutritional intervention in patients with type 2 diabetes mellitus: results from a pilot study. *Rev Diabet Stud* 2007; **4**(4): 226-30.
Yates 2009	135	Yates T, Davies M, Gorely T, Bull F, Khunti K. Effectiveness of a pragmatic education program designed to promote walking activity in individuals with impaired glucose tolerance: a randomized controlled trial. *Diabetes Care* 2009; **32**(8): 1404-10.
Yeh 2016	136	Yeh MC, Heo M, Suchday S, et al. Translation of the Diabetes Prevention Program for diabetes risk reduction in Chinese immigrants in New York City. *Diabet Med* 2016; **33**(4): 547-51.
Yin 2018	137	Yin Z, Perry J, Duan X, et al. Cultural adaptation of an evidence-based lifestyle intervention for diabetes prevention in Chinese women at risk for diabetes: results of a randomized trial. *Int Health* 2018; **10**(5): 391-400.
Zhang 2016	138	Zhang HJ, He J, Pan LL, et al. Effects of Moderate and Vigorous Exercise on Nonalcoholic Fatty Liver Disease: A Randomized Clinical Trial. *JAMA Internal Med* 2016; **176**(8): 1074-82.
*Ref No. Reference number in main paper.		

Table S2. Risk of bias assessments summary
*Only assessed where suspected, as per Cochrane guidance

Risk of bias domain	Number of studies (n = 124)		
	Low risk	Unclear risk	High risk
Overall risk of bias	33	64	27
Selection bias (random sequence generation and allocation concealment)	50	73	1
Detection bias	112	10	2
Attrition bias	108	4	12
Other risk of bias*	-	-	13

Table S3. Risk of bias of included studies
*Ref No. Reference number in main paper.
BP: Blood pressure; **HDL-C:** High density lipoprotein cholesterol; **Mths:** Months; **NS:** Not specified; **PR**: Pulse rate; **RCT**: Randomised controlled trial; **Wk/s**: week/s; **Yr.:** Year; **Yrs:** Years.

Ref No*	Study ID	Random sequence generation (Selection bias)	Allocation concealment (Selection bias)	Blinding of outcome assessment (Detection bias)	Incomplete outcome data (Attrition bias)	Other bias
16	**Abed 2013**	UNCLEAR	UNCLEAR	LOW	LOW	HIGH
	Assessment justification:	Single-centre, partially blinded RCT.	No explicit information regarding allocation concealment provided:<br><br>Study coordinators, treating physicians, and other personnel, with the exception of weight loss counselors, were blinded to randomization.<br>Patients were instructed not to disclose their status.<br>Patient records contained generic statements without indicating group allocation.	Weight objectively measured.	At 12 months, 109 (73%) had completed the study (57 in the intervention group and 52 in the control group). By 15 months, 81 (54%) remained (42 in the intervention group and 39 in the control group).	Weight data at 3, 6, 9 and 12 months from a sub-study (Abed 2015). 87 participants agreed to CMR (cardiac magnetic resonance) imaging (43 in control group, 44 in intervention group at baseline). 69 participants had baseline and 12-month follow-up (33 in control group, 36 in intervention group at 12 months).
17	**Ackermann 2011**	UNCLEAR	UNCLEAR	LOW	LOW	
	Assessment justification:	"matched-pair, group-randomized pilot intervention trial involving two YMCA facilities in greater Indianapolis."<br>No further information provided regarding matching.	NS	Body weight was measured using a calibrated, beam-balanced scale with participants wearing light clothing and no shoes.	Less than 50% attrition at 12-month follow-up.	
18	**Ahern 2017**	LOW	LOW	LOW	LOW	

	Assessment justification:	"The randomisation sequence was generated by the trial statistician and allocates participants in a 2:5:5 allocation stratified by centre and gender, with a block size of 12."	" The sequence is unknown to research staff and participants."	Weight objectively measured.<br>"Weight and fat mass will be measured in kg using a Tanita segmental body composition analyser."	3-months retention rate:<br>Brief intervention: 68%;<br>12-week programme: 76.4%;<br>52-week programme: 86.2%;<br><br>12-month retention rate:<br>Brief intervention: 58.7%;<br>12-week programme: 63.9%;<br>52-week programme: 68.2%;<br><br>24-month retention rate:<br>Brief intervention: 63%;<br>12-week programme: 67%;<br>52-week programme: 69.7%;	
19	**Almanza -Aguilera 2018**	UNCLEAR	UNCLEAR	LOW	HIGH	
	Assessment justification:	Participants were randomly allocated to either the control or the treatment group.<br><br>No further information given.	NS	"Anthropometric measurements, including weight, height, waist circumference (WC), and BMI, were taken by trained nurses..."	"Of the 115 participants recruited, 58 were excluded due to dropout or failure to show at all visits (n = 43), illness (n = 6), unavailable sample at some time point (at baseline, 3 or 12 months, n = 7), or change of residence (n = 2). Therefore, 57 participants were included in the present data analyses." Control n = 27 analysed out of 48 randomised; Treatment n = 30 analysed out of 67 (44.7% retained).	
20	**Andersen 1999**	UNCLEAR	UNCLEAR	LOW	LOW	
	Assessment justification:	"Participants were randomly assigned to 1 of the 2 conditions described above."<br>No further information given.	NS	Weight objectively measured.<br><br>Where a subjective component potentially existed (e.g. measurement of aerobic fitness), tester was blinded.	40 randomized<br>38 completed 16-week follow-up;<br>33 completed 68-week follow-up.	
21	**Anderson 2014**	LOW	LOW	LOW	LOW	

	Assessment justification:	"A statistician, independent of the analysis of study outcomes, had previously generated a randomisation list (site specific identification numbers and group allocation) by using a permuted block technique, with block sizes of four and eight, stratified by trial site."	"This list was emailed to the study administrator and trial manager. Research nurses allocated participants a site specific identification number sequentially and notified the study administrator on completion of baseline measures for each participant. The study administrator then identified the participant's group allocation from the randomisation list and notified the lifestyle counsellor of participants allocated to the intervention group or sent the weight loss booklet to participants allocated to usual care."	Weight objectively measured.<br>"The study team, including the research nurses, were blinded to the participant's group allocation until completion of the primary outcome analysis. Exceptions were the trial manager, study administrator, lifestyle counsellors, and participants who could not be blinded owing to the nature of the intervention. None of these unblinded staff had a role in data analysis."	"The remaining 329 were randomised (163 to intervention, 166 to control). At three months 314 (94% intervention, 97% control) participants had completed the primary outcome measures, and 305 (91% intervention, 95% control) completed the trial at 12 months (93%)."	
22	**Appel 2011**	LOW	LOW	LOW	LOW	
	Assessment justification:	"Randomization was stratified according to sex and was generated in blocks of 3 and 6 with the use of a Web-based program."	Web-based program.	"Participants were asked to make in-person follow-up visits 6, 12, and 24 months after randomization. At each of these visits, weight was measured on a high-quality, calibrated digital scale, with the participant wearing light, indoor clothes and no shoes."	6-month follow-up: Control: 113/138*100= 81.9%;<br>Remote: 129/139*100 = 92.8%;<br>In-person: 124/138*100 = 89.9%<br><br>12-month follow-up: Control: 108/138*100= 78.3%;<br>Remote: 124/139*100 = 89.2%;<br>In-person: 123/138*100 = 89.1%<br><br>24-month follow-up : Control: 129/138*100= 93.5%;<br>Remote: 132/139*100 = 95%;<br>In-person: 133/138*100 = 96.4%	
23	**Ard 2018**	LOW	LOW	LOW	LOW	
	Assessment justification:	The statistician generated blocked random assignments using a computer-based algorithm, stratified by age category (65–74, 75+), sex, and race.	Allocations were concealed in sealed envelopes that were opened by a research assistant at the time of randomization.	Body weight was measured in light clothing on calibrated electronic scales to the nearest 0.1 pound and converted to kilograms.	Less than 50% attrition.	

24	**Ard 2004**	LOW	LOW	LOW	LOW	
	Assessment justification:	Randomization assignments were made centrally by a computer program. Assignments were stratified by clinic and hypertension status; the randomization block size was 24.	Randomization assignments were made centrally by a computer program.	Weight was measured using a calibrated scale.	Less than 25% attrition at 6-month and 18-months follow-up.	
25	**Ashley 2001**	UNCLEAR	UNCLEAR	LOW	HIGH	
	Assessment justification:	NS	NS	Weight objectively measured.<br>Certified technicians took blood pressure and body composition measurements. Fasting blood was taken for measuring serum lipids (total cholesterol, low density lipoprotein [LDL] cholesterol, high-density lipoprotein [HDL] cholesterol, and triglycerides), glucose, and insulin by a certified phlebotomist. Blood values were analyzed by standard methods at a statewide, certified clinical laboratory.	12-months:<br>74/113 completed all assessments: LOW<br>24-months:<br>39/113 completed all assessments: HIGH	
26	**Azar 2013**	LOW	LOW	LOW	LOW	
	Assessment justification:	Participants are randomized on a 1:1:1 basis to one of three arms: UC, SM, or CM. Pocock's "minimization" procedure is used to assure better than chance group balance with respect to participant age, gender, race, BMI, fasting blood glucose, waist circumference, and use of PAMFOnline, which is PAMF's online patient portal to access his or her own health record (user vs. non-user). For each participant about to be randomized, a computerized randomization algorithm automatically calculates an imbalance score for each of the balancing factors, as the excess or deficit of previously randomized participants in each arm matching the current patient on that factor. These scores are summed over factors to form a total imbalance score, S, for each treatment arm. The randomization	A designated research staff member who is not involved in follow-up data collection or data analysis assigns each study arm a non-revealing label, e.g., A, B, or C, and performs actual randomization of the participants.	Weight objectively measured.	171/241 participants at 24-months.	

		probability of assigning the patient to the treatment associated with the smallest S is set to 2/3, and the other two treatments are each assigned a probability of 1/6 based on Efron's biased coin method.				
27	**Bacon 2002**	UNCLEAR	UNCLEAR	LOW	LOW	
	Assessment justification:	"To ensure balance in the treatment groups, the enrolled subjects (n = 78) were divided into BMI quartiles, and high/ low sets for dietary restraint, 34 degrees of flexible and rigid control of eating, 35 age, and self-reported activity level. The subjects in these subgroups were then randomly assigned to one of two treatment groups."	NS	Weight objectively measured.<br>Blood pressure was assessed in duplicate using the oscillometric technique. Fasting blood samples were analyzed for blood lipids (total cholesterol, low-density lipoprotein [LDL] cholesterol, and high-density lipoprotein [HDL] cholesterol).	52-weeks:<br>Diet group: 23/39 completed testing;<br>HAES group: 34 attended (29 completed testing)/36	
28	**Barnes 2017**	UNCLEAR	UNCLEAR	LOW	LOW	
	Assessment justification:	Participants were randomly assigned, stratified by BED diagnosis, to one of three conditions.<br><br>No further information given.	NS	Weight objectively measured.	Less than 50% attrition at 12-month follow-up.	
29	**Bartels 2015**	LOW	UNCLEAR	LOW	LOW	
	Assessment justification:	"Randomization between In SHAPE and the comparison condition was stratified by age (21 to 44 years versus 45 years and older) and psychiatric diagnosis (mood disorders versus schizophrenia spectrum disorders). Each combination of stratification categories had its own randomization schedule that was blocked on every fourth assignment to ensure balance between treatment arms. Randomization was conducted sequentially across all sites (not within sites)."	NS	Weight objectively measured.<br><br>'Blood pressure was measured before (resting heart rate) and after completing the 6-MWT'<br><br>'Lipids were measured using the CardioChek PA Analyzer, a portable testing system that produces reliable values for total cholesterol, LDL, HDL, and triglycerides using a multi-panel test strip and a single drop of blood acquired with a finger prick.'	18-months:<br>Control: 83/106 Intervention: 80/104	
30	**Beeken 2017**	LOW	LOW	LOW	LOW	

	Assessment justification:	"A computer-generated list of random permuted blocks of size 2–4 was used. Randomisation was stratified by PCP to ensure socioeconomic balance between groups."	"A central telephone-based randomisation service was used to randomise at the level of the patient ensuring allocation concealment"	"All measurements at 3 months were with a health professional blind to group allocation."<br><br>Weight objectively measured.	At 12-months, 61% (Control) and 57% (10TT) were followed up.<br>"At 24 months, 312 (58.1%) patients were followed up. There remained very little difference in attrition between arms (41.5% in the usual care group vs 42.3% in the 10TT group)."	
31	**Bennett 2013**	LOW	UNCLEAR	LOW	LOW	
	Assessment justification:	A computer-generated randomization algorithm to allocate participants equally (1:1) across the 2 treatment arms (intervention and usual care); those in the intervention arm were further randomized to 1 of 2 interventionists.	NS	Weight objectively measured.<br>Secondary measures included waist circumference, blood pressure, and fasting glucose, triglyceride, and cholesterol level.	Usual care: 90/97<br>Intervention: 86/97	
32	**Bennett 2012**	LOW	UNCLEAR	LOW	LOW	
	Assessment justification:	Participants were randomized to treatment arm using computer-generated allocations, blocked by clinic and sex.	NS	Weight objectively measured.	24-months:<br>Usual care: 166/185<br>Intervention: 148/180	
33	**Bertz 2012**	LOW	UNCLEAR	LOW	LOW	
	Assessment justification:	Random number table	Allocation method not reported but described as 'concealed'.	Weight objectively measured.<br>Body composition was measured by using dual-energy X-ray absorptiometry (DXA) (Lunar Prodigy; GE Lunar Corp). Muscle mass was calculated from DXA.	92% followed up at 12-months, intervention 100%, D 76%, E 83%, control 76%. 4 missing (6%); 2 medical reasons (3%).	
34	**Bo 2007**	LOW	LOW	LOW	LOW	
	Assessment justification:	"The randomization procedure was automatically performed by a statistician using an SAS program developed to minimize the differences between the two groups for all stratifying variables. The patients were randomly allocated to receive either standard lifestyle recommendations from their physicians (control group, n=188) or a structured lifestyle intervention program for 1 year carried out by health professionals (intervention group, n=187)."	"Random allocation with a minimization algorithm was centrally performed in a single step. The researchers then received the two lists of nominative data. The possibility for researchers to predict or influence the allocation of participants was thus completely prevented."	Weight, waist circumference, and blood pressure were measured. Fasting glucose, insulin, triglycerides, high-density lipoprotein (HDL) cholesterol, uric acid, and hs-CRP values were measured before and after the study in both groups.	12-months:<br>Control: 166/188<br>Intervention: 169/187	

35	**Burke 2005**	LOW	UNCLEAR	LOW	LOW	
	Assessment justification:	Random allocation - computer-generated random numbers. Random allocation to groups was stratified by age and BMI and used a block size of 4.	NS	Weight objectively measured.	Control:<br>98/118 at 4 months; 90/118 at 1 year follow up (16 months); 64/118 at 3 year follow up (40 months).<br>Intervention:<br>106/123 at 4 months; 102/123 at 16 months; 76/123 at 40 months.	
36	**Chaiyasoot 2018**	LOW	LOW	LOW	LOW	
	Assessment justification:	Randomly assigned (1:1 allocation) to receive either LEI or LEI + MR by a computer generated block randomisation.	"Opaque concealed envelopes were drawn by independent personnel who was not involved in the study to ensure allocation concealment. Neither the investigators nor the participants were blinded to the group allocation due to the nature of the intervention."	Weight objectively measured.<br><br>BP and PR were obtained using an electronic sphygmomanometre (Terumo Elemano [ES-H55], Medaval, New Jersey, United States) in a comfortable sitting position after at least 15-min rest. Blood sampling was undertaken following a 12-h overnight fast. TC, HDL-c, LDL-c, TG, glucose, insulin, urine microalbumin and urine creatinine were analysed with a biochemical autoanalyser (Cobas® 8000 Modular Analyser Series, Roche Diagnostics, Indianapolis, United States). HbA1c was determined using Cobas Integra® 800 analyser, Roche Diagnostics, Indianapolis, United States. Homeostatic model assessment of insulin resistance (HOMA-IR) was calculated as: HOMA-IR = (FPG × fasting insulin)/22.5 in molar units.	LEI:<br>45/52 at 12-weeks; 44/52 at 64-weeks.<br>LEI + MR:<br>48/58 at 12-weeks;<br>42/58 at 64-weeks.	
37	**Chee 2017**	LOW	UNCLEAR	LOW	LOW	
	Assessment justification:	Patients were randomized using a random allocation software.	NS	Weight objectively measured.	UC:<br>3mth: 105/115;<br>6mth: 101/115;<br>9mth: 99/115;	

					12mth 98/115<br><br>tDNA-CC:<br>3mth: 48/57;<br>6mth: 40/57;<br>9mth: 40/57;<br>12mth: 40/57<br><br>tDNA-MI:<br>3mth: 51/58;<br>6mth: 51/58;<br>9mth: 51/58;<br>12mth: 51/58.	
38	**Cheskin 2008**	LOW	UNCLEAR	LOW	HIGH	
	Assessment justification:	Random-number generator.	Allocation revealed when the participants were assigned to a group by the study coordinator (after randomisation)	Weight objectively measured.	At 34-weeks:<br>standard diet 17/58<br>PCD 31/54<br><br>At 86-weeks:<br>Standard diet 8/58<br>PCD 16/54	
39	**Cheyette 2007**	UNCLEAR	UNCLEAR	LOW	LOW	
	Assessment justification:	"Non probability volunteer sampling was used to assign people to either the intervention or the control group."	NS	Weight objectively measured.	" At six and 12 months follow up a total of eight people dropped out from the intervention group and two from the control group."	
40	**Christensen 2012**	LOW	LOW	LOW	LOW	LOW
	Assessment justification:	Cluster-randomization procedure. A cluster formation of the groups was performed to assure equal allocation in the intervention and reference groups balanced on sex, age, job seniority or job type with cluster size varying from 3 to 15.	The randomization was done by an external research group, which had no knowledge of the work place or the participants. Clusters were randomly allocated to intervention and control by the drawing of sealed envelopes from a bag.	The test manager was blinded regarding the participants intervention status, and whenever possible the same test manager tested the subject at all three rounds of tests	98 participants --> 83 participants.	Clusters were created based on information from the screening questionnaire and the management of working teams, day and evening/night shifts and close working relations. This approach was chosen to avoid contamination, and to benefit from the social support in work teams, thereby in- creasing compliance.

41	**Cole 2013**	LOW	LOW	LOW	UNCLEAR	
	Assessment justification:	"Randomization occurred by a computer-generated random-numbers list (SPSS version 15.0.1; IBM Corporation) with assignments placed in sealed envelopes, numbered sequentially, and allocated to participants in the order of recruitment."	Sealed envelopes used.	Weight objectively measured.	"…94 were randomized into the 2 study groups, 80% remained at 3 months, and 69% completed the 1-year assessment (n = 34 SMA, n = 31 control, n = 29 lost to follow-up)."<br><br>"Limitations of the study resulted from a high attrition (31%)."<br><br>Data only given at all points for those who completed year 1 – loss to follow-up from each group is unclear.	
42	**Conroy 2015**	UNCLEAR	LOW	UNCLEAR	LOW	
	Assessment justification:	"…randomization occurred in a 1:1 allocation. Each woman was allowed to draw a sealed envelope that contained a designation assignment, either interventionist-led (IL) or self-guided (SG)."	Sealed envelopes.	Weight was measured by a trained staff member in clinic using a standard balance beam scale (SECA Medichoice) and following a written protocol. " For the 12-month followup, 62 (74 %) of 84 participating women had an in-person assessment (with study-measured weight), with the remainder of the outcomes assessed by phone." Breakdown by group not clear	"Follow-up was better in the IL group (90 % at 3 months and 96 % at 12 months) than in the SG group (63 % at 3 months and 76 % at 12 months), but otherwise did not differ by other participant characteristics."	
43	**Crowley 2017**	LOW	LOW	LOW	LOW	HIGH
	Assessment justification:	"Eligible participants were randomized using a computerized random number generator in blocks of 2 (study personnel other than statisticians blinded to block size) within strata defined by baseline HbA1c level (7.5%-8.9% vs ≥9%) and insulin use (multiple types vs 1 type or none)."	After a patient's screening information has been reviewed and found to meet eligibility criteria, the study coordinator will access a computer program in which to enter the values of the stratification variables; in turn, the computer program will provide the participant's randomly assigned study arm: WM/SMA or SMA.	Body weight was measured at every visit using a standardized digital scale.	222/263 at 16-weeks; 198/263 at 32-weeks; 209/263 at 48-weeks<br><br>GMV:<br>117/136 at 48-weeks.<br>WM:<br>109/127 at 48-weeks	Baseline data for Systolic BP, total cholesterol and HDL-C is identical for both study groups. Emailed author to query whether these measures were taken before groups were randomised as this is unclear.
44	**Dalziel 2006**	UNCLEAR	UNCLEAR	UNCLEAR	LOW	HIGH

	Assessment justification:	NS	NS	NS	"Shortly after randomisation, 21 (8 in the controls and 13 in the experimental group refused follow-up) (table 1)." The mean rate of withdrawal from follow-up was similar in the experimental (8%) and control (7%) groups.	Did not explicitly aim for weight loss so may introduce clinical heterogeneity into the review. Included after discussion as dietary intervention versus control.
45	**Damschroder 2014**	LOW	UNCLEAR	LOW	LOW	
	Assessment justification:	"…a biostatistician provided block randomized assignments (by medical center and two BMI categories [o35 or Z35] to ensure balance between groups) using random permutated blocks constructed by Stata's *ralloc* command; block sizes ranged from 3 to 9…"	"Investigators were blind to assignments until baseline assessments were complete."	Anthropometric measures (height, weight, and waist circumference); blood pressure; and self-reported measures including a Food Frequency Questionnaire; EuroQoL-5D utility assessment (with level of painsubscale); Satisfaction with Life Scale; demographic characteristics; laboratory testing for cholesterol and glucose metabolism; and a 6-minute walk test were collected in baseline, 3-month, and 12-month assessments.	Move:<br>3mth: 115/159;<br>12mth: 119/159<br>Aspire phone:<br>3mth: 131/162;<br>12mth: 120/162<br>Aspire group:<br>3mth: 127/160;<br>12mth: 122/160;<br>Follow up 332/481 consented to long term follow up.<br>Move:<br>18mth: 92/112;<br>24mth: 90/112<br>Aspire phone:<br>18mth: 95/105;<br>24mth: 92/105<br>Aspire group:<br>18mth: 102/115;<br>24mth: 104/115	
46	**Daubenmier 2016**	LOW	LOW	LOW	LOW	
	Assessment justification:	"A computer-generated random allocation sequence using random block sizes of four to eight was programmed by a database manager not involved in enrollment."	"No other staff had access to the randomization sequence. The project director (PM) accessed the allocation sequence using a programmed database that could not be altered once randomized condition was revealed."	"Weight was measured to the nearest 0.1 kg on a calibrated digital scale (Wheelchair Scale 6002, Scale-Tronix, Carol Stream IL), with participants wearing a hospital gown. The same scale was used for measurements throughout the study."	At 18-months follow-up 81% of participants from the mindfulness group and 71% from the control group were followed up.	
47	**Daumit 2013**	UNCLEAR	UNCLEAR	LOW	LOW	

	Assessment justification:	"Randomization was stratified according to sex and study site; assignments were generated in blocks of two and four." No further detail given	NS	Weight objectively measured. Measurements of blood pressure, waist circumference, and fasting blood chemical levels were obtained at baseline and at 6 and 18 months.	Control: 142/147<br>Intervention: 137/144	
48	**deVos 2016**	UNCLEAR	LOW	LOW	LOW	LOW
	Assessment justification:	"...subjects were randomized using consecutive case numbers. For the diet-and exercise program, subjects were randomized 1:1 using block randomization with block size 20."	"A research assistant not involved in the trial provided a sealed envelope that was opened by the subject in the presence of the researcher."	"For the first 2.5 y, all participants were home-visited every 6 mo by a research assistant..." "Body weight was also measured during these visits." "After 6.6 y, participants were visited once more for measurements and a questionnaire."	After 2.5 y, 10.1% of the participants were lost to follow-up. After 6.6 y, 247 participants (60.7%) agreed to additional measurements and questions. "No significant difference in attrition rate was found between the randomly assigned groups."	Original study design included 4 groups ((1) Lifestyle intervention plus placebo; (2) Lifestyle intervention plus Glucosamine; (3) Control plus placebo; (4) Control plus Glucosamine) which were combined into two groups. "The preventive effects of a weight-loss program and of oral glucosamine sulfate compared with placebo on the incidence of knee osteoarthritis were investigated in a 2x2 factorial design with a follow-up time of 6.6 y." No effects of glucosamine on these outcomes were expected or detected. Therefore, the glucosamine intervention will be disregarded in the present manuscript."
49	**Delahanty 2015**	UNCLEAR	UNCLEAR	HIGH	LOW	
	Assessment justification:	NS	NS	"Participants' height and weight were measured twice and averaged on a stadiometer (baseline only) and digital scale, respectively."<br>"To evaluate sustainability of weight loss, clinically obtained weights were abstracted from medical records, if available, 1 year after randomization date	"95% retention at 6 months." At 12 months, 2 GLI and 5 MNT participants had missing clinical data.	

				with a 10- to 14-month window."		
50	**Djuric 2002**	UNCLEAR	UNCLEAR	HIGH	LOW	HIGH
	Assessment justification:	48 patients were randomly assigned into four research groups by random block design.<br>At baseline: "There were no differences among the four groups in body weight and BMI. Nevertheless, there were significant differences in percentage body fat, total cholesterol, and LDL-C at baseline, indicating that the block randomization process did not equalize all parameters among groups. However, the highest values of these parameters were not consistently found in any one group."	NS	'Weighed in clothing but without shoes using a professional beam scale (model 402KLS; Health-o-Meter, Bridgeview, IL), and percentage of body fat was measured using tetrapolar bioelectrical impedance (model BIA101S; RJL Systems, Clinton Township, MI). Height was measured at baseline only.'	18.75% dropped out by end of study.<br><br>At 12-months:<br>Control: 12/13<br>WW: 8/11<br>Individualised: 9/13<br>Comprehensive: 10/11	Missing outcome data - study states intention to follow up to 30 months but 30-month data not available. Data for 3 and 6 months extracted from graphs but some inconsistency between graphs and what is reported in text.
51	**Duncan 2016**	LOW	LOW	LOW	HIGH	
	Assessment justification:	"…participants were randomized into one of two groups using a simple randomization procedure stratified by clinic with a 1:1 allocation ratio."<br>"…the order of control and intervention envelopes was distributed at each practice using a computer-generated randomization list."<br>"Participants were randomized within practices such that some within a practice were assigned to treatment and some to control conditions. Participating practices were not used as the unit of randomization to avoid between-practice effects confounding between-group differences."	"Practice nurses and physicians were blinded to the designation of the envelopes…" (notifying participants of allocation to either control or intervention)	Weight objectively measured.	While participant drop-out from baseline to 4 months was 49%, the lack of between-group differences in baseline demographic and health indicators in individuals that dropped out of the study indicates that systematic bias was not introduced.<br><br>"Of the 320 participants randomly assigned to control and intervention groups, 156 (48.8%) were followed-up at 4 months, with 157 (49.1%) at 12 months."	
52	**Eakin 2014**	LOW	LOW	LOW	LOW	LOW

	Assessment justification:	"Randomization was by the minimization method (18) using the MINIM program (www.sghms.ac.uk/depts/phs/guide/randser.htm)."	"Allocation is performed using the free Minim computer software [48] and conducted by a research assistant with minor involvement in participant recruitment."	"Data are collected via objective measurements conducted in participants' homes, telephone interviews, and selfadministered questionnaires at baseline, 6-, 18-, and 24- months by research staff and registered nurses blind to participants' study group."	"Attrition at 24 months was nondifferential and modest in both groups, yet ;40% of telephone counseling participants chose to discontinue receiving the intervention by withdrawal from either the intervention or study participation altogether."	"... even among telephone counseling group participants who did not withdraw, intervention delivery was difficult, with just over half of participants completing at least 75% of scheduled intervention calls."
53	**Fernandez-Ruiz 2018**	UNCLEAR	UNCLEAR	LOW	LOW	
	Assessment justification:	Randomisation was performed using a simple table of numbers: 37 patients in the control group and 37 in the experimental group.<br>"A random allocation sequence was generated by a member of the scientific staff through extraction of successive numbered balls from an opaque container, alternating between the experimental and the control group'	Refer to 'Random sequence generation (selection bias)'.	"The efficacy of the intervention was evaluated through anthropometric (body mass index, weight, different parameters, and skinfolds, as stated in Section 2) and cardiovascular measures taken before, during, and after intervention.'<br>'Anthropometric and cardiovascular measures were taken at the pretest stage, every 6 months during the programme, and 1 year after it finished."	No loss to follow up reported.	
54	**Fisher 2011**	UNCLEAR	UNCLEAR	LOW	UNCLEAR	UNCLEAR
	Assessment justification:	NS	NS	Weight objectively measured.<br><br>Total and regional body composition, including total fat mass,<br>percent body fat, leg fat mass, and lean body mass were measured<br>by dual-energy X-ray absorptiometry	NS	The weight loss programme varied in length based on when weight loss target was achieved. However, weight taken when weight loss target achieved, at approximately 6mths.<br><br>Subjects were evaluated in the overweight state (prior to any intervention). Weight was stabilized for 4 weeks through dietary control. All testing was conducted following the weight stabilization period, and in the follicular phase of the menstrual cycle. During the<br>weight stabilization period, body weights were measured three to five times per week. Fisher et al.

						Page 2<br>Obesity (Silver Spring). Author manuscript; available in PMC 2011 June 1 NIH-PA Author Manuscript NIH-PA Author Manuscript NIH-PA Author Manuscript<br>General Clinical Research Center (GCRC) at UAB. A macronutrient-controlled diet was<br>provided during the final 2 weeks of weight maintenance.
55	**Foley 2016**	LOW	UNCLEAR	LOW	LOW	
	Assessment justification:	"Randomization occurred at the baseline visit, using a computer-based algorithm. The randomization algorithm allocated participants equally (1:1) across treatment arms, after accounting for CHC, gender and ethnicity (Hispanic vs. non-Hispanic) in order to ensure the equal representation of these characteristics across arms."	NS	Weight measured objectively.	Less than 50% attrition at 12-month follow-up.	
56	**Foster-Schubert 2012**	LOW	LOW	LOW	LOW	UNCLEAR
	Assessment justification:	"The random assignment was generated by a computerized program, stratified according to BMI (<30 kg/m2 or ≥30 kg/m2) and participants' self-reported race/ethnicity (non-Hispanic white, black, or other). In addition, to achieve a proportionally smaller number of women assigned to the control group, a permuted blocks randomization with blocks of four was used, wherein the control assignment was randomly eliminated from each block with a probability of ~1 in 4."<br>Blocked-randomisation. (Permuted-block randomization (ratio 0.75 : 1 : 1 : 1) to assign a proportionally smaller number of women to the control group.)	Central computerised allocation.	Weight measured objectively.	91% followed up at 12m overall: 92% D+E, 89% D only, 91% E only, 92% usual care. 2 unavoidable losses (<1%); 8% missing; 1% medical reason.	Control group received intervention at 12m, unclear if they knew in advance.
57	**Fuller 2012**	UNCLEAR	UNCLEAR	LOW	LOW	

	Assessment justification:	The randomisation process was completed by the study database (Filemaker Pro), upon entry of the participant's initials and fulfilment of trial requirements.	NS	Weight objectively measured.	22/35 Korean group and 28/35 Western group completed the study.	
58	**Green 2015**	LOW	UNCLEAR	LOW	LOW	
	Assessment justification:	Minimisation.<br>Participants were assigned to intervention or usual-care using a stratified blocked (on gender and BMI [27–34.9 and ≥35]) randomization procedure, within sites. We used computer and paper-based randomization systems; sequence generated by author NAP.	Staff not involved in data collection informed participants about randomization. Others were blinded to assignment, and participants were routinely reminded not to discuss assignment during assessments. Usual care participants were free to pursue alternative weight-loss efforts.	Blinded staff collected data at all study periods, including scale-measured weights.	Follow-up rates were 90.5% of participants at 6 months (n = 181), 85% at 1 year (n = 170), and 81.5% (n = 164) at 2 years (83.2% if 3 deaths are removed). We found no significant differences in attrition between study arms at any assessment point.	
59	**Hageman 2017**	LOW	LOW	LOW	LOW	
	Assessment justification:	"A randomization schedule was created by the project statistician using online software to generate sequences of pseudorandom numbers (http://www.randomizer.org/form.htm). To keep accrual relatively even during rolling enrollment, a random ordering of block sizes 12, 15, and 18 was used. The project statistician did not have any contact with the women during the trial."	'At completion of the first baseline visit, nurses delivered a sealed confidential envelope to each woman that contained an identification number and a password for logging into the intervention website and advised women to keep their login information materials confidential.'<br><br>'Upon completion of baseline assessment visit two, each woman received an electronic notice on her intervention web account of her group assignment. The women were instructed to not share this with others, including the research nurses who conducted the assessments, who were blinded to web intervention content as well.'	Hageman 2011: 'The Tanita Model [TBF-215, Tanita Corporation of America, Inc., 2625 S. Clearbrook Dr., Arlington Heights, IL 60005-9824] will be used to measure height, weight and percent body fat following the manufacturer's instructions. Women will be asked to fast within 4 hours of the test, not exercise intensively within 12 hours of the test, avoid alcohol 48 hours before testing and to void the bladder within 30 minutes prior to the test, as this bioelectrical impedance analysis system measure is sensitive to hydration status .'	WO: 76/101<br>WD: 67/100<br>WE: 73/ 100	
60	**Hardcastle 2013**	LOW	UNCLEAR	LOW	LOW	

	Assessment justification:	"A statistician, who had no contact with the participants, was asked to develop a randomisation protocol such that participants were allocated to the MI intervention and minimal intervention groups by a ratio of 7:5. The randomisation protocol was stratified by gender and age based on patient records. The patients within each stratum were divided into blocks of 12 and then randomly allocated to the MI intervention and minimal intervention groups using computer generated random numbers by the predetermined ratio."	NS	"The practice nurse was blind to the treatment allocated to each patient at baseline and subsequent assessments."	At 18 months, 41% from the intervention group and 31% of the control group were lost to follow-up.	
61	**Harrigan 2016**	LOW	LOW	HIGH	HIGH	
	Assessment justification:	"Permuted-block randomization with random block size was performed by the study biostatistician"	"...blinded study staff using unmarked envelopes."	"Height (using a stadiometer) and weight were measured at baseline and 6 months." "... self-reported weight from baseline to 12 month"	Because there were 15 (15%) individuals who were missing body weight measurements at 6 months, multiple imputation with data augmentation under the multivariate normal model was conducted using SAS PROC MI, as described by Allison.15 The final results were consistent with the results without multiple imputations.<br><br>Completed 12-Months: Usual care: N = 19 (58%);<br>Telephone: N = 15 (44%);<br>In-Person: N = 22 (67%)	
62	**Hunt 2014**	LOW	LOW	LOW	LOW	HIGH
	Assessment justification:	"After baseline measurement, the randomisation<br>sequence was generated by the Tayside Clinical Trials<br>Unit (TCTU) statistician (with no day to day role in the<br>study at this point) with SAS (version 9.2), blocked (block<br>size between two and nine dependent on how many<br>participants were recruited at a club), and stratified by<br>club."	"The allocation sequence was sent in a password protected file to a database manager (not part of the<br>research team) who assigned individuals to each group."	Weight measured objectively.	Comparison (control): 347/374 at 12-weeks; 355/374 at 12-months.<br>Intervention:<br>330/374 at 12-weeks; 333/374 at 12-months	12 month wait-list control.

63	**Irwin 2003**	LOW	LOW	LOW	LOW	
	Assessment justification:	“Randomization was performed by random number generation…”<br>“Randomization was stratified by BMI (<27.5 vs >27.5) to ensure equal numbers of heavier and lighter women in each study group.”	“…group assignment was placed in a sealed envelope…”	Weight measured objectively.	Less than 50% attrition at 12-months follow-up.	
64	**Jebb 2011**	LOW	LOW	LOW	LOW	
		"The randomisation sequence was computer generated with Stata (version 9.0) by APM and built into the database by the data manager, who was independent from the study team, and was stratified by country, sex, and diabetes status, with an upper limit of 50% of participants with diabetes."	"Treatment allocation was concealed by use of an online database (Filemaker Pro 9, version 3)."	"In the UK and Australia, bodyweight (in light clothes without shoes) and fat mass were measured with a Tanita BC-418 segmental body composition analyser (Tanita Corporation of America, Arlington Heights, IL, USA). In Germany, weight was measured in GP practices with standard scales, and fat mass was measured at the research centre with the Tanita BC-418."<br><br>"All participants who did not complete the 24-month visit but had not formally withdrawn from the study were asked to provide self-reported weights in a telephone follow-up survey (Australia and the UK) or a postal survey (Germany)." Majority was objectively reported.	12-month retention rate:<br>Standard care: 214/395*100 = 54.2%<br>Commercial programme: 230/377*100 = 61%<br><br>18-month retention rate:<br>Standard care: 115/395*100 = 29.1%<br>Commercial programme: 121/377*100 =32.1%<br><br>24-month retention rate:<br>Standard care: 98/395*100 = 24.8%<br>Commercial programme: 105/377*100 = 27.9%	
65	**Jebb 2017**	LOW	LOW	LOW	LOW	
	Assessment justification:	"An independent statistician produced a computer generated randomisation list with 1:1 allocation using stratified block randomisation"	"After the nurse had confirmed eligibility, participants were enrolled in the study and the allocation was revealed using an online randomisation programme to ensure full allocation concealment. "	Weight was objectively measured.	95/140 UC and 104/138 TDR followed up at 12 months.	

66	**Jenkins 2017**	LOW	LOW	LOW	HIGH	
	Assessment justification:	"A statistician not involved in the day-to-day operation of the interventions created blocks of random assignments (n=39)."	"Assignments were sealed in ordered, numbered, opaque envelopes. Upon consent and eligibility confirmation for the individual or household, the coordinator opened each envelope in sequence and assigned the participant to the treatment group it contained."	"Completed questionnaires, fasting blood, anthropometric, and blood pressure measurements were obtained at baseline and at subsequent clinic attendances at 6 and 18 months at St. Michael's Hospital."	6-month retention of 91% in the 2 food delivery arms versus 67% when no food was provided. When no food was provided, groups which had received a prior provision of food resulted at 18 months in an 81% retention versus 57% where no food had been provided.	
67	**Katula 2013**	LOW	LOW	LOW	LOW	
	Assessment justification:	"Eligible participants were randomly assigned, if equal probability, to either the lifestyle intervention or the enhanced usual care arm using a web-based data management system that verifies eligibility."	Refer to 'Random sequence generation (selection bias)'.	"Assessments are performed at 6 month intervals (baseline, 6-, 12-, 18- and 24-months post-randomization) at the GCRC. Psychosocial measures are self-administered and remaining measures are completed by trained study staff or clinic staff."	6-month assessment visit: UC: 141 attended (94%); LWL: 139 attended (92%);<br>12-month assessment visit: UC: 138 attended (92%); LWL: 135 attended (89%);<br>18-month assessment visit: UC: 132 attended (88%); LWL: 125 attended (83%);<br>24-month assessment visit: UC: 134 attended (89%)L LWL: 127 attended (84%)	
68	**Katzer 2008**	UNCLEAR	UNCLEAR	LOW	LOW	
	Assessment justification:	"Randomization was stratified by age and BMI and performed independently by a statistician."	NS	Weight objectively measured.	"By the end of the initial 10-week intervention, 53 participants had withdrawn from the study (24%), and an additional 28 participants (overall dropout rate=37%) had withdrawn by the 12-month follow-up. Dropout rates were similar in the three treatments (data not shown)."	
69	**King 1989**	LOW	LOW	LOW	LOW	HIGH

	Assessment justification:	The subjects were randomly assigned within each cohort by selecting one envelope for a set of sealed envelopes.	Sealed envelopes.	"Subjects' height and weight were measured using a balance-beam scale, with subjects wearing normal indoor clothing (without shoes). Subjects were weighed at the start and at months 6 and 12 of year 2 by staff members blind to each subject's year-2 condition assignment."	"Of the 51 subjects initially randomized to weight loss through energy restriction during year 1, 44 (86.3%) participated in the year-2 maintenance study. Of the 52 subjects initially randomized to weight loss through exercise during year 1,46 (88.5%) participated in the year-2 maintenance study."<br><br>"Of the 90 subjects participating in the maintenance study, complete year-2 total body weight data were obtained for 36 (81.8%) of 44 dieters and 36 (78.3%) of 46 exercisers."	The study randomised to three groups 1. Diet, 2. exercise or 3. control. At the end of the 1-year intervention, participants in the diet group and exercise group were re-randomised within each condition to either a maintenance condition or control condition, forming four groups for the follow-up period (the original control group was not followed up after intervention end.) Data reported was broken down into these 4 groups (formed following re-randomisation after intervention end), however we have extracted and combined data for the original 2 groups (the control group data was not extracted as this group was not followed up after intervention end).
70	**Knauper 2018**	LOW	UNCLEAR	LOW	HIGH	
	Assessment justification:	"To randomize participants to the two intervention arms, a randomization sheet generated by a random digit generator is used (www.randomizer.org)."	"Throughout the recruitment process, the list of randomized numbers will be assigned to participants by the research coordinator in sequential order from 1 to 154 in the order in which participants completes the baseline CHIP appointment."	"However, the staff assessing the outcome variables (e.g. weight, EST) is blind to which intervention the participants were assigned."	44.9% of the Enhanced DPP group attended the 12-month follow-up versus 70% of the standard DPP arm.<br><br>24-Month follow-up: Enhanced DPP: 51/107*100 = 47.7%; Standard DPP: 51/101*100 = 50.5%	
71	**Diabetes Prevention Program R G 2009**	LOW	LOW	LOW	LOW	HIGH

	Assessment justification:	"The randomization was done centrally by computer…"<br>Random treatment assignments were stratified according to clinical center and were generated by the coordinating center through computer linkup to the field center at<br>time of randomization. Therefore, assignment was unknown until randomization. Assignments to metformin and placebo were double-blinded.	"…assignments to the lifestyle group were blinded until randomization, while assignments to the medication groups were blinded until the end of the study."	Lifestyle intervention participants were weighed privately at the start of every individual session and were encouraged to weigh themselves at home daily or<br>a minimum of once per week.	Placebo<br>yr. 1 - 1027/1082;<br>yr. 2 - 1015/1082;<br>yr. 3 - 975/1082.<br>Bridge period DPPOS - 1085 eligible, 935 enrolled. DPPOS<br>yr. 1 882/935;<br>yr. 2 874/935;<br>yr. 3 844/935;<br>yr. 4 - 827/935;<br>yr. 5 - 846/935;<br>yr. 6 808/935;<br>yr. 7 - 789/935;<br>yr. 8 766/935;<br>yr 9. 760/935;<br>yr. 10 - 763/935;<br>yr. 11- 769/935.<br>Lifestyle<br>yr. 1 - 1026/1079;<br>yr. 2 - 1001/1079;<br>yr. 3 - 972/1079.<br>Bridge period DPPOS - 1068 eligible, 914 enrolled. DPPOS<br>yr. 1 855/914;<br>yr. 2 827/914;<br>yr. 3 816/914;<br>yr. 4 - 810/914;<br>yr. 5 - 824/914;<br>yr. 6 783/914;<br>yr. 7 - 763/914;<br>yr. 8 757/914;<br>yr. 9 738/914;<br>yr. 10 - 725/914;<br>yr. 11.- 738/914.	0-3 years LOW<br>From year 4 HIGH<br>DPP was a 3-year randomized clinical trial followed by open-label modified intervention follow-up.
72	**Kuller 2012**	LOW	LOW	LOW	LOW	
	Assessment justification:	Block randomisation.<br>Randomization sequence designed by independent statistician'	"…allocation via sealed, numbered envelopes opened sequentially."	Weight, height, and waist circumference were measured at clinic visits at baseline, 18 months, and 30 months. Standard laboratory measurements included total cholesterol, HDL-C, triglycerides, insulin, and glucose after 12-hour fasting samples.	83% followed up at 18m overall: 82% intervention, 84% control. Reasons for attrition NS.	
73	**Kumanyika 2012**	LOW	LOW	LOW	LOW	

	Assessment justification:	“Eligible participants stratified by gender and age (~35 or over 35 year) were randomized to one of two treatment groups in a 1:1 ratio with randomly permuted blocks (block sizes of 2-6).”	“Random assignments were concealed from both participant and study staff prior to implementation.”	Weight objectively measured.	Descriptive analysis:<br>Basic Program: 98/137 at 12mths<br>Basic Plus Program: 89/124 at 12mths<br>Weight change (>= 1 wt measurement after baseline)<br>Basic program 133/137 at 12mths<br>Basic plus program 124/124 at 12mths	
74	**Ley 2004**	HIGH	UNCLEAR	LOW	LOW	
	Assessment justification:	“They were then individually randomised to either an intervention group that was asked to consume a reduced-fat (RF), but otherwise ad libitum diet, or a control diet (CD) group that continued with their usual diet. An exception to this individual randomisation was made at one work-site where all six participants were Pacific Island’s women who worked closely together. They were all assigned to the RF group because individual randomisation was impractical. All those found to have diabetes on re-testing were referred to their general practitioners for management, but were still randomised for the study.”<br><br>Broken randomisation.	All those found to have diabetes on re-testing were referred to their general practitioners for management but were still randomised for the study.	Weight objectively measured.	less than 50% attrition at 1 - and 5-year follow-up.	
75	**Li 2016**	LOW	UNCLEAR	LOW	LOW	UNCLEAR

	Assessment justification:	"After a one-week run-in period, participants were randomly allocated to one of the following four groups by computer-generated random numbers."	NS	"All measurements were conducted with standard procedures by the same clinical staff in the third hospital of Inner Mongolia medical college, who were blinded to the group allocation."	Percentage of participants retained at follow-up at 1 year: Usual care: 98.3%; Diet: 96.2%; 50g oats: 96.3%; 94.9%; "Eleven patients dropped out during the 1-year follow-up due to personal reasons with no difference in drop-out rates among the four groups (p = 0.774)."	Unclear whether groups were stratified by BMI in parent study. "A subgroup of 298 subjects, meeting the Chinese criteria of overweight (body mass index ≥ 24 kg/m2), was selected from 445 adult patients with T2DM, who had participated in the 30-day centralized management of a dietary program and the 1-year free-living follow-up in Baotou, China."
76	**Li 2005**	LOW	UNCLEAR	LOW	LOW	HIGH
	Assessment justification:	"A random, permuted, block design was utilized for placement of subjects into the two treatment groups."	NS	Weight measured objectively.	Retention rate:<br>Baseline:<br>MR: 49/52*100 = 94.2%;<br>IDP: 44/52*100 = 84.6%<br><br>6-months:<br>MR: 46/52*100 = 88.5%;<br>IDP: 36/52*100 = 69.2%<br><br>12-months: MR: 42/52*100 = 80.8%; IDP: 35/52*100 = 67.2%	MR group continued to received the meal replacements for the 12 month study duration at lower volume.<br>"For the first 5 days of the study, subjects randomized into the MR group replaced three meals per day with a soy MR (Slim Fast Food Company, Inc. West Palm Beach, FL 33401, USA). They also were instructed to add fruits and vegetables to their dietary intake. Thereafter, the MR group replaced two meals with the soy MR with continuing use of fruits and vegetables as snacks, plus a sensible third meal for three additional months. After the 3 months, subjects in the MR group were instructed to replace one to two meals per day with the soy shakes and consume correspondingly one to two sensible meals for the duration of the study."
77	**Lindstrom 2003**	LOW	UNCLEAR	LOW	LOW	HIGH

	Assessment justification:	A randomization list was used	The nurses scheduling visits were blinded to randomisation. Study staff were not blinded.	Weight objectively measured.	At 3 years:<br>203/ 257 Control<br>231/ 265 Intervention<br><br>At 4 years:<br>170/257 Control<br>198/265 Intervention<br><br>From 5 years:<br>166/257 Control<br>200/265 Intervention	After the decision to end the intervention period, the intervention was continued until each participant's next scheduled annual clinic visit. The end date thus varied from March 2000 to December 2001. After active intervention (median 4 years, range 1–6 years), participants still free of diabetes and willing to continue their participation (from year 6 - 200 in the intervention group and 166 in the control group) were further followed until diabetes diagnosis, dropout or the end of 2009, with a median total follow-up of 9 years and a time span of 13 years from baseline.
78	**Liss 2016**	LOW	LOW	LOW	LOW	
	Assessment justification:	"Prior to the study enrollment phase, randomization lists were generated by a senior statistician using SAS, version 9.2 (Cary, NC). Lists were created using 1-to-1 allocation, with blocks of 4, stratified by Y study site and race (non-Hispanic White; African-American; Other)."	"Randomization blocks were implemented by the study programmer (AC) and pre-loaded into a back-end field of a Microsoft Access (Redmond, WA) database table that was not available to study RAs. After the study RA collected data required for randomization at each participant's screening/enrollment visit, she clicked a button in Access to execute the randomization."	Weight objectively measured.	At 12 months, 78% of participants from the GLI group and 76% from the standard care group returned for outcome assessment.	
79	**Little 2016**	LOW	LOW	UNCLEAR	LOW	

	Assessment justification:	"Upon completion of baseline questionnaires, the website automatically randomly assigned patients (1:1:1) via computer-generated random numbers…"	"Participants and investigators were masked to group allocation at the point of randomisaton…"	"Weight loss was measured with participants lightly clothed without shoes, at the same time every day when possible, with automated digital scales (Tanita Europe BV, Amsterdam, the Netherlands)." "When a blinded weight measurement could not be obtained, we used practice nurses' recorded weights, and when that was not possible, we used participants' reported weights."	Weight loss averaged over 12 months was recorded in 666 (81%) participants." Control: 136/279 weight at 6mths (HIGH); 227/279 weight at 12mths (LOW). Power + face to face: 148/269 wt at 6mths (LOW); 221/269 wt at 12mths (LOW). Power + remote: 155/270 wt at 6mths (LOW); 218/270 wt at 12mths (LOW).	
80	**Manning 1994**	UNCLEAR	UNCLEAR	LOW	LOW	
	Assessment justification:	NS	NS	"Weight measurements were available irrespective of whether the patient completed the defined study group, scale weights were comparable throughout."	Less than 50% attrition at 12- and 48-month follow-up.	
81	**Mefferd 2007**	UNCLEAR	UNCLEAR	LOW	LOW	UNCLEAR
	Assessment justification:	NS	NS	"Anthropometric measurements (obtained at baseline and 16 weeks) included height and weight (measured without shoes)..."	A little over ten percent of the participants dropped out of the study during the 16 weeks under analysis in this report, yielding a final sample size of (n = 76) at 16 weeks. All nine dropouts had been assigned to the intervention group.<br><br>Retention rate:<br>16-weeks:<br>Control: 100%; Intervention: 47/56*100= 83.9%<br>12-Months:<br>Control: 25/29*100 = 86%; Intervention: 44/56*100 = 78.6%	Wait-list control; unclear if control group participants were aware they were wait-listed.
82	**Melchart 2017**	LOW	LOW	LOW	LOW	

	Assessment justification:	"Randomization and allocation envelopes were prepared by an independent statistician at the Institute for Medical Statistics and Epidemiology at the Technical University of Munich."	"The trial physicians were instructed to open the sealed envelopes in a strictly sequential order of enrollment and to disclose the allocated treatment arm to the study participant."	"Body weight, height, waist circumference, blood pressure, and heart rates were measured by certified IHM coaches at each of the five examination visits. The teams were trained to perform the examinations in a standardized way (eg, subjects wearing light clothes and no shoes, with use of calibrated scales for measuring weight)."	"... Of 111 subjects who commenced with the IHM group, 17 (15.3%) prematurely discontinued the study, while in the UC group the dropout rate was 18.2% (10 of 55). The majority of dropouts left the study before the control visit at month 3, and this occurred in 10 of 17 cases in the IHM group and in 9 of 10 in the UC group."<br><br>Participant dropouts:<br>3-Months:<br>IHM: 90.8%; UC: 84%;<br>6-Months:<br>IHM: 88.1%; UC: 82%;<br>9-Months:<br>IHM: 85.3%; UC: 82%<br>12-Months:<br>IHM: 84.4%; UC: 84%	
83	**Melin 2003**	UNCLEAR	UNCLEAR	LOW	LOW	
	Assessment justification:	“The subjects were randomised into two groups according to gender, age and BMI: an intensively treated group (group 1) and a less intensively treated group (group 2.)”<br><br>No further information given.	NS	Weight objectively measured.<br>The laboratory tests were performed according to clinical routine. Blood glucose concentrations were determined by the glucose oxidase method.12 Serum insulin assays were performed by the Phadebas test (Pharmacia, Uppsala, Sweden).12 The blood pressure was measured in the right arm with a sphygmomanometer. The cuff size was 15.45 cm depending on the arm circumference. The recordings were made to the nearest 2 mmHg twice after 10 min supine rest, and the mean of the two measurements was used in the analyses.	Less intensive group: 19/21 at 6mths;<br>18/21 at 12mths;<br>15/21 at 24mths.<br><br>More Intensive group: 19/22 at 6mths;<br>17/22 at 12mths;<br>17/22 at 24mths.	
84	**Menard 2005**	LOW	UNCLEAR	LOW	LOW	UNCLEAR

		"Using a blocked randomization (n = 4) stratified by hemoglobin A1c value (< 10% and ≥ 10%), patients were assigned by an independent person using a computer program to receive intensive multitherapy or usual care."	NS	"Fasting plasma glucose levels, hemoglobin A1c concentrations, blood pressure and serum lipoprotein levels were measured after a 12-hour, overnight fast at baseline and at 6, 12 and 18 months. Weight and height were measured, and body mass index (kg/m2) was calculated. Fasting plasma glucose levels were measured using a glucose oxidaze method. Cholesterol, high-density lipoprotein cholesterol, and triglyceride levels were measured using a colorimetric process (Johnson & Johnson Ortho-Clinical Diagnostics, Rochester, NY). Low-density lipoprotein cholesterol levels were calculated with the Friedewald formula. Hemoglobin A1c concentrations were measured by high-performance liquid chromatography (Bio-Rad VARIANT, Hercules, Calif.)."	Control:<br>35/36 at 12mths;<br>29/36 at 18mths.<br><br>intervention:<br>34/36 at 12mths;<br>32/36 at 18mths.	For ethical reasons, patients in the control group had protocol-driven laboratory tests, and they and their physicians received information about diabetes and its management as well as the results of these tests. Thus, control group patients may have received more aggressive treatment and attention than they normally would have.
85	**Mengham 1999**	UNCLEAR	UNCLEAR	UNCLEAR	LOW	
	Assessment justification:	NS	NS	NS	74/75 LOW<br>'Seventy-five patients were recruited and randomised. '<br>'Of the 74 patients who completed the study'	
86	**Mensinger 2016**	LOW	LOW	LOW	HIGH	
	Assessment justification:	"This study was a 1:1 parallel-group randomized design comparing the effectiveness of two 6-month group-based "healthy living programs" (weight-neutral or weight-loss).<br>Folded index cards containing program assignments from a computer-generated randomization scheme were placed into sealed and sequentially numbered opaque envelopes."	"Folded index cards containing program assignments from a computer-generated randomization scheme were placed into sealed and sequentially numbered opaque envelopes. Upon completion of the baseline assessments where informed consent was obtained, participants were given an envelope containing a welcome letter with their assignment and	Weight measured objectively.<br>Waist and hip circumference was measured to the nearest quarter inch with a flexible tape measure on bare skin.<br>Venous blood samples were drawn after an overnight fast in order to obtain glucose levels and lipid panels (total cholesterol, LDL-C, HDL-C, total cholesterol-HDL ratio, and triglycerides). We followed	Weight neutral -<br>39/40 at 6mths (LOW); 19/40 at 24mths (HIGH)<br>weight loss -<br>33/40 at 6mths (LOW); 21/40 at 6mths (LOW).<br><br>Attrition rate at 6-month for the weight loss group > 25%, change from low to high.	

			instructions.”	standardized methods established by the National High Blood Pressure Education Program and averaged two blood pressure (BP) readings using a Welch Allyn cuff with an aneroid sphygmomanometer.		
87	**Mitsui 2008**	UNCLEAR	UNCLEAR	UNCLEAR	LOW	
	Assessment justification:	"...were randomly assigned to the intervention group..."	NS	NS	"Two participants in the intervention group and 1 in the control group dropped out of the program after week 12 for personal reasons."	
88	**Moreno 2014**	UNCLEAR	UNCLEAR	LOW	LOW	
	Assessment justification:	"Patients were randomised and allocated to receive ... "	NS	Weight measured objectively.<br>WC was recorded with a standard flexible nonelastic metric tape over the midpoint between the last rib and the iliac crest, with the patient standing and exhaling.	At 12-months:<br>LC: 26/40<br>VLCK: 27/39<br>At 24-months:<br>LC: 23/40<br>VLCK: 22/39	
89	**Morgan 2010**	LOW	LOW	LOW	LOW	
	Assessment justification:	"The random allocation sequence was generated by a computer-based random number-producing algorithm in block lengths of six to ensure an equal chance of allocation to each group."	"To ensure concealment, the sequence was generated by a statistician and given to the project manager. Randomization was completed by a research assistant who was not involved in the assessment of participants and the allocation sequence was concealed when enrolling participants."	"Weight was measured without shoes on a digital scale to 0.1kg (model CH-150 kp; A&D Mercury, Adelaide, Australia)"<br><br>Systolic and diastolic blood pressure and resting heart rate were measured using a NISSEI/DS-105E digital electronic blood pressure monitor (Nihon Seimitsu Sokki, Gunma, Japan) under standardized procedures.	Retention rate:<br>3-month follow-up: Control: 27/31*100 = 87.1%;<br>SHED-IT: 28/34*100 = 82.4%<br><br>6-month follow-up: Control: 26/31*100 = 83.9%;<br>SHED-IT: 28/34*100 = 82.4%<br><br>12-month follow-up: Control: 20/31*100 = 64.5%;<br>SHED-IT: 26/34*100 = 76.5%	
90	**Muggia 2014**	LOW	LOW	UNCLEAR	HIGH	

	Assessment justification:	“Participants were randomly allocated (allocation ratio 1:1) to the standard care or cognitive behavioral therapy group, using a computer-generated randomization application of STATA statistical package.”	“Randomization list was kept at Biometric Unit and clinicians were unaware of the treatment group until the subjects were enrolled. The treatment allocation was communicated by phone to the clinician every time a new patient was enrolled.”	Information on how weight was measured not stated.	A high attrition rate is observed and although multiple imputations are performed to reduce its impact bias, this cannot be excluded as also reported in similar studies.<br>At the six month, 114 patients (69.9% of the total) attended the follow-up visit, with an attrition rate of 30.1%. The percentage of visits attended was significantly greater in group A (83.3% vs 70.4% in group B, p < 0.001). At the 12 month, 78 patients (47.8% of the total) attended the follow-up visit, with an attrition rate at 1 year of 47.8 per 100 person-year. A total of 44 patients in the treatment arm A (53.0%) and 34 (42.5%) in group B completed the follow-up, with no significant differences between the two groups.	
91	**Nakata 2014**	LOW	LOW	LOW	LOW	UNCLEAR
	Assessment justification:	" After the motivational lecture, the participants were randomly assigned to one of the 3 groups using simple randomisation procedures involving computerised random numbers."	"The allocation data were generated by an investigator (MO) who had no contact with the participants or other staff members, and the data were maintained at a central secure location until completion of the motivational lecture."	"Data were collected at baseline and at months 3, 6, 18 and 30 in the hospital by trained hospital staff members who were blinded to the treatment assignment process. The primary outcome measure was the amount of weight lost from baseline to 30 months."	The attrition rates were 9.6% (12/125) and 20.0% (25/125) at months 18 and 30, respectively (Fig. 1). The numbers of individuals lost to follow-up at 30 months were similar in both groups (p = 0.531).	"Due to ethical concerns, we provided group-based support to the control group after the 6-month intervention period and did not include them in the follow-up measurements."
92	**Nanchahal 2012**	LOW	UNCLEAR	LOW	LOW	

	Assessment justification:	"Participants were randomly allocated (allocation ratio 1:1) to the control or intervention group (TP, EH, AS), using a computer-generated randomisation application written in VBA for MS Access (TP). The Taves method of minimisation 48 was used to ensure the groups were balanced for general practice, gender, age group (≤50/>50 years), BMI category (≤30/>30 kg/m2), diagnosis of diabetes (yes/no) and taking antipsychotic medication or not."	NS	The study was single blinded with members of the study team assessing baseline and follow-up measurements blinded to group assignment.<br><br>Weight (in light clothing) was measured using the Tanita (BC 420 MA) scales. The scales also reported per cent body fat, basal metabolic rate and metabolic age (age expected for a given value of basal metabolic rate). Waist was measured midway between the iliac crest and the costal margin to the nearest 0.1 cm. Blood pressure and heart rate were measured using a digital automatic monitor (Omron Model M10-IT), with the average of three readings recorded where possible.	Retention rate:<br>6-months<br>(step change in intervention intensity): Control: 67.9%; CAMWEL: 70.2%<br><br>12-months:<br>Control: 60.0%; CAMWEL: 53.9%	
93	**Ng 2015**	LOW	UNCLEAR	LOW	LOW	
	Assessment justification:	“Recruited patients were randomized in 1:1 ratio to participate in the LMP or usual care through the use of a computer-generated list of random numbers.”	NS	"Anthropometric measurements, ESS, and laboratory tests, which included liver and renal function, fasting glucose, and lipids, were performed at baseline, 4 months, and 12 months."	"Sixteen participants in the intervention group were excluded after randomization, as six had never attended dietician visits, seven attended fewer than four dietician visits, and three maintained their high-energy and -fat food intake. There were six subjects in the control group lost to follow-up."<br><br>LMP Group:<br>45/61*100 = 73.8%;<br>Control:<br>37/43*100 = 86%	
94	**Nilsen 2011**	LOW	LOW	LOW	LOW	
	Assessment justification:	Groups were randomly assigned to an “individual physician group” (IG) or an “individual plus interdisciplinary group” (IIG) by use of closed envelope method with unknown block sizes.	Closed envelope method.	“At every visit to the study physician, the following assessments were performed: fasting blood sample, systolic and diastolic blood pressure (SBP and DBP) according to	Control IG: 89/104 at follow up<br>Intervention IIG 93/109	

				recommended standards [18], waist circumference at a level midway between the lowest rib and the iliac crest to the nearest cm, height without shoes to the nearest cm (only first visit) and weight in indoor clothes to the nearest 100 g. Blood pressures were measured by an Omron M41 and weight with a Seca 771."		
95	**Nordby 2012**	LOW	LOW	UNCLEAR	HIGH	
	Assessment justification:	"The randomization was performed over the three 6-mo blocks, using a manual lottery in which participants drew their own lot."	"Sixty participants were randomized, each participant drawing his own lot."	"Before, during, and after the intervention, participants underwent a panel of tests."	Dropout rates for each group post intervention were >20% for all groups. "Of the 36 participants who completed T, D or T-iD, 28 participated in follow-up visits: 9 participants in T (6 months: n = 7; 12 months: n = 8), 10 participants in D and 9 participants in T-iD)." Only 41% of participants in group T participated at the 6-month time point.	
96	**Oldroyd 2006**	LOW	UNCLEAR	LOW	LOW	
	Assessment justification:	Eligible participants who agreed to take part were randomly allocated using a random number table to the intervention or control group at the first baseline appointment.	"Researchers performing the randomisation were blind to the group allocation."	Weight was measured to the nearest 0.1 kg with the participants lightly clothed on SECA scales (Alpha Model 770 digital, SECA Limited, Birmingham, UK)	6-month follow-up: Control: 32/39 * 100 = 82%; Intervention: 37/39*100 = 94.9%<br><br>12-month follow-up: Control: 30/39 * 100 = 77%; Intervention: 32/39*100 = 82%<br><br>24-month follow-up: Control: 24/39 * 100 = 62%; Intervention: 30/39*100 = 77%	
97	**Pan 1997**	UNCLEAR	UNCLEAR	LOW	UNCLEAR	HIGH
	Assessment justification:	NS	NS	"Briefly, blood pressure, height, and weight were measured in light clothing without shoes following methods used in the WHO multinational study of vascular disease in diabetes	NS	"The original 6-year study randomised to 4 groups (1. control, 2. diet, 3. exercise 4. diet and exercise). However, for the 20-, 23- and 30 year follow-ups, the authors combined the

				(17)."		intervention groups and only reported data for the 'control' versus 'intervention' arms. Authors state that the reason for this was that: "As diabetes incidence did not differ significantly among the three intervention groups during the active intervention period and because of limited power to detect differences, the intervention groups were combined and comparisons were made between the combined intervention group and the control group."
98	**Parikh 2010**	UNCLEAR	UNCLEAR	LOW	LOW	
	Assessment justification:	"Participants were randomized to intervention or delayed intervention (in 1 year) by blocked randomization (block size=4) by recruitment site."<br><br>No further information given.	NS	Weight measured objectively.	"The study had some attrition: 83 participants returned at 3 months, 79 at 6 months, and 72 at 12 months (37 control, 35 intervention). Four participants became ineligible because of pregnancy. The 23 participants lost to follow-up at 12 months did not differ from those who returned for the final check-up in age, gender, weight, BMI, or family history of diabetes.”	
99	**Pedersen 2013**	LOW	UNCLEAR	LOW	LOW	UNCLEAR

	Assessment justification:	"Randomization was stratified according to BMI (≤ 32.5; > 32.5). A third party unrelated to the study performed en bloc randomization with bloc size 2, 4 and 6 using Stata 11.1 software (StataCorp, 4905 Lakeway Drive, College Station, TX, USA)."	NS	"All participants are examined at baseline, after 12 weeks and after a year. Most examinations were performed at University Hospital of Bispebjerg, Department of Cardiology, except the MRI that was performed at University Hospital of Herlev and PET that was performed at Rigshospitalet." This included body composition assessed by anthropometry.	Dropout rate at 12-weeks follow-up:<br>AIT: 31/35*100 = 88.6%;<br>LED: 34/35*100 = 97.1%<br><br>Drop out rate at 1-year follow-up:<br>AIT: 26/35*100 = 74.3%;<br>LED: 29/35*100 = 82.9%	"Drop-out rates (26% and 17% in the AIT and LED+AIT group, respectively) imply that intensive lifestyle changes require physical and mental strength and support from relatives and employers especially when considering long-term interventions." "However, drop-out rates introduce a risk of bias due to small sample size and challenges related to generalisability as discussed above. "
100	**Pettman 2009**	LOW	LOW	LOW	LOW	
	Assessment justification:	"Using a random number generator (MS Excel), participant data were then distributed into three groups of approximately equal numbers. Unidentifiable individuals were block-matched to achieve an even gender balance and distribution of MetS risk factors over the 3 groups by calculating means for waist, DBP and age together with counts of males and females for each group. The groups were checked for significant differences between variables using independent samples t-tests. The three groups were then randomly assigned to 'A', 'B' or 'C' corresponding to INT-A, INT-B or CON respectively."	"Study personnel generating the sequence were not aware of participant details, due to obscuring of identification numbers. Final group assignment was conducted by an impartial person."	"Body weight was measured to the nearest 0.1 kg (Tanita Ultimate scales™ Model 2000, Tanita Corporation, Tokyo, Japan), except for individuals weighing over 150 kg, who were weighed on a single set of electronic glass scales (Model 3200, Propert Pty Ltd, Castle Hill,<br>NSW, Australia). The same set of scales was used at subsequent measurements for each participant."	Retention rate:<br>4-months:<br>Control: 86%;<br>INT-A: 98%;<br>INT-B: 92.6%<br><br>12-months:<br>Control: 36/43*100 83.7%;<br>INT-A: 44/48*100 = 91.6%;<br>INT-B: 35/49*100 = 92.6%	
101	**Promrat 2010**	LOW	LOW	LOW	LOW	
	Assessment justification:	"Randomization was performed using a random number generator developed by the project statistician, with a target enrollment of 30 participants."	"The randomization process was conducted by a project staff who was blinded to the randomization sequence."	"Data collection was obtained by trained staff who were not aware of the group assignment or sequence of measurement."	"Thirty participants (97%) completed the study. One participant (3%) in the lifestyle intervention group withdrew from the study after 3 months. All other participants adhered to the study protocol follow-up schedule."	

102	**Provencher 2009**	UNCLEAR	UNCLEAR	LOW	LOW	
	Assessment justification:	"Randomization was performed within each phase, and women were then assigned to one of the 3 treatment conditions: HAES group (N = 48), SS group (N = 48), or control group (N = 48).	NS	"Height was measured to the nearest millimeter with a stadiometer, and body weight was measured to the nearest 0.1 kg on a calibrated balance. Participants were asked to dress lightly and to remove their shoes for these measurements."	Baseline (T=0):<br>Control: 46/48*100 = 95.8%;<br>SS Group: 46/48*100 = 95.8%;<br>HAES: 100%<br><br>4-months (T=4): Control: 38/48*100 = 79.2%;<br>SS Group: 39/48*100 = 81.3%;<br>HAES: 44/48*100 =91.7%<br><br>10-months (T=10): Control: 34/48*100 = 70.8%;<br>SS Group: 38/48*100 = 79.2%;<br>HAES: 45/48*100 =93.8%<br><br>16-months (T=16): Control: 32/48*100 = 66.7%;<br>SS Group: 33/48*100 = 78.8%;<br>HAES: 41/48*100 =85.4%	
103	**Ridgeway 1999**	UNCLEAR	UNCLEAR	LOW	LOW	
	Assessment justification:	NS	NS	"The patients were weighed..."	CG: 20/28 = 71%; IG: 18/28 = 64%	
104	**Rock 2015**	LOW	LOW	LOW	LOW	
	Assessment justification:	"Random assignment was performed by a centralized computer process, assigning participants in a 1:1 ratio to either the intervention arm or the less intensive intervention control arm, stratified by age (or 55 years), stage (I v others [II and III]), and study site.	"Randomization was performed by a centralized computer process"	Weight was measured at baseline and at 6-, 12-, 18- and 24-month follow-up visits, using a calibrated scale.	"Weight was not available for 44 intervention group and 61 control group participants at 24 months"	
105	**Rolls 2005**	UNCLEAR	UNCLEAR	LOW	LOW	
	Assessment justification:	"…a stratified randomization scheme was used to balance the distribution of subject sex and age across the groups."	NS	"Body weight was measured at each counseling session, with the subject wearing light clothing without shoes, using a scale that was regularly calibrated."	Less than 50% attrition at 12-month follow-up.	
106	**Rolls 2017**	LOW	UNCLEAR	LOW	LOW	

	Assessment justification:	"Participants were stratified by body mass index and age, and randomly assigned to one of three groups, using blocks of six sequences from a random number generator."	NS	"Subjects were weighed to the nearest 0.1 kg while wearing a lightweight outfit kept for them at the center. Height was measured with a stadiometer; waist circumference was measured at the right iliac crest.	3-month:<br>total sample follow-up: 170/ 186* = 91.4%; Standard advice group: 59/62*100 = 95%; Portion selection: 58/62*100 = 93.5%; Pre-portioned foods: 59/62*100 = 95%<br><br>6-month:<br>total sample follow-up: 149/ 186* = 80.1%; Standard advice group: 52/62*100 = 83.9%; Portion selection: 51/62*100 = 82.3%; Pre-portioned foods: 53/62*100 = 85.5%<br><br>12-month:<br>total sample follow-up: 136/ 186* = 73%; Standard advice group: 49/62*100 = 79%; Portion selection: 51/62*100 = 82.3%; Pre-portioned foods: 51/62*100 = 82.3%	
107	**Rosas 2015**	LOW	UNCLEAR	LOW	LOW	
	Assessment justification:	Participants are randomized to one of three arms according to the ratio 1 UC: 2 CM: 2 CM+CHW. After all baseline data were collected, a blinded data analyst/biostatistician confirmed study data completion and randomizes the participant to one of the three arms in permuted blocks stratified by sex, BMI (30-34.9, 35-39.9, or ± 40), and diabetes status.	The data analyst/ biostatistician was blinded.	Data collection staff were blinded to treatment assignment.<br><br>Weight was measured at each assessment visit in duplicate using a Detecto scale, whereas height was measured in duplicate using a wall-mounted stadiometer at baseline only. Participants' anthropometric measures were assessed without their shoes and coats.	As in other lifestyle intervention trials, all participants did not attend all planned intervention activities (one-on-one case management, groups sessions, and home visits). This limited our ability to test whether the planned intervention had the intended effect. Nevertheless, the percentage of participants attending each activity was within the expected range. Body weight was collected from 207 participants (100%) at baseline, followed by 190 (91.8%) at 6 months, 171 (82.6%) at 12 months, and 177 (85.5%) at 24 months.	
108	**Ross 2012**	LOW	UNCLEAR	LOW	LOW	

	Assessment justification:	Eligible participants were randomized on the basis of a computer automated randomization sequence after the acquisition of primary outcome data. Randomization was stratified by sex, age, and WC measurement	NS	Weight objectively measured.	Of the 490 participants, 396 (80.8%) returned for follow-up testing at 24 months.	
109	**Samaras 1997**	UNCLEAR	UNCLEAR	LOW	LOW	UNCLEAR
	Assessment justification:	'Subjects were randomised into two groups'<br><br>No further information given.	NS	All anthropometric measures were performed by a trained investigator (AMM). Body height was measured to the nearest cm using a stadiometer with the subject barefoot; body weight to the nearest 0.1 kg in light street clothing. B	Control:<br>13/13 at 12-months<br>Intervention:<br>13/13 at 12-months<br><br>0% dropout by end of study	After the 6-month programme, the exercise sessions remained available to subjects in the intervention group.
110	**Sattin 2016**	UNCLEAR	UNCLEAR	UNCLEAR	LOW	
	Assessment justification:	"Churches were recruited as pairs in the study based on congregation size. These pairs were included in six cohorts with each cohort including either two or four churches. Each church pair was then randomized to the Fit Body and Soul (FBAS) behavioral lifestyle intervention or Health Education (HE) comparison group."<br><br>No further information given.	Allocation concealment through pastor but no further detail given.	NS	No attrition.	
111	**Schubel 2016**	LOW	LOW	LOW	LOW	
	Assessment justification:	"They sequentially enter the study and are randomly allocated to the three dietary programs (ICR, CCR, or HD) by RANDI2 [9], a web-based software using a block size of six. Randomization is stratified by age (<50 years/ ≥ 50 years) and sex."	Refer to 'Random sequence generation (selection bias)'.	"All outcome assessments (see Table 2) are performed by trained study personnel following standard operating procedures."	Overall, 144 participants (96.0%) completed the 12-wk intervention phase, 143 (95.3%) the 12-wk maintenance phase, and 136 (90.7%) the 26-wk follow-up phase (Figure 1). Across the entire study period of 50 wk there were 4 dropouts in the ICR (91.8%), 7 in the CCR (85.7%), and 2 (96.2%) in the Control group.	
112	**Seligman 2011**	LOW	LOW	LOW	LOW	
	Assessment justification:	"Randomization was performed using a computer sequence with centrally concealed allocation."	Refer to 'Random sequence generation (selection bias)'.	"Body mass index was calculated as weight/height2 (kilograms per square meter).	Less than 25% attrition at 12-months follow-up.	

				Waist was measured between the last rib and the iliac crest. Body fat mass was assessed with bioelectrical impedance (Omron HBF 306 Bioimpedance Analyzer)."		
113	**Shikany 2013**	LOW	LOW	LOW	LOW	
	Assessment justification:	"At the baseline visit, eligible participants were randomly assigned to the MD or FB group via a pseudorandom number generator with a 1:1 allocation ratio"	"The allocated group was indicated on cards contained in sequentially numbered, opaque, sealed envelopes prepared in the Department of Biostatistics, UAB School of Public Health. To randomize a participant, the study coordinator opened the next consecutively numbered envelope in the presence of the participant."	"…fasting serum glucose concentration was assessed; height, weight and blood pressure were measured; Body weight was assessed at baseline and at the 26- and 52-week clinic visits as outcome measures (and at the 8-, 16-, 32- and 40-week clinic visits as a check of participant progress), with participants in light clothing and no shoes using a Tanita model BC-418 digital scale/body composition analyzer (Tanita Corporation of America, Inc., Arlington Heights, IL, USA)."	6-month retention rate:<br>FB: 49/60 = 81.6%;<br>MD: 56/60 = 93.3%<br><br>12-month retention rate:<br>FB: 56/60 = 93.3%;<br>MD: 57/60 = 95%<br><br>Food based: 45/60<br>Medifast: 50/60	
114	**Snel 2012**	UNCLEAR	UNCLEAR	LOW	LOW	
	Assessment justification:	NS	NS	"...patients visited the research center after an overnight fast. Height, weight and waist circumference were measured."	"All patients completed the whole study period of 18 months, there were no dropouts from the study."	
115	**Stevens 1993**	UNCLEAR	LOW	LOW	LOW	
	Assessment justification:	"At clinics using the weight reduction intervention, randomization was conducted within high- and low-weight strata, with only high-weight participants eligible for the weight reduction group."	Centralized allocation by telephone; if not possible, sealed opaque envelopes.	"In addition, weights and blood pressures were recorded for all participants during official clinic visits 3, 6, 12, and 18 months after they entered the study."	93% followed up at 12 months overall:<br>93% intervention;<br>93% control.<br>Reasons for attrition not reported.	
116	**Stevens 2001**	UNCLEAR	LOW	LOW	LOW	
	Assessment justification:	NS	Centralized allocation via telephone to central randomizing centre or via sealed opaque envelopes.	"Blood pressure and weight were measured every 6 months after randomization to the end of follow-up at 36, 42, or 48 months, depending on randomization date. Clinic staff	92% followed up at 18 months overall: 92% intervention, 92% control.<br>Reasons for attrition not reported.	

				who were blinded to study group assignment made these assessments."		
117	**Sundfor 2018**	LOW	LOW	LOW	LOW	
	Assessment justification:	"A statistician prepared a computer-generated random number list."	"The project leader (TS) opened numbered and sealed envelopes consecutively with no exception."	"Body weight was measured following a 10-h fast using the same calibrated digital scale to the nearest 0.1 kg."	"As shown in the Consolidated Standards of Reporting Trials flow chart four dropouts occurred in the intermittent versus three in the continuous energy restriction group." Greater than 90% of participants returned for all follow-up time points.	
118	**Tapsell 2017**	LOW	LOW	LOW	HIGH	
	Assessment justification:	"Randomisation was conducted after the second screen for eligibility and performed remotely by an investigator unrelated to the clinic using a computer generated randomisation sequence (STATA V12, StataCorp LP, College Station, TX). The randomisation was stratified according to sex and BMI (low BMI: ≤30 and high BMI: >30). Randomisation was performed in randomly allocated blocks of 3, 6 or 9. "	"The randomisation list was provided to the study team who added eligible participants sequentially for each of the strata. The randomisation and participant database was only accessible by the HealthTrack study co-ordinator and administrator for security."	"Body weight (kg) was measured in an upright position in minimal clothing and without shoes using scales with a bio-electrical impedance component to also estimate body fat (%) (Tanita TBF-662, Wedderburn Pty Ltd., Ingleburn, NSW, Australia)."	"The intensive phase was completed by 298 participants (withdrawal rate 18%) and the 12 months follow-up by n=178 participants (withdrawal rate 39%)."<br><br>Total sample withdrawal rate at 12 months = 178/377*100 = 47%<br>12-month follow-up rate per group: Control: 61/126*100 = 48%;<br>Intervention: 45/120*100 = 36%; Intervention plus walnut: 72/126*100 = 57%	
119	**TarragaMarcos 2017**	UNCLEAR	UNCLEAR	LOW	LOW	
	Assessment justification:	NS	NS	Weight objectively measured.<br><br>Blood pressure was measured using an automated and calibrated electronic device, according to the recommendations of the Spanish Society of Arterial Hypertension.	There were no dropouts in G1 or G2 during the follow-up period, however 4 patients left G3 for personal reasons, leaving this group with 55 patients.	
120	**Teeriniemi 2018**	LOW	UNCLEAR	LOW	LOW	

	Assessment justification:	"MS Excel was used by an independent researcher to produce a randomization list with random permuted blocks of 24."	NS	Weight was measured by a study nurse.	"A total of 108 participants (20.3%) did not return to the study centre for the 1-year visit (Fig. 2), and 49 participants dropped out between the 1-year and 2-year visits. Thus, 375 study subjects completed the study per protocol, and the attrition rate at 24 months was 29.5% (n = 157). No statistically significant differences amongst the dropouts were found between the study arms..."	
121	**ter Bogt 2009**	LOW	UNCLEAR	LOW	LOW	
	Assessment justification:	"... patients were allocated using computer-generated random numbers...".	NS	"Body weight was measured on an electronic scale with subjects wearing light clothing and no shoes..."	Low dropout rate after 1 year (9%).	
122	**Tsai 2010**	UNCLEAR	LOW	LOW	LOW	
	Assessment justification:	Randomization was blocked in groups of six.	Sealed envelopes.	Weight was assessed by a research assistant (B.J.I.), who was not masked to treatment assignment.	Control: 24/26 at 6mths; 25/26 at 12mths<br>Brief counselling: 21/24 at 6mths; 22/24 at 12mths	
123	**Tuomilehto 2009**	LOW	LOW	LOW	LOW	
	Assessment justification:	"…the subjects were allocated randomly to two study groups by a study nurse according to a previously generated randomization plan.	Randomised by study nurse who did not take part in subsequent intervention.	The weight was measured at every visit.	Control: 10% drop out<br>Intervention: 13% drop out	
124	**van de Glind 2017**	LOW	LOW	LOW	LOW	HIGH
	Assessment justification:	"The allocation sequence for each football club was generated by a computer programme written by a statistician not involved in the final analysis. The sequence was generated using randomised permuted blocks, stratified by club, with block lengths of 4 and 6, at random. The sequence was securely stored, with access restricted to those responsible for maintaining the randomisation system."	"Trial coordinators accessed randomisation allocation via a secure online portal."<br>"It was not possible to mask participants or the fieldwork team to allocation, but the primary outcome measurements could not be accessed by either, and allocation was not known by study statisticians until after database lock."	"Body weight was measured using an electronic flat scale (Tanita HD366) with light clothing."	91% and 92% of participants per group attended the post program follow-up time point; 88% and 92% attended the 12-month follow-up.	Wait-list control, no blinding.

125	**vanWier 2011**	LOW	LOW	LOW	LOW	
	Assessment justification:	After baseline measurements, the employee was randomised to one of the three study groups and either to a group receiving basic weight measurements (80% of each study group) or to a group receiving additional measurements (20% of each study group). This two-step randomisation meant that there were six groups an employee could be assigned to. Randomisation to these six groups was done by block randomisation, with each block containing 15 allocations. A computerized random number generator drew up an allocation schedule.	An administrative assistant put the group allocation in opaque sealed envelopes, numbered 1 to 1,500. These envelopes were taken to the locations of the baseline measurements and opened in the given order. The researchers were blinded for the allocation schedule, but were not blinded for allocation after randomisation.<br>The participants were, in consequence of the nature of the intervention, not blinded for allocation after randomisation. Employees were not allowed to change groups after randomisation.	At baseline 'body weight and body height were assessed by the researchers.'<br>'Body weight and body height are assessed in all participants. Body weight is measured in kg, to the nearest 0.1 kg, with a digital scale (Seca 770; Seca GmbH & Co, Hamburg, Germany). Participants are wearing light clothing and no shoes. Body height is measured in m, to the nearest 0.001 m, with a portable stadiometer (Seca 214, Leicester Height Measure; Seca GmbH & Co, Hamburg, Germany). ' LOW<br>In addition, in a questionnaire self-reported body weight is assessed. Participants are asked to weigh themselves wearing light clothing and no shoes. HIGH	At 24mths:<br>Control 266/460<br>Internet 263/464<br>Phone 263/462<br>all <50%	
126	**Vissers 2010**	UNCLEAR	UNCLEAR	LOW	LOW	
	Assessment justification:	NS	NS	Body weight was measured with a digital scale to the nearest 0.1 kg.	Less than 50% attrition at 12-month follow-up.	
127	**Volpe 2008**	UNCLEAR	UNCLEAR	LOW	UNCLEAR	HIGH
	Assessment justification:	"Participants were randomly assigned, in a stratified manner based on BMI, to one of three treatment conditions..."	NS	"Body weight was measured on a balance-beam scale accurate to 0.5 kg while the subject was wearing a swimsuit and no shoes."	NS	Raw data doesn't match up with information presented in graphs. Used raw data.
128	**Weinstock 2013**	UNCLEAR	UNCLEAR	LOW	LOW	
	Assessment justification:	NS	NS	"At baseline, 6 months, 1 and 2 years, a research nurse performed standardized assessments at the practice sites and measured height, weight..."	Percentage of sample followed up:<br>6-months:<br>CC: 71%; IC: 65%<br>1 Year:<br>CC: 62%; IC: 57%<br>2 Years:<br>CC: 56%; IC: 48%	

					3 year:<br>Total sample: 51.4%	
129	**West 2007**	UNCLEAR	LOW	LOW	LOW	
	Assessment justification:	NS	"women were randomized using a sequentially numbered, closed-envelope procedure."	"All assessments were conducted by trained interviewers blind to experimental condition. Body weight was measured without shoes using a calibrated balance beam scale."	Less than 20% and 50% of attrition at mid- and longer follow-ups.	
130	**The Look AHEAD Research Group 2010**	LOW	LOW	LOW	LOW	HIGH
	Assessment justification:	"Eligible participants are randomly assigned to either diabetes support and education or lifestyle intervention using a web-based data management system that verifies eligibility. Randomization is stratified by clinical center and blocked with random block sizes." (protocol)	"Eligible participants are randomly assigned to either diabetes support and education or lifestyle intervention using a web-based data management system that verifies eligibility. Randomization is stratified by clinical center and blocked with random block sizes." (protocol)	"Weight was measured in duplicate on a digital scale."	Retention rate:<br>Year 1:<br>DSE: 95.7%; ILI: 97.1%;<br>Year 2:<br>DSE: 93.5%; ILI: 94.9%;<br>Year 3:<br>DSE: 93.8%; ILI: 94.0%;<br>Year 4:<br>DSE: 93.0%; ILI: 94.1%;<br>Year 5:<br>DSE: 92.2%; ILI: 93.3%;<br>Year 6:<br>DSE: 90.6%; ILI: 92.0%;<br>Year 7:<br>DSE: 89.3%; ILI: 90.6%;	Participants in the intervention arm who, during the first 6 months, failed to lose 10% of their initial weight were offered a weight loss medication (orlistat). Those who lost <5% were encouraged by their lifestyle counselor to try pharmacotherapy, whereas those who lost 5.0% to 9.9% were informed of medication and could receive it on request. Medication was not offered to individuals who lost greater than or equal to 10% of initial weight

					Year 8:<br>DSE: 88.3%; ILI: 89.9%.	and maintain the loss.<br>523 out of 2570 participants in the ILI study arm took Orlistat as part of the intervention.
131	**Wing 1988**	UNCLEAR	UNCLEAR	LOW	LOW	UNCLEAR
	Assessment justification:	NS	NS	Weight was measured in street clothes, without shoes, using a balance beam scale.	Attrition less than 20% at program's end and less than 50% at 1-year follow-up	Participants have been omitted from the analysis both at baseline and 10-week program end.
131	**Wing 1988b**	UNCLEAR	UNCLEAR	LOW	LOW	
	Assessment justification:	NS	NS	Weight was measured in street clothes, without shoes, using a balance beam scale.	Less than 20% attrition at post-intervention follow-up and less than 50% of attrition at 1-year follow-up.	
132	**Wing 1991**	UNCLEAR	UNCLEAR	LOW	LOW	
	Assessment justification:	NS	NS	Weight was measured in street clothes, without shoes, using a balance beam scale.	Thirty-three (92%) of the 36 subjects completed both the posttreatment (week 20) and 1-year assessments (16/19 who entered the BT group and 17/17 who entered the VLCD group).	
133	**Wing 1998**	UNCLEAR	UNCLEAR	LOW	LOW	
	Assessment justification:	NS	NS	Weight objectively measured.	Less than 50% attrition at 12-month follow-up.	
134	**Yannakoulia 2008**	UNCLEAR	UNCLEAR	UNCLEAR	HIGH	UNCLEAR

	Assessment justification:	NS	NS	NS	Fifty percent of patients were dropouts. Comparisons between completers and dropouts revealed no statistically significant differences between the two groups (with regards to history of diabetes, sex, HbA1c, BMI or waist circumference), apart from their age, with those not completing the intervention being younger compared to completers (53 ± 9 vs. 60 ± 9 yr, $p = 0.05$) (Table 2). A trend for an association between group and dropout was observed: 66.7% in the UC and 33.3% in the IC were dropouts ($p = 0.07$).<br><br>To explore the effect of several factors in relation to the likelihood of being a dropout, a logistic regression was performed. Older people ($p = 0.03$) and those with newly diagnosed T2DM ($p = 0.05$) were more likely to complete the program, whereas a tendency for a negative association between attendance of the IC group and the likelihood of dropping out was found ($p = 0.08$).	"They were also informed about smoking risks and encouraged to stop or limit smoking" but no information on how many smoked and if smoking behaviour changed and no other mentions of smoking in the paper. This could be a potential confounder but without this information difficult to assess.
135	**Yates 2009**	LOW	LOW	LOW	LOW	
	Assessment justification:	"Participants were randomly assigned, using a block design, to receive either usual care, the PREPARE program, or the PREPARE program without pedometer use and were stratified by age and sex."	"Participant random assignment was conducted using opaque envelopes and a randomly generated number sequence by a member of our research team with no<br>prior knowledge of recruited individuals<br>other than their age and sex."	Weight measured objectively.<br>Waist circumference: midpoint between the lower costal margin and iliac crest;<br>Height: measured to the nearest 0.1 kg and 0.5cm, respectively.	Less than 50% attrition at 12-month follow-up.	
136	**Yeh 2016**	UNCLEAR	UNCLEAR	UNCLEAR	LOW	

	Assessment justification:	NS	NS	"Anthropometric measures and fasting blood specimens were obtained at baseline, 6 months and 12 months to evaluate weight and cardiometabolic changes."	Attrition was less than 50% follow-up and there was a <20% difference in follow-up between groups at 6m.	
137	**Yin 2018**	LOW	UNCLEAR	LOW	LOW	
	Assessment justification:	Participants were randomised in blocks of 10, using a randomization table by the study statistician.<br>Enrollment and randomization were performed by trained research staff.	NS	Trained research staff measured the participant's weight, height and waist circumference with light clothes twice and the average was used.<br>Participant's weight was recorded at each meeting.	Less than 50% of attrition (at 12 months, 19 int. and 5 cont. loss-to-follow-up)	
138	**Zhang 2016**	LOW	UNCLEAR	LOW	LOW	
	Assessment justification:	The randomization schedules were generated using SAS<br>PROC PLAN in SAS statistical software (SAS Institute Inc) and<br>concealed until an eligible participant was ready for enrollment.	NS	Weight measured objectively.	Of 220, 211 (95.9%) completed the 6-month follow-up visit, and 208 (94.5%) completed the 12-month follow-up visit.<br>ITT was followed, undertaking MCMC imputation method.	

*Ref No. Reference number in main paper.

BP: Blood pressure; **HDL-C:** High density lipoprotein cholesterol; **Mths:** Months; **NS:** Not specified; **PR**: Pulse rate; **RCT**: Randomised controlled trial; **Wk/s**: week/s; **Yr.:** Year; **Yrs:** Years.

Table S4. Characteristics of included studies

*Ref No. Reference number in main paper. †Not all outcome measures collected at all follow-up time points; Outcome measures collected at baseline only not listed.

BED: Binge Eating Disorder; **BMI:** Body Mass Index (kg/m^2); **CBT:** Cognitive Behaviour Therapy; **CV:** Cardiovascular; **DPP:** Diabetes Prevention Program; **FG:** Fasting glucose (including fasting plasma glucose and other glucose measures)**; HbA1c:** Haemoglobin A1C**; HDL:** High-density lipoprotein cholesterol; **N:** No; **HOMA-IR:** Homeostatic Model Assessment of Insulin Resistance**; HTN:** Hypertension; **MI:** Motivational interviewing; **MR:** Meal replacement; **N/A:** Not applicable; **NS:** Not specified; **QoL:** Quality of Life; **SBP:** Systolic Blood Pressure; **T2DM:** Type 2 Diabetes Mellitus; **TC:** Total cholesterol; **TC/HDL**: Total cholesterol/High-density lipoprotein ratio; **VLCD:** Very low calorie diet; **Y:** Yes.

Ref No.*	Study ID	Country:	Follow-up time points (months):	Population	Study groups and number of participants randomised	Outcome measures extracted†	Overall Risk of Bias	Author contacted	Additional information obtained from author:	Notes:
16	Abed 2013	Australia	3, 6, 9, 12, 15	Patients with symptomatic atrial fibrillation	Control = 75<br>Weight management = 75	Weight; TC; HDL; SBP; FBG; Plasma insulin	High	Y	N	
17	Ackermann 2011	USA	6, 14	DPP population	Standard advice alone (controls) = 46<br>YMCA DPP intervention = 46	Weight; TC; HDL; SBP; HBa1C	Unclear	N	N/A	
18	Ahern 2017	UK	3, 12, 24	Adults with a BMI ≥ 28	Brief intervention = 211<br>12-week behavioural weight-loss programme = 530<br>52-week behavioural weight-loss programme = 528	Weight; TC; SBP; HBa1C; QoL	Low	N	N/A	
19	Almanza - Aguilera 2018	Spain	3, 12	Metabolically healthy obese women (definition based on the general criteria proposed by the International Diabetes Federation (IDF))	Control (general recommendations) = 48<br>Treatment (lifestyle weight loss intervention) = 67	Weight; TC; HDL; SBP; FG	High	Y	Y	Information provided.
20	Andersen 1999	USA	1, 2, 3, 4, 16	Women with obesity	Diet + Lifestyle Activity = 20<br>Diet + Aerobic Group = 20	Weight; TC:HDL ratio; TC; HDL; SBP	Unclear	Y	N	
21	Anderson 2014	Scotland	3, 12	Overweight or obese adults (aged 50 to 74 years) who had undergone colonoscopy after a positive faecal occult blood test result, as part of the national bowel screening programme, and had a diagnosis of adenoma confirmed by histopathology.	Control (weight loss booklet only) = 166<br>Intervention (BeWEL) = 163	Weight; TC; HDL; SBP; HBa1C; HOMA-IR; FG; Plasma insulin	Low	N	N/A	
22	Appel 2011	USA	6, 12, 24	Adults who were at least 21 years of age with obesity and had one or more cardiovascular risk factors (hypertension, hypercholesterolemia, or diabetes).	Control (Self-directed) = 138<br>Remote Support Only (N/A) = 139<br>In-Person Support = 138	Weight; TC; SBP; FG; QoL	Low	N	N/A	Information available from previous reviews.
23	Ard 2018	USA	6, 12	General population of adults	Exercise Only = 54	Weight; TC; HDL;	Low	N	N/A	Information

				aged 65 and older who were at risk for cardiometabolic disease due to obesity and associated risk factors	Exercise + Diet Quality + Weight Maintenance = 55<br>Exercise + Diet Quality + Weight Loss = 55	SBP; FG; QoL; Incidence CV morbidity				available from previous reviews.
24	Ard 2004	USA	6, 18	The target population consisted of generally healthy adults with above optimal BP including individuals with stage 1 hypertension who met Joint National Committee on Detection, Evaluation, and Treatment of High Blood Pressure (JNC-VI) criteria for at least a 6-month trial of nonpharmacological therapy.	"advice only" comparison group = 273<br>"established" behavioural intervention group = 268<br>Established + DASH Intervention Group = 269	Weight; TC; SBP; FBG; Plasma insulin; QoL; Incidence HTN; Remission HTN	Low	N	N/A	Information available from previous reviews.
25	Ashley 2001	USA	0.46, 0.69, 0.92, 1.15, 1.38, 1.61, 1.84, 2.07, 2.3, 2.53, 2.76, 3, 3.22, 3.45, 3.68, 3.91, 4.14, 4.37, 4.6, 4.83, 5.06, 5.29, 5.52, 5.75, 6, 6.21, 6.44, 6.67, 6.9, 7.36, 7.83, 8.29, 8.75, 9.21, 9.67, 10.13, 10.59, 11.05, 11.51, 12, 24	General population (premenopausal women)	Control, Diet = 37<br>MR - Physician/Nurse led = 38<br>MR - Dietician lead = 38	Weight; TC; HDL; SBP; FG; Plasma insulin	High	N	N/A	Information available from previous reviews.
26	Azar 2013	USA	3, 6, 15, 24	Pre-diabetes and/or metabolic syndrome	Control, Usual care = NS<br>Self-directed = NS<br>Coach-led = NS	Weight; TC; SBP; FG	Low	Y	N	
27	Bacon 2002	USA	3, 6, 12, 24	Women from the general population	Health at Every Size – control = NS<br>Diet Group – intervention = NS	Weight; TC; HDL; SBP; QoL	Unclear	N	N/A	Information available from previous reviews.
28	Barnes 2017	USA	3, 6, 15	Overweight and obese with or without binge eating	Treatment as usual (N/A) = 30<br>Nutrition - ATTENTION CONTROL = 29<br>Motivational interviewing = 30	Weight; TC; HDL; SBP; HBa1c; FG	Unclear	Y	Y	Information provided.
29	Bartels 2015	USA	3, 6, 9, 12, 18	People with serious mental illness	Control, Fitness club membership = 106<br>IN SHAPE = 104	Weight; TC; HDL; SBP	Unclear	N	N/A	Information available

										from previous reviews.
30	Beeken 2017	England	3, 6, 12, 18, 24	General population with obesity	Usual care = 270<br>10TT = 267	Weight; TC; SBP; FBG	Low	Y	Y	Information provided.
31	Bennett 2013	USA	6, 12, 18	General population	Control, usual care = 97<br>Weight gain prevention intervention = 97	Weight; TC; HDL; SBP; FG; QoL	Unclear	Y	Y	Data provided.
32	Bennett 2012	USA	6, 12, 18, 24	Obese patients receiving hypertensive treatment	Control, Usual care = 185<br>Be Fit, Be Well = 180	Weight; SBP, QoL	Unclear	Y	Y	Data provided; Information available from previous reviews.
33	Bertz 2012	Sweden	3, 12	Women, 8-12-week post-partum	Control = 17<br>Diet Only = 17<br>Exercise only = 18<br>Intervention = 16	Weight; TC; SBP; FG; QoL	Unclear			Information available from previous reviews.
34	Bo 2007	Italy	12, 24	General population (70-72% with metabolic syndrome)	Control standard care = 188<br>Intervention lifestyle by trained professional = 187	Weight; TC; HDL; SBP; FG	Low	N	N/A	
35	Burke 2005	Australia	4, 16, 40	Hypertensive patients	Control usual care = 118<br>Low sodium + fish diet = 123	Weight; TC; HDL; SBP; HOMA-IR; FBG; Plasma insulin; QoL; Remission HTN	Unclear	Y	Y	Information provided.
36	Chaiyasoot 2018	Thailand	3, 8.75, 14.7	Obesity patients with metabolic syndrome	Control, Lifestyle Education Intervention = 52<br>Lifestyle Education Intervention plus Meal Replacements = 58	Weight; TC; HDL; SBP; HbA1c; HOMA-IR; FG; Plasma Insulin	Low	N	N/A	Information available from previous reviews.
37	Chee 2017	Malaysia	6, 12	Patients with type 2 diabetes	Usual Care = 115<br>tDNA Conventional Counseling = 57<br>tDNA Motivational Interviewing = 58	Weight; TC; HDL; SBP; HbA1c	Unclear	Y	N	Information available from previous reviews.
38	Cheskin 2008	USA	8, 20	Adult men and women with type 2 diabetes	Standard diet = 58<br>Meal replacement = 54	Weight; TC; HDL; SBP; HbA1c; FG; plasma insulin;	High	N	N/A	Information available from previous reviews.
39	Cheyette 2007	UK	4, 6, 12	Patients with type 2 diabetes on insulin treatment	Control = 20<br>Weight No More intervention group = 29	Weight; HbA1c; QoL	Unclear	N	N	
40	Christensen 2012	Denmark	3, 12	Female overweight healthcare	Reference group = 44	Weight; SBP	Low	N	N/A	

				workers	Intervention group = 54					
41	Cole 2013	USA	3, 12	Department of Defense beneficiaries enrolled in the TRICARE health care system living in the San Antonio, Texas, area; Diagnosis of pre - diabetes.	Control - individualised counselling = 31<br>Intervention- shared medical appointment = 34	Weight; TC; HDL; SBP; HbA1C; FG	Unclear	N	N/A	
42	Conroy 2015	USA	3, 12	General population	Self-guided = 50<br>Interventionist led = 49	Weight; SBP	Unclear	N	N/A	
43	Crowley 2017	USA	3.7, 7.4, 11.1	Veterans with type 2 diabetes	Group Medical Visit = 136<br>Intensive Weight Management Group Medical Visit = 127	Weight; TC; HDL; SBP; HbA1c	High	Y	N	Information provided.
44	Dalziel 2006	France	2, 12, 48	Patients who had experienced their first myocardial infarction.	Control = 303<br>Experimental = 302	Weight; TC; HDL; SBP; Final follow-up only: Incidence CV morbidity; Incidence CV mortality	High	N	N/A	
45	Damschroder 2014	USA	3, 12, 18, 24	Veterans	Control, MOVE - usual care = 159<br>ASPIRE group, individual telephone counselling = 162<br>ASPIRE group, group counselling = 160	Weight; HDL; SBP; HbA1c; QoL	Unclear	N	N/A	Information available from previous reviews.
46	Daubenmier 2016	USA	3, 6, 12, 18	Adults with obesity	Active control intervention = 94<br>Mindfulness Intervention = 100	Weight; TC; HDL: SBP; HbA1c;	Low	N	N/A	
47	Daumit 2013	USA	6, 12, 18	Psychiatric patients	Control, Usual care = 147<br>ACHIEVE = 144	Weight; TC; HDL; SBP; FG; QoL; One timepoint only: Plasma insulin; Incidence CV morbidity; Incidence T2DM	Unclear	N	N/A	Information available from previous reviews.
48	deVos 2016	Netherlands	6, 12, 18, 24, 30, 80	Females 50 to 60 years	Control = 204<br>Tailor-made lifestyle intervention = 203	Weight; TC; HbA1c; QoL	Unclear	Y	Y	Data provided.
49	Delahanty 2015	USA	6, 12	Patients with type 2 diabetes	Dietitian Referral group = 29<br>Group lifestyle intervention = 28	Weight; TC; SBP; HbA1c; One timepoint only: Remission HTN	High	Y	Y	Data provided.
50	Djuric 2002	USA	3, 6, 12	Women with stage I or II breast cancer diagnosed within the past 4 years and free of any recurrence.	Control = 13<br>Weight Watchers = 11<br>Individualized group = 13<br>Comprehensive group = 11	Weight; TC:HDL ratio; TC; HDL; FG; Plasma insulin	High	Y	N	Information available from previous reviews.
51	Duncan 2016	New Zealand	4, 12	Primary health care patients with an elevated 5-year cardiovascular disease risk	Control = 162<br>Intervention = 158	Weight; TC:HDL ratio; TC; HDL; SBP.	High	N	N/A	

52	Eakin 2014	Australia	6, 18, 24	Patients 20–75 years with type 2 diabetes	Usual care = 151<br>Telephone intervention = 151	Weight; TC:HDL ratio; TC; HDL; SBP; HbA1C	Low	Y	Y	Data provided
53	Fernandez-Ruiz 2018	Spain	6, 12, 24	General population (Community Care Centre population (health centre patients))	Control = 37<br>Intervention (healthy eating, exercise & CBT) = 37	Weight; TC; SBP; HbA1C; QoL; Incidence CV mortality	Unclear	N	N/A	
54	Fisher 2011	USA	6, 12	Community (overweight, premenopausal women)	Diet only = 29<br>Diet + aerobic training = 43<br>Diet + resistance training = 54	Weight; FG; Plasma insulin	Unclear	N	N/A	
55	Foley 2016	USA	6, 12	Obese (BMI: 30.0-44.9 kg/m2) community health center patients with a diagnosis of hypertension, diabetes and/or hyperlipidemia	Usual care (Control) = 175<br>Weight loss intervention = 176	Weight; TC; HDL; SBP; HbA1c; FG; QoL	Unclear	Y	Y	Data provided.
56	Foster-Schubert 2012	USA	6, 12	Post-menopausal women	Control- usual care = 87<br>Calorie reduced diet = 118<br>Aerobic exercise (N/A) = 117<br>Intervention - diet and exercise = 117	Weight; HOMA-IR; FG; Plasma insulin; QoL	Unclear	N	N/A	
57	Fuller 2012	Australia	1, 2, 3, 6, 9, 12	Male or female residents of inner western Sydney aged 18—65 years, with a BMI of 25—45 kg/m^2	Western diet group = 35<br>Korean diet group = 35	Weight; TC; HDL; SBP; FG; One timepoint only: Plasma Insulin	Unclear	Y	N	
58	Green 2015	USA	6, 12, 24	People taking antipsychotic medications	Usual care = 96<br>STRIDE = 104	Weight; HDL; SBP; FBG; QoL	Unclear	Y	N	Information available from previous reviews.
59	Hageman 2017	USA	6, 18, 30	General population (women from underserved rural communities)	Web-based only = 101<br>Web-based discussion = 100<br>Web-based email = 100	Weight; TC; HDL; SBP; FG	Low	Y	Y	Information provided; Information available from previous reviews.
60	Hardcastle 2013	UK	6, 18	Primary care patients	Control = 131<br>MI counselling intervention = 203	Weight; TC; HDL; SBP	Unclear	N	N/A	
61	Harrigan 2016	USA	6, 12	Breast cancer survivors	Usual Care Group = 33<br>Telephone Weight Loss Counseling = 34<br>In-Person Weight Loss Counseling = 33	Weight; FG	High	Y	Y	Data provided.
62	Hunt 2014	UK	3, 12	Male football fans	Control, Wait-list = 373<br>FFIT = 374	Weight; SBP; QoL	High	N	N/A	Information available from previous reviews.
63	Irwin 2003	USA	3, 12	General postmenopausal female population	Control Group = 86<br>Exercise group = 87	Weight; HOMA-IR; FG	Low	N	N/A	Information available

										from previous reviews.
64	Jebb 2011	Australia, Germany, UK	2, 4, 6, 9, 12, 18, 24	Adults with a BMI 27-35 and at least one additional risk factor for obesity-related disease	Standard care = 395<br>Commercial programme = 377	Weight; TC:HDL ratio; TC; SBP; FG; Incidence T2DM; Remission T2DM	Low	Y	Y	Data provided; Information available from previous reviews.
65	Jebb 2017	UK	3, 6, 12, 36	Obese adults seeking support to lose weight	Usual care = 140<br>Low energy total diet replacement programme = 138	Weight; TC; HDL; SBP; HbA1c; FG; Plasma insulin; QoL; One timepoint only: HOMA-IR	Low	N	N/A	
66	Jenkins 2017	Canada	6, 18	General population in the city of Toronto	Control = 486<br>Dietary advice only = 145<br>Food basket only = 148<br>Food and advice = 140	Weight; TC:HDL ratio; SBP; FG	High	N	N/A	
67	Katula 2013	USA	6, 12, 18, 24	People with pre-diabetes (fasting blood glucose=95 mg/dl ≤FBG ≤125)	Enhanced Usual Care Comparison Condition = 150<br>Lifestyle Weight-Loss Intervention = 151	Weight; HDL; SBP; HOMA-IR; Incidence T2DM	Low	Y	N	
68	Katzer 2008	New Zealand	2.3, 6.3, 14.3, 26.3	Women with at least one other cardiovascular risk factor.	Mail-delivered 'non-dieting' program (P3) = 101<br>Group 'non-dieting' program (P2) = 62<br>Group 'non-dieting' program plus Relaxation (P1) = 62	Weight; SBP	Unclear	N	N/A	
69	King 1989	USA	7, 12, 24	Men aged 30 - 59 years.	Control (N/A) = 52<br>Exercise only = 52<br>Diet only = 51	Weight; TC/HDL ratio; TC	High	N	N/A	
70	Knauper 2018	Canada	3, 12, 24	Individuals with overweight or obesity	Standard DPP = 101<br>Enhanced DPP = 107	Weight; TC/HDL ratio; SBP; HbA1c	High	Y	Y	Data provided.
71	Diabetes Prevention Program R G 2009	USA	6, 12, 18, 24, 30, 36, 42, 48, 54, 60, 66, 72, 78, 84, 90, 96, 102, 108, 114, 120, 126, 132, 138, 144, 150, 156, 162, 168, 174, 180, 186	People at high risk for type 2 diabetes (impaired glucose tolerance)	Placebo = 1082<br>Metformin (N/A) = 1073<br>Lifestyle = 1079	Weight; HDL; SBP; HbA1c; FG; QoL; Incidence T2DM	High			Information available from previous reviews.
72	Kuller 2012	USA	6, 18, 30, 48	Postmenopausal females	Control - health education = 255<br>Intervention - lifestyle change = 253	Weight; HDL; SBP; FG	Low	Y	Y	Information provided. Information available from previous

										reviews.
73	Kumanyika 2012	USA	12, 24	General population (65% African American, non-Hispanic black)	Basic programme = 137<br>Basic plus programme = 124	Weight; SBP	Low	N	N/A	Information available from previous reviews.
74	Ley 2004	New Zealand	6, 12, 24, 36, 60	Workers with impaired glucose tolerance ((2 h blood glucose 7.8–11.0 mmol/l) and a further 114 (2%) had high normal blood glucose concentrations (7.0–7.8 mol/l))	Control diet = 70<br>Reduced-fat = 66	Weight; TC/HDL ratio; TC; HDL; SBP	High	N	N/A	
75	Li 2016	China	1, 12	Adults with Type 2 Diabetes Mellitus who are overweight (BMI ≥ 24 kg/m2)	Usual care group = 60<br>Diet group = 79<br>50g-oats group = 80<br>100g-oats group = 79	Weight; TC; HbA1c	Unclear	Y	N	
76	Li 2005	USA	0.5, 1.5, 2, 3, 4, 5, 6, 7, 8, 9, 10, 11, 12	Adults previously diagnosed and being treated for type II Diabetes Mellitus who are obese	Individualized diet plan = 52<br>Soy-based meal replacement = 52	Weight; TC; HbA1c	High	Y	N	
77	Lindstrom 2003	Finland	12, 24, 36, 48, 60, 72, 86, 96, 108, 120	Impaired glucose tolerance (IGT); People at high risk for type 2 diabetes	Control = 257<br>Intervention = 265	Weight; TC/HDL ratio; TC; HDL; SBP; HbA1c; FBG; Incidence DM; Incidence CVD morbidity; One timepoint only: Plasma insulin	High	Y	N	Information available from previous reviews.
78	Liss 2016	USA	6, 12	Adults with type 2 diabetes and a BMI ≥ 24 kg/m2	Standard care arm = 167<br>Standard care plus group-based lifestyle intervention = 164	Weight; TC; SBP; HbA1c	Low	Y	Y	Data provided.
79	Little 2016	UK	6, 12	General population	Control, Nurse follow-up = 279<br>Web-based support with minimal support (Remote) = 270<br>Web-based + nurse support (face to face) = 269	Weight; TC; HDL; SBP; HbA1c; FG; QoL	Unclear	N	N/A	Information available from previous reviews.
80	Manning 1994	UK	3, 6, 12, 48	Diabetic males and females	Clinic visit = 37<br>Behavioural = 38<br>Home visits = 35<br>Dexfenfluramine (N/A) = NS<br>Routine usual care (N/A) = NS	Weight; HbA1c	Unclear	N	N/A	
81	Mefferd 2007		4, 12	Adult breast cancer survivors with a BMI ≥ 25.0 kg/m^2	Control = 29<br>Intervention = 56	Weight; TC	Unclear	Y	Y	Data provided; Information available from previous reviews.

82	Melchart 2017	Germany	3, 6, 9, 12	Adults aged 18–67 years who are moderately overweight	Control group = 57<br>Intervention group = 109	Weight; TC/HDL ratio; SBP: FG	Low	Y	Y	Data provided.
83	Melin 2003	Sweden	3, 6, 12, 24	Obesity with complication diagnoses (i.e. diabetes type 2, hypertension, dyslipoproteinemia, polycystic ovary disease and apnoea disorder).	Control, less intensively treated = 21<br>Intensively treated = 22	Weight; SBP; FG; Plasma insulin	Unclear	N	N/A	Information available from previous reviews.
84	Menard 2005	Canada	6, 12, 18	Patients with type 2 diabetes	Control - usual care = 36<br>Intervention - intensive multitherapy = 36	Weight; TC/HDL ratio; SBP; HbA1c; QoL	Unclear	Y	N	Information available from previous reviews.
85	Mengham 1999	UK	6, 12	Patients with diabetes, aged less than 75 years, with BMI above 25kg/m^2	Control = NS<br>Intervention = NS	Weight; TC	Unclear	N	N/A	Information available from previous reviews.
86	Mensinger 2016	USA	6, 24	General population	Control, Weight Neutral Program = 40<br>Weight Loss Program = 40	Weight; TC/HDL ratio; TC; HDL; SBP; FG; QoL	High	N	N/A	Information available from previous reviews.
87	Mitsui 2008	Japan	3, 12	50-69-year-old adults	Control = 22<br>Intervention = 24	Weight; TC; HDL; SBP; FG	Unclear	Y	N	
88	Moreno 2014	Spain	0.5, 2, 4, 6, 8, 10, 12, 18, 24	Patients with obesity and prediabetes attending a hospital obesity unit (Obesity Unit, Hospital Gregorio Maranon, Madrid)	Low-calorie diet = 39<br>Very low-calorie-ketogenic diet = 40	Weight; TC; HDL; HbA1c; FG	Unclear	Y	N	Information available from previous reviews.
89	Morgan 2010	Australia	3, 6, 12	Males 18-60 years of age who are overweight or obese.	Control (Information and self-help) = 31<br>SHED-IT (Internet) group = 34	Weight; SBP	Low	N	N/A	Information available from previous reviews.
90	Muggia 2014	Italy	6, 12	Overweight and obese patients	Standard care group = 83<br>Brief CBT group = 80	Weight; TC; HDL; SBP; HOMA-IR; FG; Plasma insulin	High	N	N/A	
91	Nakata 2014	Japan	3, 6, 18, 30	Japanese adults	Control (N/A) = 63<br>Education-only = 62<br>Group-based support = 63	Weight; HDL; SBP; FG	Unclear	Y	Y	Data provided.
92	Nanchahal 2012	UK	6, 12	Adults with BMI ≥ 25 kg/m2	Usual care control = 190<br>CAMWEL Intervention = 191	Weight; SBP; QoL	Unclear	Y	Y	Data provided; Information available from previous reviews.

93	Ng 2015	UK	4, 12	Chinese patients with moderate to severe obstructive sleep apnoea (OSA) diagnosed on portable home sleep monitoring.	Control group = 43<br>Lifestyle modification program = 61	Weight; TC; FG; QoL	Unclear	Y	Y	Data provided.
94	Nilsen 2011	Norway	6, 12, 18	Individuals at high risk for type 2 Diabetes	Control, Individual Physician Group = 104<br>Individual Plus Interdisciplinary Group = 109	Weight; TC; HDL; SBP; HbA1c; FG	Low	Y	Y	Data provided; Information available from previous reviews.
95	Nordby 2012	Denmark	3, 9, 15	Younger (age: 20–40 years), sedentary, and only moderately overweight (BMI: 25–30 kg/m^2) men.	Control = 15<br>Training and increased diet (N/A) = 13<br>Training = 17<br>Energy-reduced diet = 15	Weight; TC; SBP; HbA1c	High	Y	Y	Data provided.
96	Oldroyd 2006	UK	6, 12, 24	Men and women of European origin	Control group = 39<br>Intervention group = 39	Weight; TC; FG	Unclear	N	N/A	Information available from previous reviews.
97	Pan 1997	China	24, 48, 72, 96, 120, 144, 168, 192, 216, 240 252, 264, 276, 288, 360	Chinese participants with impaired glucose tolerance	Control = 138<br>Intervention group (Exercise: n=155; Diet: n = 148; Diet plus exercise: n = 135) = 438	Weight; TC; SBP; FG; Incidence CV morbidity; Incidence CV mortality; Incidence T2DM; One timepoint only: HbA1c	High	Y	N	
98	Parikh 2010	USA	3, 6, 12	Adults with BMI ≥ 25 kg/m^2 and prediabetes	Control = 49<br>Intervention = 50	Weight; SBP; HbA1c	Unclear	Y	N	
99	Pedersen 2013	Copenhage n	3, 12	Adults with stable coronary artery disease who are overweight or obese	Aerobic interval training = 35<br>Low energy diet = 35	Weight; TC/HDL ratio; SBP; HbA1c	Unclear	N	N/A	
100	Pettman 2009	Australia	4, 12	Adults with metabolic syndrome	Control = 50<br>Intervention B - Passive follow-up = 54<br>Intervention A - Active follow-up = 49	Weight; TC; SBP; HOMA-IR; One timepoint only: FG	Low	Y	Y	Data provided.
101	Promrat 2010	USA	3, 6, 9, 12	Adults who were overweight or obese and diagnosed with Nonalcoholic steatohepatitis	Control = 10<br>Lifestyle Intervention = 21	Weight; TC; HbA1c; HOMA-IR	Low	Y	Y	Data provided.
102	Provencher 2009	Canada	4, 10, 16	Premenopausal women	Control group = 48<br>Social support = 48<br>Health-At-Every-Size = 48	Weight; TC; One timepoint only: HDL;	Unclear	Y	Y	Data provided.
103	Ridgeway 1999	USA	6, 12	Patients with Type 2 Diabetes	Control = 28<br>Intervention Group = 28	Weight; TC; HbA1c; FBG	Unclear	N	N/A	
104	Rock 2015	USA	6, 12, 18, 24	Patients with early-stage breast cancer	Control = 349<br>Intervention = 348	Weight; SBP	Low	N	N/A	
105	Rolls 2005	USA	0.92, 1.8, 2.8, 3.7, 4.6, 5.5,	Overweight and obese women and men	Comparison-control = 50<br>Two snacks = 50	Weight; TC; SBP	Unclear	Y	Y	Data provided.

			6.4, 7.4, 8.3, 9.2, 10.1, 11, 12		One soup = 50<br>Two soups = 50				
106	Rolls 2017	USA	1, 2, 3, 4, 5, 6, 7, 8, 9, 10, 11, 12	Women aged 20–65 years BMI of 28–45 kg/m2	Standard advice = 62<br>Pre-portioned foods group = 62<br>Portion selection group = 62	Weight; TC; SBP; HOMA-IR; FG	Unclear	Y	Y	Data provided; Information available from previous reviews.
107	Rosas 2015	California	6, 12, 24	Participants are obese Spanish-speaking adults with at least one cardiovascular risk factor recruited from a community health center in a low-income neighborhood of San Mateo County, California.	Usual care = 41<br>Case-management intervention = 84<br>Case-management + Community health worker intervention = 82	Weight; TC; HDL; SBP; HbA1c; FG	Unclear	N	N/A	
108	Ross 2012	Canada	6, 12, 18, 24	General population	Control condition = 241<br>Behavioral intervention group = 249	Weight; TC; HDL; SBP; FG	Unclear	N	N/A	Information available from previous reviews.
109	Samaras 1997	Australia	6, 12	Mature-aged people, performing less than 1 hour of exercise per week	Control =13<br>Intervention = 13	Weight; TC; HDL; HbA1c; FG; Plasma insulin	Unclear	N	N/A	Information available from previous reviews.
110	Sattin 2016	USA	3, 12	Obese and overweight, and/or prediabetic (FPG of 100 mg/dl to 125 mg/dl).	Health Education intervention = 287<br>Fit body and soul intervention = 317	Weight; FBG	Unclear	N	N/A	
111	Schubel 2016	Germany	3, 5.5, 11.5	Adults between 35-65 years, non-smokers and who are overweight or obese.	Control group = 52<br>Continuous Calorie Restriction = 49<br>Intermittent Calorie Restriction = 49	Weight; TC; HbA1c; HOMA-IR	Low	Y	Y	Data provided.
112	Seligman 2011	Brazil	3, 12	General population (patients with metabolic syndrome - no diabetics, more than half of the participants were hypertensive)	Standard-of-care strategy = 25<br>Healthy diet and step counter = 25<br>Healthy diet and fitness = 26	Weight; TC; HDL; SBP; HOMA-IR	Low	Y	Y	Data provided; Information available from previous reviews.
113	Shikany 2013	USA	6, 12	General population	Food-based diet = 60<br>Meal replacement = 60	Weight; TC; HDL; SBP; FG	Low	N	N/A	Information available from previous reviews.
114	Snel 2012	The Netherlands	4, 22	Adults with insulin-dependent Type 2 diabetes mellitus and obesity	VLCD only = 14<br>VLCD + exercise = 13	Weight; HbA1c; QoL	Unclear	N	N/A	
115	Stevens 1993	USA	3, 6, 12, 18, 276	Men and women aged 30 to 54	Control = 256	Weight; SBP	Unclear	Y	Y	Data

				years with high-normal diastolic blood pressure from 80 through 89 mm hg.	Intervention = 308					provided.
116	Stevens 2001	USA	6, 12, 18, 24, 30, 36	Adults 30 to 54 years of age who had nonmedicated diastolic blood pressure of 83 to 89 mm Hg and systolic blood pressure less than 140 mm Hg and were 110% to 165% of their ideal body weight at baseline.	Control = 596<br>Intervention = 595<br>Sodium only intervention (N/A) = 594<br>Combined intervention (N/A) = 597	Weight; SBP	Unclear	Y	Y	Data provided. Information available from previous reviews.
117	Sundfor 2018	Norway	3, 6, 12	Men and women aged 21-70 years with BMI 30-45.0	Continuous energy restriction = 58<br>Intermittent energy restriction = 54	Weight; TC; SBP; HbA1c; FG	Low	N	N/A	
118	Tapsell 2017	Australia	3, 12	Adult residents, 25-54 years, BMI 25-40kg/m2	Usual care (Control) = 126<br>Intervention Group = 125<br>Intervention plus food supplement group (N/A) = 126	Weight; TC/HDL ratio; TC; SBP; HbA1c; QoL; Remission HTN	High	N	N/A	
119	TarragaMarcos 2017	Spain	0.5, 1, 3, 6, 12	Adult general obese/overweight population.	G3 = 55<br>G2 = 61<br>G1 = 60	Weight; TC; HDL	Unclear	Y	N	
120	Teeriniemi 2018	Finland	12, 24	Residents aged 20–60 years living in the city of Oulu who were overweight or obese.	Control = 89<br>SHG Counselling = 87<br>CBT Counselling = 85<br>Control plus HBCSS = 91<br>SHG Counselling plus HBCSS = 92<br>CBT Counselling plus HBCSS = 88	Weight; HDL; SBP; FG	Unclear	Y		
121	ter Bogt 2009	Netherlands	12, 36	Patients 40–70 years of age with BMI: 25 to 40 and either hypertension or dyslipidemia or both.	GP usual care = 232<br>Lifestyle counselling from NP = 225	Weight; TC; HDL; SBP; FG	Unclear	N	N/A	
122	Tsai 2010	USA	3, 6, 12	General population	Control = 26<br>Brief counselling = 24	Weight; TC; HDL; SBP; FG	Unclear	N	N/A	Information available from previous reviews.
123	Tuomilehto 2009	Finland	3, 12, 24, 60	Patients with mild obstructive sleep apnoea	Control = 41<br>Intervention = 40	Weight; TC; HDL; SBP; FBG; Plasma insulin; QoL	Low	N	N/A	Information available from previous reviews.
124	van de Glind 2017	England, The Netherland, Norway, Portugal	3, 12	Males	Comparison group = 553<br>EuroFIT group = 560	Weight; TC; SBP; HbA1c; QoL; Final timepoint only: Incidence CV morbidity	High	Y	Y	Information /data received.
125	vanWier 2011	Netherlands	6, 24	General population	Control – Brochure = 460<br>Internet Group = 464<br>Phone Group = 462	Weight; TC; SBP	Low	N	N/A	

126	Vissers 2010	Belgium	3, 6, 12	General overweight or obesity patients.	Control = 21<br>Diet only group (Diet) = 20<br>Diet + fitness training group (Fitness) = 20<br>Diet + WBV group (Vibration) = 18	Weight; HDL; SBP; FBG; Final follow-up only: Incidence T2DM	Unclear	N	N/A	Information available from previous reviews.
127	Volpe 2008	USA	3, 6, 9, 12	Adults with overweight/obesity	Exercise only = 34<br>Diet only = 28<br>Combination of diet and exercise = 28	Weight; TC/HDL ratio; TC; SBP	High	Y	Y	Data provided.
128	Weinstock 2013	USA	6, 12, 24, 36	Adults with metabolic syndrome	Conference Call DPP = 128<br>Individual Call DPP = 129	Weight; TC; SBP; FG	Unclear	Y	N	
129	West 2007	USA	6, 12, 18	Women with type 2 diabetes treated by oral diabetes medications but not insulin	Attention control = 108<br>Motivational interviewing = 109	Weight; HbA1c	Unclear	N	N/A	
130	The Look AHEAD Research Group 2010	USA	12, 24, 36, 48, 60, 72, 84, 96, 108, 115, 120	Adults with Type 2 Diabetes Mellitus	Diabetes support and education = 2575<br>Intensive lifestyle intervention = 2570	Weight; TC; HDL; SBP: QoL; Incidence CV morbidity; Incidence CV mortality; Incidence T2DM; Remission T2DM	High	N	N/A	Information available from previous reviews.
131	Wing 1988	USA	2, 14	Type 2 diabetes	Diet plus placebo exercise = 13<br>Diet plus moderate exercise = 12	Weight; TC; HDL; SBP; HbA1c	Unclear	N	N/A	
131	Wing 1988b	USA	2, 14	Type 2 diabetes patients	Diet only = 15<br>Diet plus exercise = 15	Weight; TC; HDL; SBP; HbA1c; FG; Plasma insulin	Unclear	N	N/A	
132	Wing 1991	USA	5, 17	Overweight and obese people with type 2 diabetes	Behavior therapy alone = 19<br>Behavior therapy plus VLCD = 17	Weight; TC/HDL ratio; TC; HDL; HbA1c; FG	Unclear	N	N/A	Information available from previous reviews.
133	Wing 1998	USA	6, 12, 24	Overweight participants who had one or two parents with diabetes	Control = 40<br>Diet = 37<br>Exercise = 37<br>Diet plus exercise = 40	Weight; TC/HDL ratio; TC; HDL; SBP; HbA1c; FG; At final follow-up only: Incidence T2DM	Unclear	N	N/A	Information available from previous reviews.
134	Yannakoulia 2008	Greece	2, 12	Type 2 diabetes mellitus patients	Usual care group = 15<br>Intensive care group = 15	Weight; HbA1c	High	Y	Y	Data provided; Information available from previous reviews.
135	Yates 2009	UK	3, 6, 12, 24	Patients with impaired glucose tolerance	Control group = 34<br>PREPARE with pedometer = 33<br>PREPARE group = 31	Weight; TC; HDL; SBP; FG; At final follow-up only: Incidence T2DM	Low	N	N/A	Information available from previous reviews.

136	Yeh 2016	USA	6, 12	Chinese immigrants with prediabetes living in New York City	Control group = 30<br>Intervention group = 30	Weight; TC; SBP; HbA1c	Unclear	Y	N	Information available from previous reviews.
137	Yin 2018	China	6, 12	Women with pre-diabetes	Comparison-Control Group = 75<br>Intervention Group = 109	Weight; HbA1c; FG	Unclear	N	N/A	
138	Zhang 2016	China	6, 12, 24	Patients with Nonalcoholic Fatty Liver Disease	Control = 74<br>Moderate exercise = 73<br>Vigorous-moderate exercise = 73	Weight; TC; HDL; SBP; FG	Unclear	N	N/A	Information available from previous reviews.

*Ref No. Reference number in main paper.
†Not all outcome measures collected at all follow-up time points; Outcome measures collected at baseline only not listed.

BED: Binge Eating Disorder; **BMI:** Body Mass Index (kg/m^2); **CBT:** Cognitive Behaviour Therapy; **CV:** Cardiovascular; **DPP:** Diabetes Prevention Program; **FG:** Fasting glucose (including fasting plasma glucose and other glucose measures)**; HbA1c:** Haemoglobin A1C**; HDL:** High-density lipoprotein cholesterol; **N:** No; **HOMA-IR:** Homeostatic Model Assessment of Insulin Resistance**; HTN:** Hypertension; **MI:** Motivational interviewing; **MR:** Meal replacement; **N/A:** Not applicable; **NS:** Not specified; **QoL:** Quality of Life; **SBP:** Systolic Blood Pressure; **T2DM:** Type 2 Diabetes Mellitus; **TC:** Total cholesterol; **TC/HDL**: Total cholesterol/High-density lipoprotein ratio; **VLCD:** Very low calorie diet; **Y:** Yes.

Table S5. Baseline demographics

*Ref No. Reference number in main paper. †Comorbidity definitions varied for each study; ‡Unclear whether DM percentage listed includes Type II and Type I;
§ Median (IQR); ‖ Standard error; # Range; ** 95% Confidence intervals
CBT: Cognitive Behaviour Therapy; **CV** = Cardiovascular; **DM:** Diabetes Mellitus; **DPP:** Diabetes Prevention Program**; N/A** = Not applicable; **NR** = Not reported; **VLCD:** Very low calorie diet

**Study ID** Ref No.*	**Groups:**	**Randomised**	**Number of participants reported at baseline**	**Gender (%F)**	**Age**		**BMI**		**Comorbidities at baseline (%)**†		
					Mean	***SD***	***Mean***	***SD***	***CV morbidity***	***Type II DM***	***Hypertension***
Abed 2013[16]	Control	75	75	33	60.3	10.3	33.8	4.1	100	28	87
	Weight Management	75	75	32	59.8	9.5	32.8	3.5	100	24	83
Ackermann 2011[17]	Standard advice alone (controls)	46	46	61	60.1	10.5	30.8	5.1	NR	NR	NR
	YMCA DPP intervention	46	46	50	56.5	9.7	32.0	4.8	NR	NR	NR
Ahern 2017[18]	Brief intervention	211	211	68	51.9	14.1	34.4	4.6	NR	13.5	49.8
	12-week behavioural weight-loss programme	530	528	68	53.6	13.3	34.7	5.4			
	52-week behavioural weight-loss programme	528	528	68	53.3	14.0	34.5	5.1			
Almanza - Aguilera 2018[19]	Control (general recommendations)	48	27	100	44.4	3.3	36.3	5.7	NR	0	NR
	Treatment (lifestyle weight loss intervention)	67	30	100	45.7	3.5	35.4	4.1	NR	0	NR
Andersen 1999[20]	Diet + Lifestyle Activity	20	20	100	42.9	7.9	32.4	4.5	NR	NR	NR
	Diet + Aerobic Group	20	20	100	43.2	9.1	31.4	3.7	NR	NR	NR
Anderson 2014[21]	Control (weight loss booklet only)	166	166	26	63.6	6.7	30.4	3.9	NR	14.3	NR
	Intervention (BeWEL)	163	163	26	63.5	7.0	31.0	4.5	NR		NR
Appel 2011[22]	Control (Self-directed)	138	138	63.8	52.9	10.1	36.8	5.1	NR	23.8	76.8
	Remote Support Only (N/A)	139	N/A	N/A	N/A	N/A	N/A	N/A	N/A	N/A	N/A
	In-Person Support	138	138	63.8	53.3	10.5	36.8	5.2	NR	23.9	71.0
Ard 2018[23]	Exercise Only	54	54	68.5	69.9	4.5	33.9	0.4	NR	NR	NR
	Exercise + Diet Quality + Weight Maintenance	55	55	60	70.5	4.8	33.8	0.4	NR	NR	NR
	Exercise + Diet Quality + Weight Loss	55	55	58.2	70.3	4.8	33.3	0.4	NR	NR	NR
Ard 2004[24]	"advice only" comparison group	273	273	63	49.5	8.8	32.9	5.6	NR	NR	14.0
	"established" behavioural intervention group	268	268	64.9	50.2	8.6	33.0	5.5	NR	NR	13.7
	Established + DASH Intervention Group	269	269	57.2	50.2	9.2	33.3	6.3	NR	NR	13.8
Ashley 2001[25]	Control, Diet	37	37	100	42.3	4.1	29.9	2.6	NR	NR	NR
	MR - Physician/Nurse led	38	38	100	41	5.7	30.1	3.7	NR	NR	NR
	MR - Dietician lead	38	38	100	41	4.3	30.1	2.9	NR	NR	NR
Azar 2013[26]	Control, Usual care	NR	81	45.7	52.5	10.6	32.4	6.3	NR	NR	NR
	Self-directed	NR	81	45.7	51.8	9.9	31.7	4.7	NR	NR	NR
	Coach-led	NR	79	48.1	54.6	11.0	31.8	5.1	NR	NR	NR
Bacon 2002[27]	Health at Every Size - control	NR	29	100	39.3	4.5	35.9	4.1	NR	NR	NR
	Diet Group - intervention	NR	23				36.6	4.1	NR	NR	NR
Barnes 2017[28]	Treatment as usual (N/A)	30	30	N/A	N/A	N/A	N/A	N/A	N/A	N/A	N/A

Study ID Ref No.*	Groups:	Randomised	Number of participants reported at baseline	Gender (%F)	Age		BMI		Comorbidities at baseline (%)†		
					Mean	*SD*	*Mean*	*SD*	*CV morbidity*	*Type II DM*	*Hypertension*
	Nutrition - ATTENTION CONTROL	29	29	69	48.9	11.6	35.1	7.5	NR	NR	NR
	Motivational interviewing	30	30	80	47.1	10.0	34.7	7.1	NR	NR	NR
Bartels 2015[29]	Control, Fitness club membership	106	106	55	43.5	11.6	37.5	8.8	NR	NR	NR
	IN SHAPE	104	104	47	44.3	10.9	36.2	7.5	NR	NR	NR
Beeken 2017[30]	Usual care	267	267	64.8	60§	48.9-67.1§	34.8§	32.6-39.4§	NR	NR	NR
	10TT	270	270	66.7	59.1§	48.1-66.1§	35§	32.6-38.7§	NR	NR	NR
Bennett 2013[31]	Control, usual care	97	94	100	35.2	5.5	30.2	2.4	NR	5.3	36.2
	Weight gain prevention intervention	97	91	100	35.6	5.5	30.1	2.7	NR	5.8	36.3
Bennett 2012[32]	Control, Usual care	185	185	65.9	54.7	11.0	36.99	5.2	NR	NR	NR
	Be Fit, Be Well	180	180	71.1	54.6	10.8	37.03	5.0	NR	NR	NR
Bertz 2012[33]	Control	17	17	100	32.2	4.6	30.2	3.4	NR	NR	NR
	Diet Only	17	17	100	33.7	4.2	33.7	2.6	NR	NR	NR
	Exercise only	18	18	100	33.2	3.7	33.2	3.1	NR	NR	NR
	Intervention	16	16	100	33.9	4.5	33.9	2.2	NR	NR	NR
Bo 2007[34]	Control standard care	188	166	57.8	55.7	5.6	29.8	4.6	NR	NR	36.1
	Intervention lifestyle by trained professional	187	169	58.6	55.7	5.7	29.7	4.1	NR	NR	36.1
Burke 2005[35]	Control usual care	118	118	57	55.3	7.5	29.7	2.5	NR	NR	100
	Low sodium + fish diet	123	123	54.5	57.1	7.2	30.4	2.9	NR	NR	100
Chaiyasoot 2018[36]	Control, Lifestyle Education Intervention	52	52	78.8	43.2	11.9	33.1§	30, 38.3§	NR	NR	NR
	Lifestyle Education Intervention plus Meal Replacements	58	58	86.2	41.8	11.8	32§	30.4, 37.6§	NR	NR	NR
Chee 2017[37]	Usual Care	115	115	48.7	54	8	28.9	6.3	NR	100	NR
	tDNA Conventional Counseling	57	57	87.4	55	8	29.4	7.3	NR	100	NR
	tDNA Motivational Interviewing	58	58	67.2	55	8	30.7	8.2	NR	100	NR
Cheskin 2008[38]	Standard diet	58	58	58.6	55.48	7.2	35.7	3.8	NR	100	NR
	Meal replacement	54	54	53.7	54.6	7.0	35.3	3.5	NR	100	NR
Cheyette 2007[39]	Control	20	20	40	58	10.7	31.7	5.4	NR	100	NR
	Weight No More intervention group	29	29	51.7	56.7	9.7	34.1	4.7	NR	100	NR
Christensen 2012[40]	Reference group	44	44	100	46	8.6	30.4	4.9	NR	NR	NR
	Intervention group	54	54	100	45.7	6.36	30.5	5.4	NR	NR	NR
Cole 2013[41]	Control - individualised counselling	31	31	51	55	9.9	31.4	4.8	NR	NR	19
	Intervention- shared medical appointment	34	34	41	61.2	8.4	30.3	5	NR	NR	25
Conroy 2015[42]	Self-guided	50	49	100	54	5.6	33.4	5.4	NR	23.5	56.1
	Interventionist led	49	49	100	53.8	5.3	36.1	5.4	NR		
Crowley 2017[43]	Group Medical Visit	136	136	8.1	60.4	8.3	35	4.8	75.7	100	87.5
	Intensive Weight Management Group Medical Visit	127	127	13.4	61	8.1	35.6	5.4	83.5	100	91.3
Dalziel 2006[44]	Control	303	303	7.9	53.5	10	25.8	3.4	100	NR	NR
	Experimental	302	302	10.6	53.5	10	25.8	3.4	100	NR	NR

Study ID [Ref No.*]	Groups:	Randomised	Number of participants reported at baseline	Gender (%F)	Age		BMI		Comorbidities at baseline (%)[†]		
					Mean	*SD*	*Mean*	*SD*	*CV morbidity*	*Type II DM*	*Hypertension*
Damschroder 2014[45]	Control, MOVE - usual care	159	159	12.6	54.6	10.5	36.8	6.4	NR	37.7	65.4
	ASPIRE group, individual telephone counselling	162	162	16	55.4	10.0	36.3	6.2	NR	32.7	67.9
	ASPIRE group, group counselling	160	160	16.2	54.9	9.5	36.2	6.1	NR	40.0	65.6
Daubenmier 2016[46]	Active control intervention	94	94	86	47.8	12.4	35.6	3.8	NR	NR	22.3
	Mindfulness Intervention	100	100	79	47.2	13.0	35.4	3.5	NR	NR	16
Daumit 2013[47]	Control, Usual care	147	147	49	44.1	11.0	36.5	7.3	NR	NR	NR
	ACHIEVE	144	144	51.4	46.6	11.5	36	7.2	NR	NR	NR
deVos 2016[48]	Control	204	204	100	55.7	3.2	32.5	4.5	NR	NR	74.5
	Tailor-made lifestyle intervention	203	203	100	55.7	3.2	32.2	4.1	NR	NR	68.5
Delahanty 2015[49]	Dietitian Referral group	29	29	41	61	11.4	33.8	5.0	27.6	100	82.6
	Group lifestyle intervention	28	28	39	62	9.6	36.3	12.4	25.0	100	71.4
Djuric 2002[50]	Control	13	48	100	51.7	8.4	34.9	1.2[‖]	NR	6.3	NR
	Weight Watchers	11		100			35	1.2[‖]	NR		NR
	Individualized group	13		100			35.5	1.1[‖]	NR		NR
	Comprehensive group	11		100			36.8	1[‖]	NR		NR
Duncan 2016[51]	Control	162	159	42.8	54.8	8.48	31.8	6.91	NR	NR	NR
	Intervention	158	154	45.1	53.1	9.83	33.8	7.14	NR	NR	NR
Eakin 2014[52]	Usual care	151	151	43.0	58.3	9.0	33.2	6.0	74.8	100	NR
	Telephone intervention	151	151	44.4	57.7	8.1	33.1	6.3	84.1	100	NR
Fernandez-Ruiz 2018[53]	Control	37	37	51.4	62.8	8.9	34.3	4.5	NR	62.2	86.5
	Intervention (healthy eating, exercise & CBT)	37	37	48.6	59.4	9.1	32.4	3.8	NR	43.2	78.4
Fisher 2011[54]	Diet only	29	NR	100	NR	NR	28	3	NR	NR	NR
	Diet + aerobic training	43	NR		NR	NR			NR	NR	NR
	Diet + resistance training	54	NR		NR	NR			NR	NR	NR
Foley 2016[55]	Usual care (Control)	175	175	68	50.5	8.7	35.9	3.7	NR	3.4	29.1
	Weight loss intervention	176	176	68	50.9	9.1	35.9	4.1	NR	3.4	29.5
Foster-Schubert 2012[56]	Control- usual care	87	87	100	57.4	4.4	30.7	3.9	NR	NR	NR
	Calorie reduced diet	118	118	100	58.1	5.9	31	3.9	NR	NR	NR
	Aerobic exercise (N/A)	117	117	N/A	N/A	N/A	N/A	N/A	N/A	N/A	N/A
	Intervention - diet and exercise	117	117	100	58.0	4.5	31	4.3	NR	NR	NR
Fuller 2012[57]	Western diet group	35	35	54.3	47.1	11.1	31.0	3.8	NR	NR	NR
	Korean diet group	35	35	71.4	43.7	11	31.2	4.0	NR	NR	NR
Green 2015[58]	Usual care	96	96	71.9	48.3	9.7	38.2	7.3	NR	16.7	30.2
	STRIDE	104	104	72.1	46.2	11.4	38.3	9.1	NR	13.5	28.8
Hageman 2017[59]	Web-based only	101	101	100	53.9	6.9	NR	NR	3.0	3.0	NR

Study ID Ref No.*	Groups:	Randomised	Number of participants reported at baseline	Gender (%F)	Age		BMI		Comorbidities at baseline (%)†		
					Mean	*SD*	*Mean*	*SD*	*CV morbidity*	*Type II DM*	*Hypertension*
	Web-based discussion	100	100				NR	NR	1.0	5.0	NR
	Web-based email	100	100				NR	NR	4.0	6.0	NR
Hardcastle 2013[60]	Control	131	131		50.41	10.87	33.37	4.47	NR	NR	18.3
	MI counselling intervention	203	203		50.1	10.54	33.66	5.12	NR	NR	22.7
Harrigan 2016[61]	Usual Care Group	33	33	100	58	7.5	34	7.5	NR	NR	NR
	Telephone Weight Loss Counseling	34	34	100	60	7.7	31.8	5.4	NR	NR	NR
	In-Person Weight Loss Counseling	33	33	100	58.9	7.3	33.5	6.7	NR	NR	NR
Hunt 2014[62]	Control, Wait-list	373	373	0	47.2	7.89	35.1	4.8	NR	NR	NR
	FFIT	374	374	0	47	8.07	35.5	5.1	NR	NR	NR
Irwin 2003[63]	Control Group	86	86	100	60.6	59.1, 62.1**	30.6	29.8, 31.4**	NR	NR	NR
	Exercise group	87	87		61	59.6, 62.5**	30.5	29.6, 31.4**	NR	NR	NR
Jebb 2011[64]	Standard care	395	395	86	48.2	12.2	31.3	2.6	NR	6.8	25.1
	Commercial programme	377	377	88	46.5	13.5	31.5	2.6	NR	6.4	25.5
Jebb 2017[65]	Usual care	140	140	60.0	47.4	12.8	36.8	5.1	NR	14.3	21.4
	Low energy total diet replacement programme	138	138	60.5	48.2	11.5	37.6	5.7	NR	15.2	23.9
Jenkins 2017[66]	Control	486	486	79.4	44.9	43.8, 46.0**	32.5	32.0, 33.0**	5.8	NR	8.6
	Dietary advice only	145	145	75.9	46.2	44.0, 48.4**	31.7	30.8, 32.7**	7.6	NR	9.0
	Food basket only	148	148	72.3	44.9	43.1, 46.7**	32.6	31.6, 33.5**	7.4	NR	10.1
	Food and advice	140	140	76.4	42.4	40.4, 44.4**	32.7	31.7, 33.7**	5.0	NR	5.7
Katula 2013[67]	Enhanced Usual Care Comparison Condition	150	150	57.3	58.5	9.0	32.6	4.1	NR	0.0	52.0
	Lifestyle Weight-Loss Intervention	151	151	57.6	57.3	10.1	32.8	3.9	NR	0.0	51.7
Katzer 2008[68]	Mail-delivered 'non-dieting' program (P3)	101	225	100	46.1	8.9	35.4	5.7	NR	NR	NR
	Group 'non-dieting' program (P2)	62							NR	NR	NR
	Group 'non-dieting' program plus Relaxation (P1)	62							NR	NR	NR
King 1989[69]	Control (N/A)	52	N/A	N/A	N/A	N/A	N/A	N/A	N/A	N/A	N/A
	Exercise only	52	36	0	44.8	7.4	NR	NR	NR	NR	NR
	Diet only	51	36	0	45	7.6	NR	NR	NR	NR	NR
Knauper 2018[70]	Standard DPP	101	85	76.5	49.4	11.8	NR	NR	NR	NR	NR
	Enhanced DPP	107	87	83.9	50.9	12.1	NR	NR	NR	NR	NR
Diabetes Prevention Program R G 2009[71]	Placebo	1082	1082	69	50.3	10.4	32.2	6.7	NR	All at high risk of T2DM	30.0
	Metformin (N/A)	1073	1073	N/A	N/A	N/A	N/A	N/A	N/A		
	Lifestyle	1079	1079	68	50.6	11.3	33.9	6.8	NR		

Study ID [Ref No.*]	Groups:	Randomised	Number of participants reported at baseline	Gender (%F)	Age		BMI		Comorbidities at baseline (%)[†]		
					Mean	*SD*	*Mean*	*SD*	*CV morbidity*	*Type II DM*	*Hypertension*
Kuller 2012[72]	Control - health education	255	255	100	57	NR	30.9	3.8	NR	NR	NR
	Intervention - lifestyle change	253	253	100	56	NR	30.6	3.8	NR	NR	NR
Kumanyika 2012[73]	Basic programme	137	137	82.5	46.8	11.6	37.3	6.4	10.9	19.7[‡]	41.6
	Basic plus programme	124	124	86.3	47.6	11.9	37.2	6.5	6.5	16.9[‡]	46.0
Ley 2004[74]	Control diet	70	70	20	52	0.8[‖]	29.1	0.6[‖]	NR	NR	NR
	Reduced-fat	66	66	31.8	52.5	0.8[‖]	29.3	0.6[‖]	NR	NR	NR
Li 2016[75]	Usual care group	60	60	35	59	3.9	25.2	0.9	NR	100	NR
	Diet group	79	79	46.8	59.7	6.5	27.2	2.8	NR	100	NR
	50g-oats group	80	80	48.8	59.7	6.1	26.9	2.7	NR	100	NR
	100g-oats group	79	79	58.2	59.4	6.8	27.4	2.4	NR	100	NR
Li 2005[76]	Individualized diet plan	52	36	33.3	56.6	10.4[‖]	33.7	3.6[‖]	NR	100	NR
	Soy-based meal replacement	52	46	41.3	54.4	9.3[‖]	32.8	3.7[‖]	NR	100	NR
Lindstrom 2003[77]	Control	257	257	68.5	55	7	31.4	4.5	NR	NR	31.1
	Intervention	265	265	65.7	55	7	31.1	4.5	NR	NR	29.1
Liss 2016[78]	Standard care arm	167	167	48.5	56.6	12.2	34.9	7.3	NR	100	76.6
	Standard care plus group-based lifestyle intervention	164	164	51.8	57.1	10.6	36.2	7.8	NR	100	80.5
Little 2016[79]	Control, Nurse follow-up	279	279	66	52.7	13.3	37.1	6.0	NR	NR	NR
	Web-based support with minimal support (Remote)	270	270	60	54.7	13.0	36.3	5.7	NR	NR	NR
	Web-based + nurse support (face to face)	269	269	65	53.7	13.2	36.7	5.4	NR	NR	NR
Manning 1994[80]	Clinic visit	37	37	56.8	57.3	54.1, 60.5[**]	31.2	30.1, 32.3[**]	NR	NR	NR
	Behavioural	38	38	47.4	58.8	55.9, 61.7[**]	32.2	30.5, 33.9[**]	NR	NR	NR
	Home visits	35	35	42.7	55.2	51.6, 58.8[**]	32	30.9, 33.1[**]	NR	NR	NR
	Dexfenfluramine (N/A)	N/A	N/A	N/A	N/A	N/A	N/A	N/A	N/A	N/A	N/A
	Routine usual care (N/A)	N/A	N/A	N/A	N/A	N/A	N/A	N/A	N/A	N/A	N/A
Mefferd 2007[81]	Control	29	29	100	56.4	7.5	31.3	4.8	NR	NR	NR
	Intervention	56	56	100	55.9	8.7	31	3.7	NR	NR	NR
Melchart 2017[82]	Control group	57	55	72.7	52.1	10	31.5	2	NR	0.0	NR
	Intervention group	109	111	74.8	49.9	9.7	31.8	2	NR	0.0	NR
Melin 2003[83]	Control, less intensively treated	21	21	90.7	39.4	26-57[#]	35.2	4.6	NR	14.3	NR
	Intensively treated	22	22		40.7	25-60[#]	35.6	4.5	NR	NR	NR
Menard 2005[84]	Control - usual care	36	36	38.9	55.9	8.6	32.6	5.7	NR	100	NR
	Intervention - intensive multitherapy	36	36	25	53.7	7.5	32.9	5.5	NR	100	NR
Mengham 1999[85]	Control	NR	36	44.4	63.5	10.9	31.7	4.9	NR	89.2	NR
	Intervention	NR	38	44.7	57.8	13.5	31.4	4.4	NR		NR
Mensinger 2016[86]	Control, Weight Neutral Program	40	40	100	39.8	4.34	37.4	0.6	NR	NR	NR
	Weight Loss Program	40	40	100	39.4	3.91	38.6	0.7	NR	NR	NR

Study ID [Ref No.*]	Groups:	Randomised	Number of participants reported at baseline	Gender (%F)	Age		BMI		Comorbidities at baseline (%)[†]		
					Mean	*SD*	*Mean*	*SD*	*CV morbidity*	*Type II DM*	*Hypertension*
Mitsui 2008[87]	Control	22	21	54.5	67.4	10.6	25.6	2.5	NR	NR	18.6
	Intervention	24	22	54.2	64	8.9	24.8	2.2	NR	NR	
Moreno 2014[88]	Low-calorie diet	39	26	96.1	46.3	9.3	35.1	5.3	NR	3.8	19.2
	Very low-calorie-ketogenic diet	40	27	81.4	44.4	8.6	35.1	4.5	NR	7.4	14.8
Morgan 2010[89]	Control (Information and self-help)	31	31	0	34	11.6	30.5	3.0	NR	NR	NR
	SHED-IT (Internet) group	34	34	0	37.5	10.4	30.6	2.7	NR	NR	NR
Muggia 2014[90]	Standard care group	83	83	71.1	43.5	10.0	32.5	3.7	NR	NR	NR
	Brief CBT group	80	80	76.3	46.2	11.7	31.9	3.	NR	NR	NR
Nakata 2014[91]	Control (N/A)	63	63	N/A	N/A	N/A	N/A	N/A	N/A	N/A	N/A
	Education-only	62	62	66	51.7	6.8	29.2	3.8	NR	0.0	29.0
	Group-based support	63	63	81	50.7	6.7	29	3.0	NR	0.0	17.5
Nanchahal 2012[92]	Usual care control	190	190	72.6	49.4	14.1	33.0	5.4	NR	12.3	NR
	CAMWEL Intervention	191	191	71.7	48.2	15.5	33.9	5.6	NR		NR
Ng 2015[93]	Control group	43	43	30.7	52	9.3	30.5	4.2	NR	25.6[‡]	20.9
	Lifestyle modification program	61	61	21.3	51.4	9.1	30.2	3.9	NR	23.0[‡]	26.2
Nilsen 2011[94]	Control, Individual Physician Group	104	104	47	45.9	11	35.9	6	NR	NR	NR
	Individual Plus Interdisciplinary Group	109	109	53	47	11	37.6	6	NR	NR	NR
Nordby 2012[95]	Control	15	12	0	31	7	28	1.5	NR	0.0	0.0
	Training and increased diet (N/A)	13	N/A	N/A	N/A	N/A	N/A	N/A	N/A	N/A	N/A
	Training	17	12	0	28	5	28.3	1.1	NR	0.0	0.0
	Energy-reduced diet	15	12	0	32	7	28	1.3	NR	0.0	0.0
Oldroyd 2006[96]	Control group	39	32	31.25	57.5	41 – 73[#]	29.9	4.9	NR	NR	NR
	Intervention group	39	37	54.05	58.2	41 – 75[#]	30.4	5.6	NR	NR	NR
Pan 1997[97]	Control	138	138	43	46.6	9.3	26.2	3.8	NR	0.0	NR
	Intervention group (Exercise: n=155; Diet: n = 148; Diet plus exercise: n = 135)	438	438	47	44.7	9.3	25.6	4.0	NR	0.0	NR
Parikh 2010[98]	Control	49	49	84	50	18	31	5.0	NR	0.0	NR
	Intervention	50	50	86	46	15	32	4.0	NR	0.0	NR
Pedersen 2013[99]	Aerobic interval training	35	26	15	62.3	5.7	31.6[§]	29.6, 34.8[§]	26	0	96.4
	Low energy diet	35	29	28	63.8	6.8	31.1[§]	29.9, 32.7[§]	29	0	
Pettman 2009[100]	Control	50	50	72	NR	NR	36.5	6.5	NR	NR	NR
	Intervention B - Passive follow-up	54	NR	NR	NR	NR	37.3	6.2	NR	NR	NR
	Intervention A - Active follow-up	49	NR	NR	NR	NR	36.1	6.6	NR	NR	NR
Promrat 2010[101]	Control	10	10	20	47.6	12.0	33.7	4.7	NR	40.0	NR
	Lifestyle Intervention	21	21	33.3	48.9	10.9	33.9	5.3	NR	52.4	NR
Provencher 2009[102]	Control group	48	47	100	41.8	6.0	30.5	3.0	NR	0.0	NR
	Social support	48	46	100	42.3	5.5	30.6	3.1	NR	0.0	NR
	Health-At-Every-Size	48	48	100	42.8	5.5	30.1	3.0	NR	0.0	NR

Study ID [Ref No.*]	Groups:	Randomised	Number of participants reported at baseline	Gender (%F)	Age		BMI		Comorbidities at baseline (%)[†]		
					Mean	*SD*	*Mean*	*SD*	*CV morbidity*	*Type II DM*	*Hypertension*
Ridgeway 1999[103]	Control	28	20	75	65	NR	NR	NR	NR	100	NR
	Intervention Group	28	18	61	62	NR	NR	NR	NR	100	NR
Rock 2015[104]	Control	349	348	100	56.5	9.5	31.4	4.6	NR	NR	28.7
	Intervention	348	345	100	56.1	9.4	31.6	4.7	NR	NR	30.7
Rolls 2005[105]	Comparison-control	50	50	NR	45.2	1.2[‖]	31.3	0.4[‖]	NR	NR	NR
	Two snacks	50	50	NR	44.5	1.2[‖]	31.4	0.4[‖]	NR	NR	NR
	One soup	50	50	NR	45.1	1.2[‖]	30.9	0.5[‖]	NR	NR	NR
	Two soups	50	50	NR	43.8	1.2[‖]	30.8	0.5[‖]	NR	NR	NR
Rolls 2017[106]	Standard advice	62	62	100	49.5	12.0	34.1	4.3	NR	NR	NR
	Pre-portioned foods group	62	62	100	50.1	10.1	34.2	4.1	NR	NR	NR
	Portion selection group	62	62	100	50.4	9.6	33.6	4.2	NR	NR	NR
Rosas 2015[107]	Usual care	41	41	78	47.6	10.5	34.9	4.4	NR	43.9[‡]	NR
	Case-management intervention	84	84	76.2	47.9	11.9	36	5.7	NR	44.0[‡]	NR
	Case-management + Community health worker intervention	82	82	76.8	46	10.7	35.5	5.1	NR	41.5[‡]	NR
Ross 2012[108]	Control condition	241	241	70.1	52.4	11.8	32	4.2	NR	NR	33.2
	Behavioral intervention group	249	249	70.28	51.3	11	32.6	4.1	NR	NR	25.7
Samaras 1997[109]	Control	13	13	53.8	60.5	2.1[‖]	35.7	1.6[‖]	NR	NR	NR
	Intervention	13	13	69.2	60.5	7.8[‖]	32.3	1.1[‖]	NR	NR	NR
Sattin 2016[110]	Health Education intervention	287	287	82.6	46.4	10.9	35.6	7.6	NR	0	NR
	Fit body and soul intervention	317	317	84.2	46.6	10.9	35.8	7	NR	0	NR
Schubel 2016[111]	Control group	52	52	52	50.7	7.1	31.1	3.6	NR	0	NR
	Continuous Calorie Restriction	49	49	49	50.5	8.0	31.2	4.0	NR	0	NR
	Intermittent Calorie Restriction	49	49	49	49.4	9.0	32	3.8	NR	0	NR
Seligman 2011[112]	Standard-of-care strategy	25	25	32	42	8[‖]	34.7	0.6[‖]	NR	NR	52.0
	Healthy diet and step counter	25	25	36	44	7[‖]	34.4	0.6[‖]	NR	NR	64.0
	Healthy diet and fitness	26	25	36	43	8[‖]	35.2	0.5[‖]	NR	NR	64.0
Shikany 2013[113]	Food-based diet	60	60	90	39.7	9.1	41.3	3.8	NR	NR	NR
	Meal replacement	60	60	86.7	40.2	9.2	40.6	3.8	NR	NR	NR
Snel 2012[114]	VLCD only	14	14	38.5	56	2	37.9	1.4	NR	100	NR
	VLCD + exercise	13	13	57.1	53	3	36.4	1.1	NR	100	NR
Stevens 1993[115]	Control	256	256	37	42.4	6.2	29.5	2.8	NR	NR	0.0
	Intervention	308	308	17	43.1	6.0	29.5	2.9	NR	NR	0.0
Stevens 2001[116]	Control	596	596	31.7	43.2	6.1	30.9	3.2	NR	NR	NR
	Intervention	595	595	37	43.4	6.1	31	3.3	NR	NR	NR
	Sodium only intervention (N/A)	594	N/A	N/A	N/A	N/A	N/A	N/A	N/A	N/A	N/A
	Combined intervention (N/A)	597	N/A	N/A	N/A	N/A	N/A	N/A	N/A	N/A	N/A
Sundfor 2018[117]	Continuous energy restriction	58	58	51.7	47.5	11.6	35.3	3.5	NR	6.9[#]	92.0
	Intermittent energy restriction	54	54	48.1	49.9	10.1	35.1	3.9	NR	1.9[#]	37.0
Tapsell 2017[118]	Usual care (Control)	126	126	73	43.8	7.46	32.49	4.12	NR	NR	11.1

Study ID Ref No.*	Groups:	Randomised	Number of participants reported at baseline	Gender (%F)	Age		BMI		Comorbidities at baseline (%)†		
					Mean	*SD*	*Mean*	*SD*	*CV morbidity*	*Type II DM*	*Hypertension*
	Intervention Group	125	124	73	43.79	7.97	32.59	4.25	NR	NR	16.1
	Intervention plus food supplement group (N/A)	126	N/A	N/A	N/A	N/A	N/A	N/A	N/A	N/A	N/A
TarragaMarcos 2017[119]	G3	55	55	32.4	49.8	6.6	30.7	3.4	NR	NR	NR
	G2	61	61	34.3	49.7	6.4	30.8	3.6	NR	NR	NR
	G1	60	60	33.3	50.1	7.2	30.3	3.2	NR	NR	NR
Teeriniemi 2018[120]	Control	89	89	51.7	46.5	10.2	30.5	2.3	NR	2.2‡	25.8
	SHG Counselling	87	87	48.3	44.4	10.2	30.7	2.2	NR	2.3‡	26.4
	CBT Counselling	85	85	49.4	46.4	9.7	30.5	1.9	NR	3.5‡	20.0
	Control plus HBCSS	91	91	47.3	47	9.4	30.3	2.0	NR	3.3‡	22.0
	SHG Counselling plus HBCSS	92	92	48.9	46.4	10.5	30.4	2.1	NR	1.1‡	25.0
	CBT Counselling plus HBCSS	88	88	50	44.8	9.6	30.3	2.1	NR	2.3‡	15.9
ter Bogt 2009[121]	GP usual care	232	232	53.9	56.9	7.8	29.6	3.6	NR	NR	62.5
	Lifestyle counselling from NP	225	225	49.8	55.3	7.7	29.5	3.1	NR	NR	60.9
Tsai 2010[122]	Control	26	26	88	47.6	12.7	37.6	5.6	NR	NR	NR
	Brief counselling	24	24		51.3	11.3	35.4	5.9	NR	NR	NR
Tuomilehto 2009[123]	Control	41	41	27	50.9	8.6	31.4	2.7	NR	7.3	36.6
	Intervention	40	40	25.7	51.8	9.0	33.4	2.8	NR	10	45.0
van de Glind 2017[124]	Comparison group	553	553	0.0	45.6	8.7	33.4	4.7	NR	NR	NR
	EuroFIT group	560	560	0.0	45.9	9.0	33.1	4.6	NR	NR	NR
vanWier 2011[125]	Control – Brochure	460	460	33.5	43	8.7	29.6	3.7	2.0	2.0‡	10.0
	Internet Group	464	464	34.9	43	8.4	29.6	3.4			
	Phone Group	462	462	30.5	43	8.8	29.5	3.5			
Vissers 2010[126]	Control	21	21	74.7	44.8	11.4	30.8	3.4	NR	NR	NR
	Diet only group (Diet)	20	20		45.5	13.1	32.9	3.1	NR	NR	NR
	Diet + fitness training group (Fitness)	20	20		44.7	13	33.1	3.4	NR	NR	NR
	Diet + WBV group (Vibration)	18	18		43.3	9.6	31.9	4.7	NR	NR	NR
Volpe 2008[127]	Exercise only	34	34	50	43.5	7.5	30.5	3.1	NR	NR	NR
	Diet only	28	28	53.6	44.0	6.2	30.9	2.8	NR	NR	NR
	Combination of diet and exercise	28	28	50	45.7	7.4	30.5	2.6	NR	NR	NR
Weinstock 2013[128]	Conference Call DPP	128	128	71.9	52.7	12.8	39.7	8.3	NR	0.0	66.1
	Individual Call DPP	129	129	78.3	50.7	13.1	38.9	7.6	NR		
West 2007[129]	Attention control	108	108	NR	52	10	36.5	5.4	NR	100.0	NR
	Motivational interviewing	109	109	NR	54	10	36.5	5.5	NR	100.0	NR
The Look AHEAD	Diabetes support and education	2575	2575	59.6	58.9	6.9	36	5.8	NR	100.0	84.0

Study ID [Ref No.*]	Groups:	Randomised	Number of participants reported at baseline	Gender (%F)	Age		BMI		Comorbidities at baseline (%)[†]		
					Mean	*SD*	*Mean*	*SD*	*CV morbidity*	*Type II DM*	*Hypertension*
Research Group 2010[130]											
	Intensive lifestyle intervention	2570	2570	59.3	58.6	6.8	35.9	6.0	NR	100.0	84.5
Wing 1988[131]	Diet plus placebo exercise	13	13	84	52.5	8.9	37.5	6.2	NR	100.0	NR
	Diet plus moderate exercise	12	12		56.2	7.5	38.1	6.4	NR	100.0	NR
Wing 1988b[131]	Diet only	15	15	70.0	55.1	7.2	37.9	6.5	NR	100.0	NR
	Diet plus exercise	15	15		56.1	6.4	38.2	6.6	NR		NR
Wing 1991[132]	Behavior therapy alone	19	16	75.0	51.9	9.9	38.1	5.7	NR	100.0	NR
	Behavior therapy plus VLCD	17	17	76.4	50.6	7.7	37.3	4.7	NR	100.0	NR
Wing 1998[133]	Control	40	40	80	45.3	4.9	36.0	5.4	NR	0.0	NR
	Diet	37	37	78	45.0	4.7	36.1	4.1	NR	0.0	NR
	Exercise	37	37	81	46.4	4.5	36	3.7	NR	0.0	NR
	Diet plus exercise	40	40	77	46.3	3.8	35.7	4.1	NR	0.0	NR
Yannakoulia 2008[134]	Usual care group	15	15	53.3	56.9	10	31.6	5	NR	100	NR
	Intensive care group	15	15	40.0	56.3	8.8	32.2	4.1	NR	100	NR
Yates 2009[135]	Control group	34	29	41	65	10	29.8	4.4	NR	NR	NR
	PREPARE group	31	29	31	64	7	29.5	4.9	NR	NR	NR
	PREPARE with pedometer	33	29	31	66	8	28.7	4.8	NR	NR	NR
Yeh 2016[136]	Control group	30	30	50	60.9	12.2	25.8	2.3	NR	NR	NR
	Intervention group	30	30	63.3	56.8	9.5	26.3	2.4	NR	NR	NR
Yin 2018[137]	Comparison-Control Group	75	75	100	53.27	7.17	27.43	2.75	NR	NR	24.0
	Intervention Group	109	109	100	51.06	7.15	27.42	2.91	NR	NR	27.5
Zhang 2016[138]	Control	74	74	62.2	54	6.8	28	2.7	NR	NR	NR
	Moderate exercise	73	73	69.9	54.4	7.4	28.1	3.3	NR	NR	NR
	Vigorous-moderate exercise	73	73	71.2	53.2	7.1	27.9	2.7	NR	NR	NR

*Ref No. Reference number in main paper.
†Comorbidity definitions varied for each study;
‡Unclear whether DM percentage listed includes Type II and Type I;
§ Median (IQR); ‖ Standard error; # Range; ** 95% Confidence intervals

CBT: Cognitive Behaviour Therapy; **CV** = Cardiovascular; **DM:** Diabetes Mellitus; **DPP:** Diabetes Prevention Program**; N/A** = Not applicable; **NR** = Not reported; **VLCD:** Very low calorie diet

Table S6. Intervention characteristics

*Ref No. Reference number in main paper. †See table footnotes for Provider category descriptions ‡Unless otherwise stated; §Exercise sessions were assumed to be unsupervised and did not contribute to the number of sessions unless otherwise stated.

Approx.: Approximately; **Appt.**: Appointment/s; **Fin. Incentives;** Financial Incentives**; GP:** General Practitioner **Inter. Fasting:** Intermittent Fasting; **Min/s:** Minute/s **M/Mths:** month/s**; MR – F =** Meal replacement (Full); **MR – P =** Meal replacement (Partial); **N:** Number; **N/A:** Not applicable; **NR:** Nor reported; **Nutrition Edu.** = Nutrition Education; **PA:** Physical Activity**; SMS**: Short Message Service; **VLCD:** Very low-calorie diet

Study ID Ref No.*	Groups:	Intervention type	Intervention faded in intensity	Features (meal replacements, nutrition education, financial incentives, intermittent fasting, content designed to help participants following programme end)	Provider †	Provider training received	Delivery		Intervention setting	Intervention timing (months)		Sessions			Intervention personalised, titrated or adapted
							Mode	Format		Last contact	End (step change in intensity)	N§	Frequency	Length per session with description for varying lengths. (minutes ‡)	
Abed 2013[16]	Control	Control	No	Nutrition Edu.; Help following programme end		No			Health Care			0			No
	Weight Management	Diet and exercise	Yes	MR-P; Nutrition Edu.; Help following programme end	Physician	No	Individual	Face to Face; Print	Health Care	15	2	5	Every 3 months	20 - 40 mins; Additional goal-directed face-to-face clinic visits were scheduled as required; 8 weeks VLCD. 3m to 15m low GI meals. Exercise plan of increasing intensity. Exercise 3 per week for 15 months; 24-hour e-mail and telephone support provided as required.	Yes
Ackermann 2011[17]	Standard advice alone (controls)	Diet and exercise	No	Nutrition Edu.			Individual	Face to Face	Community	0	0	1	Once	5	Yes
	YMCA DPP intervention	Diet and exercise	No	Nutrition Edu.			Individual and Group	Face to Face	Community	5	5	16	Weekly	60 – 90	Yes
Ahern 2017[18]	Brief intervention	Control	No	Nutrition Edu.	Other	Unclear	Individual	Face to Face; Print	Community	1	1	1	Once		No

Study ID [Ref No.*]	Groups:	Intervention type	Intervention faded in intensity	Features (meal replacements, nutrition education, financial incentives, intermittent fasting, content designed to help participants following programme end)	Provider [†]	Provider training received	Delivery		Intervention setting	Intervention timing (months)		Sessions			Intervention personalised, titrated or adapted
							Mode	Format		Last contact	End (step change in intensity)	N[§]	Frequency	Length per session with description for varying lengths. (minutes [‡])	
	12-week behavioural weight-loss programme	Diet and exercise	No	Nutrition Edu.	Health Trainer	Yes	Group	Face to Face; Internet	Community	12	12	12	Weekly	30	No
	52-week behavioural weight-loss programme	Diet and exercise	No	Nutrition Edu.	Health Trainer	Yes	Group	Face to Face; Internet	Community	52	52	52	Weekly	30	No
Almanza - Aguilera 2018[19]	Control (general recommendations)	Control	No	Nutrition Edu.	Nutritionist	No	Unclear	Face to Face; Other		12		2	3m, 12m		No
	Treatment (lifestyle weight loss intervention)	Diet and exercise	No	Nutrition Edu.	Nutritionist	No	Unclear	Face to Face; Other		12	3	13	Weekly (0-3m); Once at 12m		No
Andersen 1999[20]	Diet + Lifestyle Activity	Diet and exercise	No	Nutrition Edu.; Help following programme end	Psychologist/ Counsellor	No	Group	Face to Face; Other	Community	17	4	16	Weekly	Cognitive behavioural sessions = 60 mins. Advised 30 mins moderate physical activity on most days per week.	No
	Diet + Aerobic Group	Diet and exercise	No	Nutrition Edu.; Help following programme end	Other	No	Group	Face to Face	Community	17	4	64	Weekly cognitive behavioural session + aerobic session 3 x per week	Cognitive behavioural sessions = 60 mins. Aerobics classes: 5-10 min warm up, 15-45 mins aerobic phase (increased by 4 mins per week), 5 min cool-down.	Yes
Anderson 2014[21]	Control (weight loss booklet only)	Control	No	Nutrition Edu.; Help following programme end		No	Individual	Print	Home	12	12	0			No

Study ID [Ref No.*]	Groups:	Intervention type	Intervention faded in intensity	Features (meal replacements, nutrition education, financial incentives, intermittent fasting, content designed to help participants following programme end)	Provider †	Provider training received	Delivery		Intervention setting	Intervention timing (months)		Sessions			Intervention personalised, titrated or adapted
							Mode	Format		Last contact	End (step change in intensity)	N§	Frequency	Length per session with description for varying lengths. (minutes ‡)	
	Intervention (BeWEL)	Diet and exercise	Yes	Nutrition Edu.; Help following programme end	Psychologist/ Counsellor	No	Individual	Face to Face; Telephone; Print	Health Care; Home	3	12	12	Monthly	The 3 counsellor sessions were each 1 hour. The 9 phone calls were each 15 minutes.	Yes
Appel 2011[22]	Control (Self-directed)	Control	No	Nutrition Edu.	Health Trainer	Unclear	Individual	Face to Face; Print	Health Care			1	0, 24m		
	Remote Support Only (N/A)	N/A	N/A	N/A	N/A	N/A	N/A	N/A	N/A	N/A	N/A	N/A	N/A	N/A	N/A
	In-Person Support	Diet and exercise	Yes	Nutrition Edu.	Other AHPs; Health Trainer	Yes	Individual and Group	Face to Face; Telephone; Internet; Other	Health Care	24	6	57	Weekly (0-3m); Three monthly contacts over the next 3 months; Two monthly contacts for the remainder of the study.	Individual sessions approx. 20 mins; In-person group sessions: 90 mins	Yes
Ard 2018[23]	Exercise Only	Exercise only	No			Unclear	Group	Face to Face; Print	Community	12	6	38	Weekly 0-24 weeks and then biweekly until 12m	1 hour	No
	Exercise + Diet Quality + Weight Maintenance	Diet and exercise	No	Nutrition Edu.		Unclear	Group	Face to Face; Print;	Community	12	6	38	Weekly 0-24 weeks and then biweekly until 12m	1 hour	No
	Exercise + Diet Quality + Weight Loss	Diet and exercise	No	Nutrition Edu.		Unclear	Group	Face to Face; Print	Community	12	6	38	Weekly 0-24 weeks and then biweekly until 12 m	1 hour	No
Ard 2004[24]	“advice only” comparison group	Diet and exercise	No	Nutrition Edu.	Dietitian	No	Individual	Face to Face; Print;	Health Care	6	6	2		30	Yes
	“established” behavioural intervention group	Diet and exercise	Yes	Nutrition Edu.; Help following programme end			Individual and Group	Face to Face	Health Care	18	6	23	Weekly (3m), Biweekly (3m), Monthly (12m)		Yes
	Established + DASH Intervention	Diet and exercise	Yes	Nutrition Edu.; Help following programme end			Individual and Group	Face to Face	Health Care	18	6	23	Weekly (3m), Biweekly (3m), Monthly (12m)		Yes

Study ID [Ref No.*]	Groups:	Intervention type	Intervention faded in intensity	Features (meal replacements, nutrition education, financial incentives, intermittent fasting, content designed to help participants following programme end)	Provider †	Provider training received	Delivery		Intervention setting	Intervention timing (months)		Sessions			Intervention personalised, titrated or adapted
							Mode	Format		Last contact	End (step change in intensity)	N§	Frequency	Length per session with description for varying lengths. (minutes ‡)	
	Group														
Ashley 2001[25]	Control, Diet	Diet and exercise	Yes	Nutrition Edu.; Help following programme end	Dietitian	Unclear	Group	Face to Face; Print	Community	24	12	37	Year 1: weekly for 3m, biweekly for 3m, monthly for 6m. Year 2: monthly	60	No
	MR - Physician/Nurse led	Diet and exercise	Yes	MR-P; Nutrition Edu.; Help following programme end	Nurse (General); Physician	Unclear	Group	Face to Face; Print	Community	24	12	37	Year 1: every other week. Year 2: monthly	15	Yes
	MR - Dietician lead	Diet and exercise	Yes	MR-P; Nutrition Edu.; Help following programme end	Dietitian	Unclear	Group	Face to Face; Print	Community	24	12	37	Year 1: weekly for 3m, biweekly for 3m, monthly for 6m. Year 2: monthly	60	Yes
Azar 2013[26]	Control, Usual care	Control	No				Unclear	Face to Face	Community			4			No
	Self-directed	Diet and exercise	Yes	Nutrition Edu.	Dietitian		Individual and Group	Face to Face; Internet; Other	Home	15	3	40			Yes
	Coach-led	Diet and exercise	Yes	Nutrition Edu.	Psychologist/ Counsellor; Dietitian		Individual and Group	Face to Face	Community	15	3	52	Weekly	90 – 120	Yes
Bacon 2002[27]	Health at Every Size - control	Diet and exercise	No	Nutrition Edu.	Psychologist/ Counsellor		Group	Face to Face	Community	12	6	30	Weekly for first 6m, then monthly	1.5 hour	No
	Diet Group - intervention	Diet and exercise	No	Nutrition Edu.	Dietitian		Group	Face to Face	Community	12	6	30	Weekly for first 6m, then monthly	1.5 hour	No
Barnes 2017[28]	Treatment as usual (N/A)	Control	N/A	N/A	N/A	N/A	N/A	N/A	N/A	N/A	N/A	N/A	N/A	N/A	N/A
	Nutrition - ATTENTION CONTROL	Diet only	No	Nutrition Edu.	Other AHPs		Individual	Face to Face Internet; Print	Community	3	3	5	Every 3 weeks	60 mins the first session and 20 mins the rest (4 sessions)	
	Motivational interviewing	Diet and exercise	No	Nutrition Edu.			Individual	Face to Face; Internet; Print	Community	3	3	5	Every 3 weeks	60 mins the first session and 20 mins the rest (4 sessions)	
Bartels	Control,	Exercise	No			No	Individual	Face to	Community			1			No

Study ID [Ref No.*]	Groups:	Intervention type	Intervention faded in intensity	Features (meal replacements, nutrition education, financial incentives, intermittent fasting, content designed to help participants following programme end)	Provider [†]	Provider training received	Delivery		Intervention setting	Intervention timing (months)		Sessions			Intervention personalised, titrated or adapted
							Mode	Format		Last contact	End (step change in intensity)	N[§]	Frequency	Length per session with description for varying lengths. (minutes [‡])	
2015[29]	Fitness club membership	only						Face							
	IN SHAPE	Diet and exercise	No	Nutrition Edu.	Health Trainer; Personal Trainer	Yes	Individual	Face to Face	Community	12	12	52	Weekly	45 – 60	Yes
Beeken 2017[30]	Usual care	Diet and exercise	No	Nutrition Edu.	Health care professional (not specified)	No	Individual and Group	Other	Health Care; Community	Varied (12 weeks, min. 2 appts, 12 weekly sessions, monthly appts)	varied	Varied			Yes
	10TT	Diet and exercise	No	Nutrition Edu.; Help following programme end	Nurse (General)	Yes	Unclear	Face to Face; Telephone; Print	Health Care	3	3	1	Single session	30	No
Bennett 2013[31]	Control, usual care	Control	No			No				12					No
	Weight gain prevention intervention	Diet and exercise	No	Nutrition Edu.		Yes	Individual	Telephone;	Community; Home	12	12	64	Weekly (52) and monthly (12)	10 mins (52 weekly IVR (interactive voice response calls)) 12 monthly 20 min calls	Yes
Bennett 2012[32]	Control, Usual care	Diet only	No			No	Other – Print only	Print							No
	Be Fit, Be Well	Diet and exercise	Yes	Nutrition Edu.	Health care professional (not specified)	Yes	Individual and Group	Face to Face; Telephone; Internet	Community; Home	24	12	30	Monthly for the first year and bimonthly for the second year. Additional 12 optional monthly group sessions	15-20 mins; Telephone counselling sessions were held monthly for the first year and bimonthly for the second year. There were an	Yes

Study ID Ref No.*	Groups:	Intervention type	Intervention faded in intensity	Features (meal replacements, nutrition education, financial incentives, intermittent fasting, content designed to help participants following programme end)	Provider [†]	Provider training received	Delivery		Intervention setting	Intervention timing (months)		Sessions			Intervention personalised, titrated or adapted
							Mode	Format		Last contact	End (step change in intensity)	N[§]	Frequency	Length per session with description for varying lengths. (minutes [‡])	
														additional 12 optional monthly group sessions	
Bertz 2012[33]	Control	Control	No			No									No
	Diet Only	Diet only	No	Nutrition Edu.	Dietitian	No	Individual	Face to Face; Telephone; SMS	Health Care; Community; Home	3	3	2	Week 0, 6	1.5 hrs at start of intervention, 1hr at week 6	Yes
	Exercise only	Exercise only	No		Physiotherapist	No	Individual	Face to Face; Telephone; SMS	Health Care; Community; Home	3	3	2	Week 0, 6	1.5 hours at start of intervention, 1 hour at week 6	No
	Intervention	Diet and exercise	No	Nutrition Edu.	Dietitian; Physiotherapist	No	Individual	Face to Face; Telephone; SMS	Health Care; Community; Home	3	3	4	Week 0, 6	2 x 1.5 hours at start of intervention,<br>2 x 1 hour at week 6	Yes
Bo 2007[34]	Control standard care	Control	No	Nutrition Edu.	GP	Yes	Individual	Face to Face	Health Care	0		1			No
	Intervention lifestyle by trained professional	Diet and exercise	No	Nutrition Edu.	Physician; Nutritionist	Yes	Individual and Group	Face to Face; Print	Health Care; Community	12	12	6		60	Yes
Burke 2005[35]	Control usual care	Control	No			No		Face to Face; Print	Health Care						
	Low sodium + fish diet	Diet and exercise	No	Nutrition Edu.	Physician; Dietitian	No	Individual and Group	Face to Face; Telephone; Print;	Health Care; Home	16	4	18	6 group plus individual (0-4m). Then, group: 2 x month for 1st month. 1 x month for 2 months. Then 1 x every 3m	Group = 90 mins. Length of individual sessions not stated.	
Chaiyasoot 2018[36]	Control, Lifestyle Education Intervention	Diet and exercise	No	Nutrition Edu.	Dietitian	No	Individual and Group	Face to Face; Print		3	3	5	Baseline, 2, 4, 8, 12 weeks	30	Yes
	Lifestyle	Diet and	No	MR-P; Nutrition	Dietitian	No	Individual	Face to		3	3	5	Baseline, 2, 4, 8, 12	30	Yes

Study ID [Ref No.*]	Groups:	Intervention type	Intervention faded in intensity	Features (meal replacements, nutrition education, financial incentives, intermittent fasting, content designed to help participants following programme end)	Provider [†]	Provider training received	Delivery		Intervention setting	Intervention timing (months)		Sessions			Intervention personalised, titrated or adapted
							Mode	Format		Last contact	End (step change in intensity)	N [§]	Frequency	Length per session with description for varying lengths. (minutes [‡])	
	Education Intervention plus Meal Replacements	exercise		Edu.			and Group	Face; Print							
Chee 2017[37]	Usual Care	Diet and exercise	Yes	Nutrition Edu.	Dietitian	Unclear	Unclear	Face to Face; Print	Health Care	6	12	4	Every 3m		Yes
	tDNA Conventional Counseling	Diet and exercise	Yes	MR-P; Nutrition Edu.	Physician; Dietitian	Unclear	Unclear	Face to Face; Print	Health Care	6	12	8	Monthly 0-6m then every 3m		Yes
	tDNA Motivational Interviewing	Diet and exercise	Yes	MR-P; Nutrition Edu.	Physician; Dietitian	Unclear	Unclear	Face to Face; Print	Health Care	6	12	8	Monthly 0-6m then every 3m		Yes
Cheskin 2008[38]	Standard diet	Diet and exercise	Yes	Nutrition Edu.	Dietitian	No	Individual and Group	Face to Face	Community; Home	20	8	36	Group: every 2 weeks for 0-34 weeks, then mthly 12-20m. 3 individual meetings		Yes
	Meal replacement	Diet and exercise	Yes	MR-P; Nutrition Edu.	Dietitian	No	Individual and Group	Face to Face	Community; Home	20	8	36	Group: every 2 weeks for 0-34 weeks, then mthly 12-20m. 3 individual meetings		Yes
Cheyette 2007[39]	Control	Diet only	No	Nutrition Edu.	Dietitian	No	Individual	Face to Face	Health Care			1 minimum	Annually		No
	Weight No More intervention group	Diet and exercise	No	Nutrition Edu.; Help following programme end	Dietitian; Physiotherapist	Unclear	Group	Face to Face; Print	Community	4	4	8	Fortnightly	1.5 hours	No
Christensen 2012[40]	Reference group	Control	No	Nutrition Edu.		No	Group	Face to Face		12		12	Monthly	2 hours	No
	Intervention group	Diet and exercise	No	Nutrition Edu.; Help following programme end		No	Group	Face to Face	Workplace	12	3	48	Weekly	1 hour; Participants also instructed to spend additional personal time	Yes

Study ID [Ref No.*]	Groups:	Intervention type	Intervention faded in intensity	Features (meal replacements, nutrition education, financial incentives, intermittent fasting, content designed to help participants following programme end)	Provider [†]	Provider training received	Delivery		Intervention setting	Intervention timing (months)		Sessions			Intervention personalised, titrated or adapted
							Mode	Format		Last contact	End (step change in intensity)	N[§]	Frequency	Length per session with description for varying lengths. (minutes [‡])	
														doing physical exercise (see 'Procedures')	
Cole 2013[41]	Control - individualised counselling	Diet and exercise	No	Nutrition Edu.	Dietitian	No	Individual	Face to Face	Health Care	3	3	1	At least 1 session over 3m	45 – 60	No
	Intervention-shared medical appointment	Diet and exercise	No	Nutrition Edu.	Nurse (General); Dietitian; Nutritionist	No	Group	Face to Face	Health Care	3	3	3	3 sessions over 3m	90	No
Conroy 2015[42]	Self-guided	Diet and exercise	No	Nutrition Edu.	Other	No	Other - Self-guided manual	Print	Home	3	3			12-week self-guided manual	No
	Interventionist led	Diet and exercise	No	Nutrition Edu.	Physician; Other	No	Group	Face to Face	Health Care	3	3	12	Weekly	60 mins	No
Crowley 2017[43]	Group Medical Visit		Yes		Nurse (General); GP; Physician; Dietitian	Unclear	Individual and Group	Face to Face; Print	Health Care	11.1	3.7	8	Every 4 weeks for 16 weeks and every 8 weeks until week 48	1.5-2 hours	Yes
	Intensive Weight Management Group Medical Visit	Diet and exercise	Yes	Nutrition Edu.; Help following programme end	Nurse (General); GP; Physician; Dietitian	Unclear	Individual and Group	Face to Face; Print	Health Care	11.1	3.7	16	Every 2 weeks for 16 weeks and every 8 weeks until week 48	1.5-2 hours	Yes
Dalziel 2006[44]	Control	Control	No	Nutrition Edu.	Physician; Dietitian	No	Unclear	Face to Face	Health Care						No
	Experimental	Diet only	No	Nutrition Edu.	Physician; Dietitian	No	Unclear	Face to Face	Health Care	24	2	3	At 8 weeks, then annually from baseline	1 hour first session, length of follow-up sessions not reported.	No
Damschroder 2014[45]	Control, MOVE - usual care	Diet and exercise	Yes	Nutrition Edu.	Nurse (General); Psychologist/ Counsellor; Dietitian; Physiotherapist	Unclear	Group	Face to Face	Health Care	24	3	58	Weekly for 3m, then either quarterly or twice monthly	11-12 weekly open-group sessions of 90 mins each over 3 months. During months 4-12, one group met	Yes

Study ID Ref No.*	Groups:	Intervention type	Intervention faded in intensity	Features (meal replacements, nutrition education, financial incentives, intermittent fasting, content designed to help participants following programme end)	Provider †	Provider training received	Delivery		Intervention setting	Intervention timing (months)		Sessions			Intervention personalised, titrated or adapted
							Mode	Format		Last contact	End (step change in intensity)	N§	Frequency	Length per session with description for varying lengths. (minutes ‡)	
														quarterly for 90 minutes and the other groups met twice a month for 60 minutes. Some participants had the option of re-enrolling in the initial series of weekly sessions. Total hours over the year ranged from 22 to 35 hours. 12-24 mths as above for mths 4-12.	
	ASPIRE group, individual telephone counselling	Diet and exercise	Yes	Nutrition Edu.	Health Trainer	Yes	Individual	Telephone	Home	24	3	34	Same as ASPIRE-Group but duration of sessions varied	Up to 30 mins for the first 3 mths and 20 mins for the remaining 9 mths, totalling 11 hours across the year. 12-24 mths coaching every other mth, 6 sessions.	Yes
	ASPIRE group, group counselling	Diet and exercise	Yes	Nutrition Edu. Help following programme end	Health Trainer	Yes	Group	Face to Face	Health Care	24	3	34	Both ASPIRE small-changes treatment arms consisted of weekly sessions for 3m, followed by 6m of sessions every other week, and then 3 monthly sessions over 12 months, for a total of 28 sessions.	Up to 90 mins for the first 3 mths and 60 mins for the remaining 9 mths, totalling 33 hours across the year. 12-24 mths coaching every other mth, 6 sessions.	Yes
Daubenmier	Active control	Diet and exercise	Yes	Nutrition Edu.	Dietitian	Unclear	Group	Face to Face; Print	Community	5.5	5.5	16	12 weekly then biweekly for 3	16 sessions: 2 hours; 5-hour all-	Yes

Study ID Ref No.*	Groups:	Intervention type	Intervention faded in intensity	Features (meal replacements, nutrition education, financial incentives, intermittent fasting, content designed to help participants following programme end)	Provider †	Provider training received	Delivery		Intervention setting	Intervention timing (months)		Sessions			Intervention personalised, titrated or adapted
							Mode	Format		Last contact	End (step change in intensity)	N§	Frequency	Length per session with description for varying lengths. (minutes ‡)	
2016[46]	intervention												sessions, and then one session one month later plus a single all-day weekend session	day session	
	Mindfulness Intervention	Diet and exercise	Yes	Nutrition Edu.; Help following programme end	Dietitian	Yes	Group	Face to Face; Print	Community	5.5	5.5	16	12 weekly then biweekly for 3 sessions, and then one session one month later plus a single all-day weekend session	16 sessions: 2.5 hours; 6.5 hour all day session	Yes
Daumit 2013[47]	Control, Usual care	Control	No	Nutrition Edu.		No	Other					1			
	ACHIEVE	Diet and exercise	Yes	Nutrition Edu.	Health Trainer	Yes	Individual and Group	Face to Face	Health Care	18	6	279	1-6m: Group weight management class weekly, individual visit monthly, group PA class 3x per week, weigh-in weekly; 7-18m: monthly group and individual class, group PA class 3x week, weigh in weekly	Group weight management class = 45 mins; Individual visit with interventionist 15-20 mins; group PA class 45 mins, weigh-in = 2 mins	Yes
deVos 2016[48]	Control	Control	No			No									
	Tailor-made lifestyle intervention	Diet and exercise	No	Nutrition Edu.	Dietitian; Physiotherapist	No	Individual and Group	Face to Face	Community	30	6	23 +		First 3 dietician appointments were biweekly, after that the frequency of visits was determined by mutual agreement. Invited to attend 20 weekly physical activity classes.	Yes

Study ID [Ref No.*]	Groups:	Intervention type	Intervention faded in intensity	Features (meal replacements, nutrition education, financial incentives, intermittent fasting, content designed to help participants following programme end)	Provider †	Provider training received	Delivery		Intervention setting	Intervention timing (months)		Sessions			Intervention personalised, titrated or adapted
							Mode	Format		Last contact	End (step change in intensity)	N§	Frequency	Length per session with description for varying lengths. (minutes ‡)	
														"...definition of compliance as attendance at ≥ 6 dietitian visits and ≥7 physical activity classes."	
Delahanty 2015[49]	Dietitian Referral group	Diet and exercise	No	Nutrition Edu.		Unclear	Individual	Face to Face; Print	Health Care			1 +	Individualised – 1 session plus follow-up sessions on individual basis at dietitian's discretion	Initial session (1 hour); follow-up sessions (20-40 minutes)	Yes
	Group lifestyle intervention	Diet and exercise	No	MR-P; Nutrition Edu.	Physician; Dietitian	Yes	Group	Face to Face; Print	Health Care	6	6	19	Weekly	1.5 hours	No
Djuric 2002[50]	Control	Control	No	Nutrition Edu.	Dietitian	No	Unclear	Print		0	0	1			
	Weight Watchers	Diet and exercise	No	Nutrition Edu.	Dietitian	Yes	Group	Face to Face	Community	12	12	52	Weekly		
	Individualized group	Diet and exercise	Yes	Nutrition Edu.	Dietitian		Individual and Group	Face to Face; Telephone; Print	Community	12	3	21	0-3m: weekly; 3-6m: every other week; 6-12m: monthly		Yes
	Comprehensive group	Diet and exercise	Yes	Nutrition Edu.	Dietitian		Individual and Group	Face to Face; Telephone	Community	12	3	73	Weekly		Yes
Duncan 2016[51]	Control	Control	No		GP	No	Individual	Face to Face	Health Care			1			No
	Intervention	Diet and exercise	No	Nutrition Edu.; Help following programme end	Health Trainer	Yes	Individual	Face to Face	Home	4	4	6	Ranged from 1-4 weeks	60	Yes
Eakin 2014[52]	Usual care	Control	No	Nutrition Edu.		Unclear	Individual	Print	Health Care			0			No
	Telephone intervention	Diet and exercise	Yes	Nutrition Edu.	Health Trainer	Yes	Individual	Telephone; Print	Health Care	18	6	27	4 weekly calls; fortnightly calls for 5 months; monthly calls for 12 months.		Yes
Fernandez-Ruiz	Control	Control	No			Unclear									No
	Intervention (healthy	Diet and exercise	No	Nutrition Edu.	Nurse (General);	Unclear	Individual and Group	Face to Face	Health Care	12	12	232	4 x per week physical activity;	208 exercise sessions: 4 x per	Yes

Study ID [Ref No.*]	Groups:	Intervention type	Intervention faded in intensity	Features (meal replacements, nutrition education, financial incentives, intermittent fasting, content designed to help participants following programme end)	Provider †	Provider training received	Delivery		Intervention setting	Intervention timing (months)		Sessions			Intervention personalised, titrated or adapted
							Mode	Format		Last contact	End (step change in intensity)	N§	Frequency	Length per session with description for varying lengths. (minutes ‡)	
2018[53]	eating, exercise & CBT)				Physician; Psychologist/ Counsellor; Nutritionist; Exercise physiologist								monthly CBT & health ed.	week, 40 mins. CBT 12 sessions, 1 per month, 60 mins. Health education (nurse) 12 sessions, 1 per month, 60 mins.	
Fisher 2011[54]	Diet only	Diet only	No	MR-F		No		Face to Face	Health Care	6	6	0			No
	Diet + aerobic training	Diet and exercise	No	MR-F	Exercise physiologist	No	Group	Face to Face	Health Care; Community	6	6	78		50	No
	Diet + resistance training	Diet and exercise	No	MR-F	Exercise physiologist	No	Group	Face to Face	Health Care; Community	6	6	78		50	No
Foley 2016[55]	Usual care (Control)	Control	No			No	Other	Print	Health Care						No
	Weight loss intervention	Diet and exercise	Yes	Nutrition Edu.	Psychologist/ Counsellor; Health care professional (not specified); Other	No	Individual	Face to Face; Telephone; Internet; App; Print; SMS	Health Care; Home	12	3	18	Calls 1-4: weekly; Calls 5-10: biweekly Calls 11-18: monthly		No
Foster-Schubert 2012[56]	Control- usual care	Control	No			No	Other – no contact								
	Calorie reduced diet	Diet only	Yes	Nutrition Edu.	Dietitian	No	Individual and Group	Face to Face; Telephone; Internet	Community; Home	12	6	38	2 + 24 weekly 0-24. Then 2 per month (12) during weeks 24 – 52		Yes
	Aerobic exercise (N/A)	N/A	N/A	N/A	N/A	N/A	N/A	N/A	N/A	N/A	N/A	N/A	N/A	N/A	N/A
	Intervention - diet and exercise	Diet and exercise	Yes	Nutrition Edu.	Dietitian; Exercise physiologist;	No	Individual and Group	Face to Face; Telephone; Internet	Community; Home	12	6	194	3 per week exercise. Plus 38 diet sessions.	45	Yes
Fuller	Western diet	Diet and	No		Dietitian	No	Individual	Face to	Health Care;	3	3	1			No

Study ID [Ref No.*]	Groups:	Intervention type	Intervention faded in intensity	Features (meal replacements, nutrition education, financial incentives, intermittent fasting, content designed to help participants following programme end)	Provider [†]	Provider training received	Delivery		Intervention setting	Intervention timing (months)		Sessions			Intervention personalised, titrated or adapted
							Mode	Format		Last contact	End (step change in intensity)	N[§]	Frequency	Length per session with description for varying lengths. (minutes [‡])	
2012[57]	group	exercise						Face	Community						
	Korean diet group	Diet and exercise	No	MR-F	Dietitian	No	Individual	Face to Face	Health Care; Community	3	3	1			No
Green 2015[58]	Usual care	Control	No	Nutrition Edu.			Unclear								No
	STRIDE	Diet and exercise	Yes	Nutrition Edu.	Psychologist/ Counsellor; Nutritionist		Individual and Group	Face to Face; Telephone; Print;	Health Care	12	6	3	Weekly: 0- 6m; Monthly: 6-12m	120	Yes
Hageman 2017[59]	Web-based only	Diet and exercise	Yes	Nutrition Edu.; Help following programme end	Nurse (General)	No	Individual	Internet	Home	30	6	61	0-6m: weekly. 6-18m: biweekly; 18-24m: monthly 24-30m: bimonthly	0 to 6m new content weekly posted weekly. 6 to 18m posted biweekly. Videos monthly, 18 to 24 months Videos bimonthly, 24 to 30 months. Weight logging daily.	No
	Web-based discussion	Diet and exercise	Yes	Nutrition Edu.; Help following programme end	Nurse (General); Other	Yes	Individual	Internet; Other	Home	30	6	109	0-6m: weekly; 6-18m: biweekly; 18-24m: monthly; 24-30m: bimonthly	As for WO Plus O-6 mths weekly primers. 6-12 mths biweekly primers. 12-18 mths monthly primers.	Yes
	Web-based email	Diet and exercise	Yes	Nutrition Edu.; Help following programme end	Nurse (General); Psychologist/ Counsellor; Dietitian	No	Individual	Internet; Other	Home	30	6	106	0-6m: weekly; 6-18m: biweekly; 18-24m: monthly; 24-30m: bimonthly	As for WO. Plus O-6 mths weekly feedback. 6-12 mths biweekly feedback. 12-18 mths monthly feedback.	Yes
Hardcastle 2013[60]	Control	Diet and exercise	No	Nutrition Edu.	Nurse (General)	Yes	Other - Single appointment; provided	Print	Health Care	6	6	1	Once		No

Study ID [Ref No.*]	Groups:	Intervention type	Intervention faded in intensity	Features (meal replacements, nutrition education, financial incentives, intermittent fasting, content designed to help participants following programme end)	Provider [†]	Provider training received	Delivery		Intervention setting	Intervention timing (months)		Sessions			Intervention personalised, titrated or adapted
							Mode	Format		Last contact	End (step change in intensity)	N[§]	Frequency	Length per session with description for varying lengths. (minutes [‡])	
							leaflet								
	MI counselling intervention	Diet and exercise	No	Nutrition Edu.	Psychologist/ Counsellor; Personal Trainer	Yes	Individual	Face to Face	Health Care	6	6	5	Anytime over a 6m period	20 – 30	Yes
Harrigan 2016[61]	Usual Care Group	Diet and exercise	No			Unclear	Unclear	Unclear;	Health Care			2			No
	Telephone Weight Loss Counseling	Diet and exercise	Yes		Dietitian	No	Individual	Telephone	Home	6	6	11	Once per week (month 1), then every two weeks (months 2 and 3), and once per month (months 4, 5, and 6)	30	No
	In-Person Weight Loss Counseling	Diet and exercise	Yes		Dietitian	No	Individual	Face to Face	Health Care	6	6	11	Once per week (month 1), then every two weeks (months 2 and 3), and once per month (months 4, 5, and 6)	30	No
Hunt 2014[62]	Control, Wait-list	Control	No	Nutrition Edu.				Print							
	FFIT	Diet and exercise	Yes	Nutrition Edu.; Help following programme end	Personal Trainer	Yes	Group	Face to Face; Print	Community	12	3	19	Weekly for 12 weeks. 6 emails over 9 months. 1 reunion at 6 months.	1.5 hours for 12 weekly sessions. Then 6 emails over 9m and 1 x reunion at 6m.	Yes
Irwin 2003[63]	Control Group	Control	No							0	0	0	0	0	No
	Exercise group	Exercise onl	Yes	Help following programme end	Exercise physiologist		Group	Face to Face	Community; Home	12	3	36	3 times per week for the first 3 months and one per week the rest 9 months	45	No
Jebb 2011[64]	Standard care	Diet and exercise	No	Nutrition Edu.	GP	Yes	Individual	Face to Face	Health Care	0	0	1	Single session		
	Commercial programme	Diet and exercise	No	Nutrition Edu.	Health Trainer	Unclear	Group	Face to Face; Internet	Community	12	12	52	Weekly	60	Yes
Jebb	Usual care	Diet only		Nutrition Edu.	Nurse	No	Individual	Face to	Health Care	3	3	6 to 12	Weekly or biweekly		Yes

Study ID Ref No.*	Groups:	Intervention type	Intervention faded in intensity	Features (meal replacements, nutrition education, financial incentives, intermittent fasting, content designed to help participants following programme end)	Provider †	Provider training received	Delivery		Intervention setting	Intervention timing (months)		Sessions			Intervention personalised, titrated or adapted
							Mode	Format		Last contact	End (step change in intensity)	N§	Frequency	Length per session with description for varying lengths. (minutes ‡)	
2017[65]					(General)			Face; Print							
	Low energy total diet replacement programme	Diet only	Yes	MR-F; Nutrition Edu.; Help following programme end	Health Trainer	Yes	Individual	Face to Face	Community	6	6	15	Weekly for first 12 weeks, then monthly		Yes
Jenkins 2017[66]	Control	Control	No	Nutrition Edu.		Unclear		Print	Health Care						No
	Dietary advice only	Diet only	Yes	Nutrition Edu.		Unclear	Individual	Telephone; Print	Health Care	6	6	9	Weekly (Month 1); Monthly (Months 0-5)	20 – 30	Yes
	Food basket only	Diet only	No			Unclear	Individual	Print; Other	Residential	6	6	Received 26 food baskets	Weekly		No
	Food and advice	Diet only	Yes	Nutrition Edu.		Unclear	Individual	Telephone; Print; Other	Health Care	6	6	9 plus 26 food baskets	Weekly (Month 1); Monthly (Months 0-5); weekly baskets	20 – 30	Yes
Katula 2013[67]	Enhanced Usual Care Comparison Condition	Diet and exercise	No	Nutrition Edu.	Dietitian	Unclear	Individual	Face to Face; Print	Community	24		2	Both sessions during first 3 months		Yes
	Lifestyle Weight-Loss Intervention	Diet and exercise	Yes	Nutrition Edu.; Help following programme end	Dietitian; Health Trainer	Yes	Individual and Group	Face to Face; Telephone	Community	24	6	65	Months 1-6: Weekly group sessions plus "All participants received three personalized consultations with an RD (during Months 1, 3, and 6)."; Months 7-24: 2 contacts per month, one group session and one phone contact		Yes
Katzer 2008[68]	Mail-delivered 'non-dieting'	Diet and exercise	Yes	Nutrition Edu.; Help following programme end		No	Other – print only	Print	Home	2.3	10.3	0			No

Study ID [Ref No.*]	Groups:	Intervention type	Intervention faded in intensity	Features (meal replacements, nutrition education, financial incentives, intermittent fasting, content designed to help participants following programme end)	Provider [†]	Provider training received	Delivery		Intervention setting	Intervention timing (months)		Sessions			Intervention personalised, titrated or adapted
							Mode	Format		Last contact	End (step change in intensity)	N[§]	Frequency	Length per session with description for varying lengths. (minutes [‡])	
	program (P3)														
	Group 'non-dieting' program (P2)	Diet and exercise	Yes	Nutrition Edu.; Help following programme end	Psychologist/ Counsellor; Dietitian; Health Trainer	Unclear	Group	Face to Face	Community	2.3	10.3	22	Weekly for 10 weeks, fortnightly then monthly	2 hours	No
	Group 'non-dieting' program plus Relaxation (P1)	Diet and exercise	Yes	Nutrition Edu.; Help following programme end	Psychologist/ Counsellor; Nutritionist	Unclear	Group	Face to Face; Other	Community; Home	2.3	10.3	22	Weekly for 10 weeks, fortnightly then monthly	2 hours	No
King 1989[69]	Control (N/A)	N/A	N/A	N/A	N/A	N/A	N/A	N/A	N/A	N/A	N/A	N/A	N/A	N/A	N/A
	Exercise only	Exercise only				Unclear	Unclear	Face to Face; Print	Community		12				Yes
	Diet only	Diet only		Nutrition Edu.	Nutritionist	Unclear	Individual and Group	Face to Face	Community		12				Yes
Knauper 2018[70]	Standard DPP	Diet and exercise	Yes	Nutrition Edu.	Health Trainer	Yes	Group	Face to Face	Community	12	3	22	12 weekly core sessions, 4 transitional sessions over 3 months, and 6 monthly support sessions	1 hour	
	Enhanced DPP	Diet and exercise	Yes	Nutrition Edu.; Help following programme end	Health Trainer	Yes	Group	Face to Face; Print	Community	12	3	22	12 weekly core sessions, 4 transitional sessions over 3 months, and 6 monthly support sessions	1 hour	Yes
Diabetes Prevention Program R G 2009[71]	Placebo	Diet and exercise	No	Nutrition Edu.	Other		Individual	Face to Face; Print		36	36	4	Annually	20 – 30	
	Metformin (N/A)	N/A	N/A	N/A	N/A	N/A	N/A	N/A	N/A	N/A	N/A	N/A	N/A	N/A	N/A
	Lifestyle	Diet and exercise	Yes	MR-P; Nutrition Edu.; Help following programme end	Psychologist/ Counsellor; Dietitian; Exercise physiologist; Health	Yes	Individual and Group	Face to Face; Telephone; Print	Community	36	6	358	16 sessions in first 24 weeks then monthly. [At least 2 exercise classes per week]	45 mins; Core curriculum sessions 30-60 mins; Sessions: 16+6+12+12=46.	Yes

Study ID Ref No.*	Groups:	Intervention type	Intervention faded in intensity	Features (meal replacements, nutrition education, financial incentives, intermittent fasting, content designed to help participants following programme end)	Provider †	Provider training received	Delivery		Intervention setting	Intervention timing (months)		Sessions			Intervention personalised, titrated or adapted
							Mode	Format		Last contact	End (step change in intensity)	N§	Frequency	Length per session with description for varying lengths. (minutes ‡)	
					Trainer									[Physical activity 2x52 x 3 years = 312]	
Kuller 2012[72]	Control - health education	Control	Yes						Community			0			
	Intervention - lifestyle change	Diet and exercise	Yes	Nutrition Edu.	Psychologist/ Counsellor; Nutritionist; Exercise physiologist	No	Group	Face to Face	Community	36	6	64	1-6m: weekly<br>6-12m: every 2 weeks<br>12-36m: monthly		Yes
Kumanyika 2012[73]	Basic programme	Diet and exercise	No	Nutrition Edu.; Help following programme end	Nurse (General); Physician; Other AHPs	Yes	Individual	Face to Face; Print	Health Care; Home	12	12	3	PCP every 4 months. 12 printed session in year 1. 2 printed sessions in year 2.	12.5	Yes
	Basic plus programme	Diet and exercise	Yes	Nutrition Edu.; Help following programme end	Nurse (General); Physician; Other AHPs; Health Trainer	Yes	Individual	Face to Face; Print	Health Care; Home	12	12	15	PCP every 4 months. LC monthly year 1, every other month year 2. 12 printed session in year 1.	12.5	Yes
Ley 2004[74]	Control diet	Control	No												
	Reduced-fat	Diet only		Nutrition Edu.				Face to Face		12	12	12			
Li 2016[75]	Usual care group	Control	Yes		Dietitian; Other	Yes		Print	Inpatient	12	1	0			Yes
	Diet group	Diet only	Yes	Help following programme end	Dietitian; Other	Yes	Group	Face to Face; Telephone; Internet; Print	Inpatient	12	1	36	Six weekly sessions (Month 1); Monthly sessions (Months 1 - 12)		Yes
	50g-oats group	Diet only	Yes	Help following programme end	Dietitian; Other	Yes	Group	Face to Face; Telephone; Internet; Print	Inpatient	12	1	36	Six weekly sessions (Month 1); Monthly sessions (Months 1 - 12)		Yes
	100g-oats	Diet only	Yes	Help following	Dietitian;	Yes	Group	Face to	Inpatient	12	1	36	Six weekly sessions		Yes

Study ID Ref No.*	Groups:	Intervention type	Intervention faded in intensity	Features (meal replacements, nutrition education, financial incentives, intermittent fasting, content designed to help participants following programme end)	Provider †	Provider training received	Delivery		Intervention setting	Intervention timing (months)		Sessions			Intervention personalised, titrated or adapted
							Mode	Format		Last contact	End (step change in intensity)	N§	Frequency	Length per session with description for varying lengths. (minutes ‡)	
	group			programme end	Other			Face; Telephone; Internet; Print					(Month 1); Monthly sessions (Months 1 - 12)		
Li 2005[76]	Individualized diet plan	Diet only	Yes	Nutrition Edu.; Help following programme end	Dietitian	Unclear	Individual	Face to Face	Community	12	2	15	Months 1-2: Every 2 weeks; Months 2-12: Monthly		Yes
	Soy-based meal replacement	Diet only	Yes	MR-P; MR-F; Nutrition Edu.; Help following programme end	Dietitian	Unclear	Individual	Face to Face; Other	Community	12	3	15	Months 1-2: Every 2 weeks; Months 2-12: Monthly		Yes
Lindstrom 2003[77]	Control	Control	No	Nutrition Edu.	Nurse (General); Physician; Nutritionist	Unclear	Other - group or individual	Face to Face; Print	Health Care			1		30 mins to 1 hour	No
	Intervention	Diet and exercise	Yes	MR-P; MR-F; Nutrition Edu.; Help following programme end	Nurse (General); Physician; Nutritionist; Physiotherapist	Unclear	Individual and Group	Face to Face; Telephone; Print	Health Care; Community	48	12	19	7 sessions in first year then every 3 months	30 mins to 1 hour	Yes
Liss 2016[78]	Standard care arm	Diet and exercise	No	Nutrition Edu.	Health care professional (not specified); Other	No	Individual	Face to Face; Print	Health Care	12		3	Every 6m	"brief"	Yes
	Standard care plus group-based lifestyle intervention	Diet and exercise	Yes	Nutrition Edu.; Help following programme end	Health Trainer	Yes	Individual and Group	Face to Face; Print	Health Care; Community	12	6	39	Weekly for 6m; biweekly for next 6m	3 brief; 60-to-90-minute intervention sessions	Yes
Little 2016[79]	Control, Nurse follow-up	Diet only	No	Nutrition Edu.	Nurse (General)	No	Other	Internet	Home				Data collection only at 6 and 12 months		No
	Web-based support with minimal support	Diet and exercise	No	Nutrition Edu.; Help following programme end	Nurse (General)	Unclear	Individual	Telephone; Internet; Other	Home	6	6	29	24 web-based sessions designed to be used over 6 months. Three		Yes

Study ID [Ref No.*]	Groups:	Intervention type	Intervention faded in intensity	Features (meal replacements, nutrition education, financial incentives, intermittent fasting, content designed to help participants following programme end)	Provider [†]	Provider training received	Delivery		Intervention setting	Intervention timing (months)		Sessions			Intervention personalised, titrated or adapted
							Mode	Format		Last contact	End (step change in intensity)	N[§]	Frequency	Length per session with description for varying lengths. (minutes [‡])	
	(Remote)												scheduled phone or email contacts and up to two optional phone or email contacts in the first 6 months		
	Web-based + nurse support (face to face)	Diet and exercise	No	Nutrition Edu.; Help following programme end	Nurse (General)	Unclear	Individual	Face to Face; Telephone; Internet; Other	Health Care; Home	6	6	31	24 web-based sessions designed to be used over 6 months. Three scheduled face-to-face appointments in the first 3 months, and then up to four more appointments during a further 3 months if needed.		Yes
Manning 1994[80]	Clinic visit	Diet only	Yes	Nutrition Edu.	Dietitian	No	Individual	Face to Face	Health Care	12	6	7	6 weekly intervals for the first 6 months and then 2 monthly for the remainder of the year.		Yes
	Behavioural	Diet only	Yes	Nutrition Edu.	Psychologist/ Counsellor; Dietitian; Physiotherapist	No	Group	Face to Face	Health Care	12	3	10	Fortnightly intervals initially for 3 months and then at 2 monthly intervals for the remainder of the year.		Yes
	Home visits	Diet only	Yes	Nutrition Edu.	Dietitian	No	Individual	Face to Face	Health Care; Home	12	6	7	6 weekly intervals for the first 6 months and then 2 monthly for the remainder of the year.		Yes
	Dexfenfluramine (N/A)	N/A	N/A	N/A	N/A	N/A	N/A	N/A	N/A	N/A	N/A	N/A	N/A	N/A	N/A
	Routine usual care (N/A)	N/A	N/A	N/A	N/A	N/A	N/A	N/A	N/A	N/A	N/A	N/A	N/A	N/A	N/A

Study ID [Ref No.*]	Groups:	Intervention type	Intervention faded in intensity	Features (meal replacements, nutrition education, financial incentives, intermittent fasting, content designed to help participants following programme end)	Provider [†]	Provider training received	Delivery		Intervention setting	Intervention timing (months)		Sessions			Intervention personalised, titrated or adapted
							Mode	Format		Last contact	End (step change in intensity)	N[§]	Frequency	Length per session with description for varying lengths. (minutes [‡])	
Meffer d 2007[81]	Control	Control	No			Unclear	Unclear								
	Intervention	Diet and exercise	Yes	Nutrition Edu.; Help following programme end		Unclear	Individual and Group	Face to Face; Telephone	Health Care	4	12	78	16 weeks of weekly closed group sessions followed by once-monthly sessions and then monthly sessions for an additional 6 months; Telephone contact: twice weekly during initial two weeks; weekly thereafter		Yes
Melcha rt 2017[82]	Control group	Diet and exercise	No	Nutrition Edu.	GP	No		Face to Face; Print	Health Care						Yes
	Intervention group	Diet and exercise	Yes	Nutrition Edu.; Help following programme end	Health Trainer	Yes	Individual and Group	Face to Face; Internet	Health Care	3	12	17	10 weekly; 3 full day 'introduction days' (reduction phase); 4 full day refresh training sessions (maintenance phase)	7 full-day sessions; 10 x 2 hour sessions	Yes
Melin 2003[83]	Control, less intensively treated	Diet and exercise	No	Nutrition Edu.; Help following programme end	Physician; Psychologist/ Counsellor; Dietitian		Group	Face to Face	Health Care	24	24	27	2 x per week during VLCD (2 periods 25 days) + every 3m		No
	Intensively treated	Diet and exercise	Yes	Nutrition Edu.; Help following programme end	Physician; Psychologist/ Counsellor; Dietitian		Group	Face to Face	Health Care	12	24	43	2 x per week during VLCD (2 periods 25 days) + every fortnight during the first year and 6 meetings during the second year.		No
Menar d 2005[84]	Control - usual care	Control	No	Nutrition Edu.	Physician			Face to Face	Health Care			6	Every 6m	Health ed materials at 6, 12 and 18 mths, plus 3 phone calls	
	Intervention -	Diet and	No	Nutrition Edu.			Individual	Face to	Health Care;	12	12	36	3 x per month	1 session plus at	Yes

Study ID [Ref No.*]	Groups:	Intervention type	Intervention faded in intensity	Features (meal replacements, nutrition education, financial incentives, intermittent fasting, content designed to help participants following programme end)	Provider [†]	Provider training received	Delivery: Mode	Delivery: Format	Intervention setting	Intervention timing (months): Last contact	Intervention timing (months): End (step change in intensity)	Sessions: N[§]	Sessions: Frequency	Sessions: Length per session with description for varying lengths. (minutes [‡])	Intervention personalised, titrated or adapted
	intensive multitherapy	exercise						Face; Telephone; Print	Home					least 2 phone calls per month	
Mengham 1999[85]	Control		No	Nutrition Edu.	Dietitian	Unclear	Unclear	Face to Face	Health Care	12	12	3	Six-monthly	15	Yes
	Intervention		No	Nutrition Edu.; Help following programme end	Dietitian	Unclear	Individual and Group	Face to Face	Health Care	6	12	16	Fortnightly up to 6 months plus 6 monthly sessions	15 mins standard care sessions; Patients in the intervention group typically received input from the dietitian amounting to 3hrs over the twelve months of the study.	Yes
Mensinger 2016[86]	Control, Weight Neutral Program	Diet and exercise	No	Nutrition Edu.; Help following programme end	Psychologist/ Counsellor; Personal Trainer		Group	Face to Face; Print; Other	Community	6	6	26	Weekly.	90	
	Weight Loss Program	Diet and exercise	No	Nutrition Edu.; Help following programme end			Group	Face to Face; Print; Other	Community	6	6	26	Weekly	90	
Mitsui 2008[87]	Control	Control	No			Unclear	Unclear		Health Care			0			No
	Intervention	Diet and exercise	Yes	Nutrition Edu.	Dietitian	Unclear	Individual and Group	Face to Face	Health Care	3	12	25	Weekly: 0-12; Every other week: 13-26; Monthly 26-52 weeks	Exercise training: 40 minutes; Individual counselling sessions: not reported	Yes
Moreno 2014[88]	Low-calorie diet	Diet and exercise	Yes	Nutrition Edu.	Physician; Dietitian; Other	No	Individual and Group	Face to Face; Telephone	Health Care	12	12	9	LC diet. Group meetings took place at 0.5, 2, 4, 6, 8, 10, and 12 months		Yes
	Very low-calorie-ketogenic diet	Diet and exercise	Yes	MR-P; Nutrition Edu.	Physician; Dietitian; Other	No	Other	Face to Face; Telephone	Health Care	12	2	9	VLCK diet up to 2m (45-60 days). Meetings same as LCD group		Yes

Study ID Ref No.*	Groups:	Intervention type	Intervention faded in intensity	Features (meal replacements, nutrition education, financial incentives, intermittent fasting, content designed to help participants following programme end)	Provider †	Provider training received	Delivery		Intervention setting	Intervention timing (months)		Sessions			Intervention personalised, titrated or adapted
							Mode	Format		Last contact	End (step change in intensity)	N§	Frequency	Length per session with description for varying lengths. (minutes ‡)	
Morgan 2010[89]	Control (Information and self-help)	Control	No	Nutrition Edu.	Other	Unclear	Group	Face to Face; Print	Community			1	Once	60	No
	SHED-IT (Internet) group	Diet and exercise	Yes	Nutrition Edu.	Other	Unclear	Individual and Group	Face to Face; Internet; Print	Community; Home	3	3	8	Submit online daily eating and exercise diaries for the first 4 weeks, for 2 weeks in the second month and for 1 week in the third month. 7 x feedback	1st session face to face group- 75 mins. The rest internet. 7 feedback sessions. Submit online daily eating and exercise diaries for the first 4 weeks, for 2 weeks in the second month and for 1 week in the third month. 28 + 14 + 7 = 49	Yes
Muggia 2014[90]	Standard care group	Diet only	No	Nutrition Edu.		No	Individual	Print	Home	12	6	7	Control meetings every 3 months during the first year and every 6 months during the second year	30	No
	Brief CBT group	Diet only	No	Nutrition Edu.		No	Group	Face to Face; Print	Health Care	12	6	14	7 treatment sessions in a monthly basis. Then control meetings every 3 months during the first year and every 6 months during the second year	90	No
Nakata 2014[91]	Control (N/A)	N/A	N/A	N/A	N/A	N/A	N/A	N/A	N/A	N/A	N/A	N/A	N/A	N/A	N/A
	Education-only	Diet and exercise	No	Nutrition Edu.	Other	No	Group	Face to Face; Print	Health Care	6	6	1		2 hours	No
	Group-based support	Diet and exercise	Yes	Nutrition Edu.	Other	Yes	Group	Face to Face; Print	Health Care	6	6	8	Fortnightly (Weeks 1-6); Monthly (Weeks 6 - 22)	2 hours	No

Study ID [Ref No.*]	Groups:	Intervention type	Intervention faded in intensity	Features (meal replacements, nutrition education, financial incentives, intermittent fasting, content designed to help participants following programme end)	Provider †	Provider training received	Delivery		Intervention setting	Intervention timing (months)		Sessions			Intervention personalised, titrated or adapted
							Mode	Format		Last contact	End (step change in intensity)	N§	Frequency	Length per session with description for varying lengths. (minutes ‡)	
Nanchahal 2012[92]	Usual care control			Nutrition Edu.	GP	Yes	Individual	Face to Face; Print	Health Care						Yes
	CAMWEL Intervention	Diet and exercise	Yes	Nutrition Edu.; Help following programme end	Health Trainer	Yes	Individual	Face to Face; Print	Health Care	6	12	14	Fortnightly for 12 weeks, 3-weekly to 27 weeks, 4-weekly to 35 weeks and a 12-week interval to the last session	30	Yes
Ng 2015[93]	Control group	Control	No	Nutrition Edu.	Physician	No	Individual	Face to Face	Health Care	6		2	Single sessions at baseline and at 6 months		
	Lifestyle modification program	Diet and exercise	Yes	Nutrition Edu.; Help following programme end	Dietitian	No	Individual	Face to Face	Health Care	12	4	24	Weekly (Months 1-4); Monthly (Months 5-12)	Encouraged to see an exercise instructor at least once during the program and perform 30 min of aerobic exercise two to three times a week.	
Nilsen 2011[94]	Control, Individual Physician Group	Diet and exercise	No	Nutrition Edu.	Physician		Individual	Face to Face	Health Care	18	18	3	6 monthly		
	Individual Plus Interdisciplinary Group	Diet and exercise	Yes	Nutrition Edu.; Help following programme end	Nurse (General); Physician; Dietitian; Physiotherapist; Other		Individual and Group	Face to Face	Health Care; Community	18	6	11	Weekly (Weeks 5 to 10. Other sessions at week 3, 16, 20, 26 52 and 78	7 sessions of 5 hours;<br>1 x individual session 30 mins;<br>3 x physician consultation (30 mins?<br>[3 x exercise test]	Yes
Nordby 2012[95]	Control	Control	No			Unclear			Community			0			No
	Training and increased diet (N/A)	N/A	N/A	N/A	N/A	N/A	N/A	N/A	N/A	N/A	N/A	N/A	N/A	N/A	N/A
	Training	Exercise only	No			Unclear	Unclear	Face to Face; Print	Community	3	3	12	Weekly	3–4 sessions/week of continuous	Yes

Study ID Ref No.*	Groups:	Intervention type	Intervention faded in intensity	Features (meal replacements, nutrition education, financial incentives, intermittent fasting, content designed to help participants following programme end)	Provider †	Provider training received	Delivery		Intervention setting	Intervention timing (months)		Sessions			Intervention personalised, titrated or adapted
							Mode	Format		Last contact	End (step change in intensity)	N§	Frequency	Length per session with description for varying lengths. (minutes ‡)	
														exercise at moderate intensity; d 3–4 sessions/week of continuous exercise with intermittent high intensity training intervals; weekly contact with a supervisor; extra supervision incorporated when required	
	Energy-reduced diet	Diet only	No	Nutrition Edu.		Unclear	Unclear	Face to Face	Community	3	3	12	Weekly	Weekly contact with a supervisor; extra supervision incorporated when required.	
Oldroyd 2006[96]	Control group	Control	No												
	Intervention group	Diet and exercise	No	Nutrition Edu.	Dietitian; Physiotherapist	No	Individual	Face to Face; Print	Community	24	6	12	In the first 6 months there were three such appointments at two weekly intervals, followed by three at monthly intervals. There was one after 9 months and five at two monthly intervals between 12 and 24 months.	15 – 20	Yes
Pan 1997[97]	Control	Control	No	Nutrition Edu.	Physician	Unclear	Unclear	Print	Health Care						No
	Intervention group (Exercise: n=155; Diet: n = 148; Diet		Yes	Nutrition Edu.	Physician	Unclear	Individual and Group	Face to Face; Print	Health Care	72	72	30 - 60	Frequency of group dietary counselling and exercise sessions: weekly for 1 month, monthly		Yes

Study ID Ref No.*	Groups:	Intervention type	Intervention faded in intensity	Features (meal replacements, nutrition education, financial incentives, intermittent fasting, content designed to help participants following programme end)	Provider †	Provider training received	Delivery		Intervention setting	Intervention timing (months)		Sessions			Intervention personalised, titrated or adapted
							Mode	Format		Last contact	End (step change in intensity)	N§	Frequency	Length per session with description for varying lengths. (minutes ‡)	
	plus exercise: n = 135)												for 3 months, and then once every 3 months for the remainder of the study.		
Parikh 2010[98]	Control	Control					Unclear		Health Care						
	Intervention	Diet and exercise	No	Nutrition Edu.		Yes	Group	Face to Face; Print	Community	3	3	8	8 sessions over 10 weeks	1.5 hours	No
Pedersen 2013[99]	Aerobic interval training	Exercise only	Yes	Nutrition Edu.; Help following programme end	Physiotherapist	Unclear	Group	Face to Face	Community	12	3	116	Three times a week for 12 weeks; twice weekly for 40 weeks.	38	
	Low energy diet	Diet and exercise	Yes	MR-P; Nutrition Edu.; Help following programme end	Dietitian	Unclear	Individual and Group	Face to Face	Community	12	3	96	Fortnightly dietitian sessions (Weeks 1-12); Monthly (Weeks 12 – 52); Twice weekly exercise sessions for 40 weeks.	Exercise sessions: 38 mins; Dietitian sessions not reported	
Pettman 2009 [100]	Control	Control	No	Nutrition Edu.		No	Individual	Print							No
	Intervention B - Passive follow-up	Diet and exercise	No	Nutrition Edu.	Health Trainer	Unclear	Group	Face to Face; Telephone; Other	Community	4	12	32	Weekly group session and exercise session	2 hour group sessions: 1 hour exercise session.	No
	Intervention A - Active follow-up	Diet and exercise	Yes	Nutrition Edu.; Help following programme end	Health Trainer	Unclear	Group	Face to Face; Telephone; Other	Community	4	12	40	Weekly group session and exercise session	2 hour group sessions; 1 hour exercise session.	No
Promrat 2010 [101]	Control	Diet and exercise	No	Nutrition Edu.	Nutritionist; Health Trainer	Unclear	Group	Face to Face	Health Care	12	12	4	Once every 12 weeks.		
	Lifestyle Intervention	Diet and exercise	Yes	Nutrition Edu.; Help following programme end	Nutritionist; Health Trainer	Unclear	Individual and Group	Face to Face	Health Care	12	6	36	Months 1-6: weekly; Months 7-12: biweekly		Yes
Provencher 2009[102]	Control group	Control	No												
	Social support	Diet only	No	Nutrition Edu.	Psychologist/ Counsellor; Dietitian	Yes	Group	Face to Face	Community	4	4	14	Weekly	2 hours	Yes

Study ID [Ref No.*]	Groups:	Intervention type	Intervention faded in intensity	Features (meal replacements, nutrition education, financial incentives, intermittent fasting, content designed to help participants following programme end)	Provider [†]	Provider training received	Delivery		Intervention setting	Intervention timing (months)		Sessions			Intervention personalised, titrated or adapted
							Mode	Format		Last contact	End (step change in intensity)	N[§]	Frequency	Length per session with description for varying lengths. (minutes [‡])	
	Health-At-Every-Size	Diet and exercise	No	Nutrition Edu.	Psychologist/ Counsellor; Dietitian	Yes	Group	Face to Face; Print	Community	4	4	14	Weekly	"13 three-hour evening sessions and 1 intensive-day session of 6 hours)."	Yes
Ridgeway 1999[103]	Control	Control	No			No									No
	Intervention Group	Diet and exercise	No	Nutrition Edu.	Nurse (General); Dietitian	Unclear	Individual and Group	Face to Face; Print	Health Care	12	6	7	Monthly; Plus single follow-up session at 12 months	90 minutes group sessions	Yes
Rock 2015[104]	Control	Diet and exercise	Yes	Nutrition Edu.	Other	Unclear	Individual	Face to Face; Telephone; Internet	Community	6	12	14	2 individual sessions (baseline and 6 months); monthly telephone calls and/or e-mails		Yes
	Intervention	Diet and exercise	Yes	Nutrition Edu.; Help following programme end	Other	Unclear	Individual and Group	Face to Face; Telephone; Internet	Community	6	12	73	Group sessions: weekly for 4 months; biweekly for two months; monthly for 6 months; Newsletters: quarterly from 6 to 24 months.	1 hour group sessions; Group sessions were reinforced by brief (10- to 15-minute) personalized guidance delivered by telephone and/or e-mail.	Yes
Rolls 2005[105]	Comparison-control	Control	No			No									
	Two snacks	Diet and exercise	No	MR-P; Nutrition Edu.; Help following programme end	Dietitian	No	Individual	Face to Face		12	6	24	Weekly from 1 to 3 months, fortnightly 4 to 6 months, and monthly from 7 to 12 months	15 – 30	No
	One soup	Diet and exercise	No	MR-P; Nutrition Edu.; Help following programme end	Dietitian	No	Individual	Face to Face		12	6	24	Weekly from 1 to 3 months, fortnightly 4 to 6 months, and monthly from 7 to 12 months	15 – 30	No
	Two soups	Diet and exercise	No	MR-P; Nutrition Edu.; Help	Dietitian	No	Individual	Face to Face		12	6	24	Weekly from 1 to 3 months, fortnightly	15 - 30	No

Study ID [Ref No.*]	Groups:	Intervention type	Intervention faded in intensity	Features (meal replacements, nutrition education, financial incentives, intermittent fasting, content designed to help participants following programme end)	Provider [†]	Provider training received	Delivery		Intervention setting	Intervention timing (months)		Sessions			Intervention personalised, titrated or adapted
							Mode	Format		Last contact	End (step change in intensity)	N [§]	Frequency	Length per session with description for varying lengths. (minutes [‡])	
				following programme end									4 to 6 months, and monthly from 7 to 12 months		
Rolls 2017[106]	Standard advice	Diet and exercise	Yes	Nutrition Edu.	Dietitian; Other	Yes	Individual	Face to Face; Print	Community	12	1	19	Weekly in month 1 and biweekly in months 2–6, and 1-hour sessions were scheduled monthly in months 7–12	Thirty-min weekly and biweekly sessions; 1-hour monthly sessions.	Yes
	Pre-portioned foods group	Diet and exercise	Yes	MR-P; Nutrition Edu.; Help following programme end	Dietitian; Other	Yes	Individual	Face to Face; Print	Community	12	1	19	Weekly in month 1 and biweekly in months 2–6, and 1-hour sessions were scheduled monthly in months 7–12	Thirty-min weekly and biweekly sessions; 1-hour monthly sessions.	Yes
	Portion selection group	Diet and exercise	Yes	Nutrition Edu.; Help following programme end	Dietitian; Other	Yes	Individual	Face to Face; Print	Community	12	1	19	Weekly in month 1 and biweekly in months 2–6, and 1-hour sessions were scheduled monthly in months 7–12	Thirty-min weekly and biweekly sessions; 1-hour monthly sessions.	Yes
Rosas 2015[107]	Usual care		No		GP	No	Individual	Face to Face	Health Care						No
	Case-management intervention		Yes	Nutrition Edu.; Help following programme end	Health Trainer	No	Individual and Group	Face to Face	Health Care	24	6	20	16 sessions from 0-12 months. 4 sessions from 12-24 months.	Group sessions last 2 hours, individual sessions last 30 minutes.	Yes
	Case-management + Community health worker intervention		Yes	Nutrition Edu.; Help following programme end	Health Trainer	No	Individual and Group	Face to Face	Health Care; Community; Home	24	6	27	Same as CM group, with additional 5 home visits from 0-12 months and 2 home visits from 12-24 months.	Same as CM group. The length of the additional CHW home visits is not clear.	Yes
Ross 2012[108]	Control condition	Control	No	Nutrition Edu.	Physician	No	Unclear	Face to Face	Health Care				Usual schedule (typically once a year).		Yes
	Behavioral	Diet and	No	Nutrition Edu.; Help	Health	Yes	Individual	Face to		24	6	33	First 6m: 8 sessions	(0–6 months, 15	Yes

Study ID [Ref No.*]	Groups:	Intervention type	Intervention faded in intensity	Features (meal replacements, nutrition education, financial incentives, intermittent fasting, content designed to help participants following programme end)	Provider [†]	Provider training received	Delivery		Intervention setting	Intervention timing (months)		Sessions			Intervention personalised, titrated or adapted
							Mode	Format		Last contact	End (step change in intensity)	N[§]	Frequency	Length per session with description for varying lengths. (minutes [‡])	
	intervention group	exercise		following programme end	Trainer		and Group	Face					in first 6 weeks, then every 2 weeks. Months 7-24, monthly sessions.	sessions, 15 hours); Months 7–12 (6 sessions, 3–6 hours); Months 13–24 (12 sessions, 6–12 hours)	
Samaras 1997[109]	Control	Control	No												
	Intervention	Exercise only	No	Help following programme end	Nurse (General); Physician; Dietitian; Exercise physiologist; Other	Yes	Group	Face to Face; Print; Video	Community	6	6	6	Monthly	1 hour	Yes
Sattin 2016[110]	Health Education intervention	Control	No	Nutrition Edu.	Health care professional (not specified)	Yes	Group	Face to Face	Community	9	3	18	Weekly for the first 12 weeks and monthly for the remaining 6m		Yes
	Fit body and soul intervention	Diet and exercise	No	Nutrition Edu.; Help following programme end	Health care professional (not specified)	Yes	Group	Face to Face	Community	9	3	18	Weekly for the first 12 weeks and monthly for the remaining 6m		Yes
Schubel 2016[111]	Control group	Control	Yes	Nutrition Edu.	Dietitian; Nutritionist	Yes	Individual	Face to Face; Telephone	Community	11.5	3	8	Biweekly phone calls (Week 1-12); Two single sessions at the beginning and end of the Intervention phase	"The number of personal contacts and counseling sessions was the same for all study participants overall, but individuals in the ICR and CCR arms received longer and more comprehensive counseling sessions with personalized dietary plans,	No

Study ID [Ref No.*]	Groups:	Intervention type	Intervention faded in intensity	Features (meal replacements, nutrition education, financial incentives, intermittent fasting, content designed to help participants following programme end)	Provider [†]	Provider training received	Delivery		Intervention setting	Intervention timing (months)		Sessions			Intervention personalised, titrated or adapted
							Mode	Format		Last contact	End (step change in intensity)	N[§]	Frequency	Length per session with description for varying lengths. (minutes [‡])	
														specific for the ICR or CCR regimens."	
	Continuous Calorie Restriction	Diet only	Yes	Nutrition Edu.; Help following programme end	Dietitian; Nutritionist	Yes	Individual	Face to Face; Telephone; Print	Community	11.5	3	8	Biweekly phone calls (Week 1-12); Two single sessions at the beginning and end of the Intervention phase	"The number of personal contacts and counseling sessions was the same for all study participants overall, but individuals in the ICR and CCR arms received longer and more comprehensive counseling sessions with personalized dietary plans, specific for the ICR or CCR regimens."	Yes
	Intermittent Calorie Restriction	Diet only	Yes	Nutrition Edu.; Inter. Fasting; Help following programme end	Dietitian; Nutritionist	Yes	Individual	Face to Face; Telephone; Print	Community	11.5	3	8	Biweekly phone calls (Week 1-12); Two single sessions at the beginning and end of the Intervention phase	"The number of personal contacts and counseling sessions was the same for all study participants overall, but individuals in the ICR and CCR arms received longer and more comprehensive counseling sessions with personalized dietary plans, specific for the	Yes

Study ID [Ref No.*]	Groups:	Intervention type	Intervention faded in intensity	Features (meal replacements, nutrition education, financial incentives, intermittent fasting, content designed to help participants following programme end)	Provider [†]	Provider training received	Delivery		Intervention setting	Intervention timing (months)		Sessions			Intervention personalised, titrated or adapted
							Mode	Format		Last contact	End (step change in intensity)	N[§]	Frequency	Length per session with description for varying lengths. (minutes [‡])	
														ICR or CCR regimens."	
Seligman 2011[112]	Standard-of-care strategy	Diet only		Nutrition Edu.		Unclear	Individual	Print		3	3				Yes
	Healthy diet and step counter	Diet and exercise		Nutrition Edu.		Unclear	Individual	Face to Face; Print		3	3	2			No
	Healthy diet and fitness	Diet and exercise		Nutrition Edu.		Unclear	Individual	Face to Face; Print		3	3	2			Yes
Shikany 2013[113]	Food-based diet	Diet only	Yes	Nutrition Edu.		No	Individual	Face to Face; Telephone; Internet; Print	Health Care; Home	12	6	10	Fortnightly until week 4, monthly until week 20, then six weeks, followed by one fortnight, followed by one month to week 32, two months to week 40 and then six weekly until week 52.		Yes
	Meal replacement	Diet only	Yes	MR-P; Nutrition Edu.	Dietitian; Health Trainer	No	Individual	Face to Face; Telephone; Internet	Health Care; Home	12	6	10	Fortnightly until week 4, monthly until week 20, then six weeks, followed by one fortnight, followed by one month to week 32, two months to week 40 and then six weekly until week 52.		Yes
Snel 2012[114]	VLCD only	Diet only	No	MR-F; Nutrition Edu.		Unclear	Unclear	Face to Face	Health Care	4	4				No
	VLCD + exercise	Diet and exercise	No	MR-F; Nutrition Edu.	Physiotherapist	Unclear	Unclear	Face to Face	Health Care; Home	4	4	16	Weekly at minimum	One-hour supervised exercise sessions plus at least 4 home training	No

Study ID [Ref No.*]	Groups:	Intervention type	Intervention faded in intensity	Features (meal replacements, nutrition education, financial incentives, intermittent fasting, content designed to help participants following programme end)	Provider [†]	Provider training received	Delivery		Intervention setting	Intervention timing (months)		Sessions			Intervention personalised, titrated or adapted
							Mode	Format		Last contact	End (step change in intensity)	N[§]	Frequency	Length per session with description for varying lengths. (minutes [‡])	
														sessions	
Steven s 1993[115]	Control	Control	No			No				0	0	0			No
	Intervention	Diet and exercise	Yes	Nutrition Edu.; Help following programme end	Psychologist/ Counsellor; Dietitian; Exercise physiologist	Unclear	Individual and Group	Face to Face; Telephone; Internet; Print	Community	18	3	54		90 minutes group, individual length not reported.<br><br>'The intervention started with an individual counseling session, followed by 14 weekly group meetings led by dietitians or health educators. After this 14-week intensive phase, participants attended six biweekly group meetings and then monthly group meetings. Beginning in the 18th month, participants were offered a variety of options to keep them involved in the intervention, including individual counseling sessions and special group sessions focused on selected weight loss topics.'	Yes
Steven	Control	Control	No			Unclear						0			

Study ID [Ref No.*]	Groups:	Intervention type	Intervention faded in intensity	Features (meal replacements, nutrition education, financial incentives, intermittent fasting, content designed to help participants following programme end)	Provider [†]	Provider training received	Delivery		Intervention setting	Intervention timing (months)		Sessions			Intervention personalised, titrated or adapted
							Mode	Format		Last contact	End (step change in intensity)	N[§]	Frequency	Length per session with description for varying lengths. (minutes [‡])	
s 2001[116]	Intervention	Diet and exercise	Yes	Nutrition Edu.; Help following programme end	Dietitian; Health Trainer	Unclear	Individual and Group	Face to Face; Telephone; Print; Other	Community	36	6	51	Intensive phase (0-6 Months): 1 individual, 14 weekly, 6 biweekly sessions. Extended phase (7-36 Months): biweekly contacts with monthly face-to-face meetings until the intensive intervention is completed for the first cohort then mini-modules to be offered with continued biweekly contact. Specifically tailored follow-up where indicated.	90 mins in first phase	Yes
	Sodium only intervention (N/A)	N/A	N/A	N/A	N/A	N/A	N/A	N/A	N/A	N/A	N/A	N/A	N/A	N/A	N/A
	Combined intervention (N/A)	N/A	N/A	N/A	N/A	N/A	N/A	N/A	N/A	N/A	N/A	N/A	N/A	N/A	N/A
Sundfor 2018[117]	Continuous energy restriction	Diet only	Yes	Nutrition Edu.; Help following programme end	Dietitian	Unclear	Individual	Face to Face; Telephone; Print; Internet	Health Care	12	6	10	"Follow-up visits were scheduled at biweekly intervals up to eight weeks, and thereafter monthly up to six months for a total of 10 visits."		Yes
	Intermittent energy restriction	Diet only	Yes	Nutrition Edu.; Inter. Fasting; Help following programme end	Dietitian	Unclear	Individual	Face to Face; Telephone; Print; Internet	Health Care	12	6	10	"Follow-up visits were scheduled at biweekly intervals up to eight weeks, and thereafter		Yes

Study ID [Ref No.*]	Groups:	Intervention type	Intervention faded in intensity	Features (meal replacements, nutrition education, financial incentives, intermittent fasting, content designed to help participants following programme end)	Provider [†]	Provider training received	Delivery		Intervention setting	Intervention timing (months)		Sessions			Intervention personalised, titrated or adapted
							Mode	Format		Last contact	End (step change in intensity)	N[§]	Frequency	Length per session with description for varying lengths. (minutes [‡])	
													monthly up to six months for a total of 10 visits.		
Tapsell 2017[118]	Usual care (Control)	Diet and exercise	Yes	Nutrition Edu.	Nurse (General)	Unclear	Individual	Face to Face; Telephone; Print	Health Care	12	3	11	Months 1-3: Monthly; Months 1 – 12: Quarterly; Phone calls: Quarterly	30 mins clinics; 15 min phone calls	Yes
	Intervention Group	Diet and exercise	Yes	Nutrition Edu.	Dietitian; Health Trainer	Yes	Individual	Face to Face; Telephone; Print	Health Care	12	3	11	Months 1-3: Monthly; Months 1 – 12: Quarterly; Phone calls: Quarterly	1 hour clinics; 15 min phone calls	Yes
	Intervention plus food supplement group (N/A)	N/A	N/A	N/A	N/A	N/A	N/A	N/A	N/A	N/A	N/A	N/A	N/A	N/A	N/A
TarragaMarcos 2017[119]	G3	Diet and exercise	No	Nutrition Edu.		Unclear	Group	Face to Face	Health Care			1			No
	G2	Diet and exercise	Yes	Nutrition Edu.		Unclear	Group	Face to Face; Internet; Other	Health Care; Home	12	3	6	After the initial visit, visits were scheduled after 15 days, 1m, 3m, 6m and one year.		No
	G1	Diet and exercise	Yes	Nutrition Edu.; Help following programme end	Nurse (General)	Unclear	Group	Face to Face	Health Care	8	3	10	Every two weeks from weeks, 1 to 12 and then monthly from weeks 13 to 32	1 hour	No
Teeriniemi 2018[120]	Control	Control	No	Nutrition Edu.		No	Other	Print	Health Care			0			Yes
	SHG Counselling	Diet and exercise	No	Nutrition Edu.	Nurse (General)	Yes	Group	Face to Face	Community	0.7	0.7	2		90	No
	CBT Counselling	Diet and exercise	Yes	Nutrition Edu.	Nutritionist	No	Group	Face to Face	Community	4.1	4.1	8	7 sessions every second week, last session after 1 month	90	No
	Control plus	Diet and	No	Nutrition Edu.; Help		No	Other	Internet;	Health Care;	12	12	0		52-week access to	Yes

Study ID Ref No.*	Groups:	Intervention type	Intervention faded in intensity	Features (meal replacements, nutrition education, financial incentives, intermittent fasting, content designed to help participants following programme end)	Provider †	Provider training received	Delivery		Intervention setting	Intervention timing (months)		Sessions			Intervention personalised, titrated or adapted
							Mode	Format		Last contact	End (step change in intensity)	N§	Frequency	Length per session with description for varying lengths. (minutes ‡)	
	HBCSS	exercise		following programme end				Print	Community					Web-based HBCSS	
	SHG Counselling plus HBCSS	Diet and exercise	No	Nutrition Edu.; Help following programme end	Nurse (General)	Yes	Group	Face to Face; Internet	Community	12	12	2		90 minutes 52-week access to Web-based HBCSS	Yes
	CBT Counselling plus HBCSS	Diet and exercise	Yes	Nutrition Edu.; Help following programme end	Nutritionist	No	Group	Face to Face; Internet	Community	12	12	8	7 sessions every second week, last session after 1 month	90 minutes 52-week access to Web-based HBCSS	Yes
ter Bogt 2009[121]	GP usual care	Control	No		GP	Unclear	Individual	Face to Face	Health Care			1		10	No
	Lifestyle counselling from NP	Diet and exercise	Yes	Nutrition Edu.	Nurse (General)	Yes	Individual	Face to Face; Telephone	Health Care	36	8	11	Four visits (at months 1, 2, 3, 8); 1 telephone call (5 months) in the first year; one visit and one telephone call per year (year 2, 3)	Average duration of the visits was 35 minutes for the first and second visit (range 15–60 minutes) and 25 minutes for the third visit (range 15–40 minutes).	Yes
Tsai 2010[122]	Control	Control	No	Nutrition Edu.	GP	No	Individual	Face to Face; Print	Health Care	12	12	4	Quarterly	2 – 3	No
	Brief counselling	Diet and exercise	No	Nutrition Edu.	Other AHPs; Health care professional (not specified)	Yes	Individual	Face to Face; Telephone; Print	Health Care	12	6	12	PCP visits: quarterly. MA visits: weeks 0, 2, 4, 8, 12, 16, 20, 24	15 – 20	Yes
Tuomilehto 2009[123]	Control	Diet and exercise	No	Nutrition Edu.	Nurse (General); Physician	Yes	Individual			12		3	At baseline, 3ms and 12m		No
	Intervention	Diet and exercise	Yes	MR-F; Nutrition Edu.	Nutritionist; Physiotherapist	Unclear	Individual and Group	Face to Face	Health Care; Home	12	3	14	Every 2 weeks until week 12 then monthly	60 – 90	Yes
van de Glind	Comparison group	Control	No	Nutrition Edu.		No		Print	Community						No

Study ID [Ref No.*]	Groups:	Intervention type	Intervention faded in intensity	Features (meal replacements, nutrition education, financial incentives, intermittent fasting, content designed to help participants following programme end)	Provider [†]	Provider training received	Delivery		Intervention setting	Intervention timing (months)		Sessions			Intervention personalised, titrated or adapted
							Mode	Format		Last contact	End (step change in intensity)	N[§]	Frequency	Length per session with description for varying lengths. (minutes [‡])	
2017[124]	EuroFIT group	Diet and exercise	No	Nutrition Edu.; Help following programme end	Health Trainer	Yes	Group	Face to Face; App; Print; Other	Community	6 - 9	3	13	Weekly for Weeks 1 to 12; One reunion meeting held 6–9 months after the program end.	90	Yes
vanWier 2011[125]	Control – Brochure	Control	No	Nutrition Edu.			Other – information booklet								No
	Internet Group	Diet and exercise	No	Nutrition Edu.	Psychologist/ Counsellor; Health Trainer	Yes	Individual	Internet	Workplace; Home	6	6	10	Every 2 weeks	Work on module on internet. Email contact after completion of each module.	No
	Phone Group	Diet and exercise	No	Nutrition Edu.	Psychologist/ Counsellor; Health Trainer	Yes	Individual	Telephone	Workplace; Home	6	6	10	Every 2 weeks	Call every 2 weeks. Work on modules individually in between calls.	No
Vissers 2010[126]	Control	Control	No			No									
	Diet only group (Diet)	Diet only	Yes	Nutrition Edu.	Dietitian	Unclear	Individual	Face to Face	Community	12	3	12	During the first 3 months participants had a dietary counseling every fortnight. During the next 3 months there was a dietary counseling once a month. 3 more visits months 6-12		Yes
	Diet + fitness training group (Fitness)	Diet and exercise	Yes	Nutrition Edu.	Dietitian; Physiotherapist	Unclear	Individual and Group	Face to Face	Health Care; Community; Home	12	3	51	As per diet only plus 2 x week for first 3m, 1 x week for second 3m		Yes
	Diet + WBV group (Vibration)	Diet and exercise	Yes	Nutrition Edu.	Dietitian; Physiotherapist	Unclear	Individual and Group	Face to Face	Community; Home	12	3	51	As per diet only plus 2 x week for first 3m, 1 x week for second 3m		Yes
Volpe	Exercise only	Exercise	Yes	Help following	Other	Yes	Group	Face to	Community;	12	6	96	3/4/5 days/week	30	

Study ID Ref No.*	Groups:	Intervention type	Intervention faded in intensity	Features (meal replacements, nutrition education, financial incentives, intermittent fasting, content designed to help participants following programme end)	Provider †	Provider training received	Delivery		Intervention setting	Intervention timing (months)		Sessions			Intervention personalised, titrated or adapted
							Mode	Format		Last contact	End (step change in intensity)	N§	Frequency	Length per session with description for varying lengths. (minutes ‡)	
2008[127]		only		programme end				Face; Telephone; Internet	Home				exercise sessions; Monthly and periodic phone/email contact		
	Diet only	Diet only	Yes	Nutrition Edu.; Help following programme end		Unclear	Group	Face to Face; Telephone; Internet	Community	12	6	18	Weekly; biweekly nutrition sessions; Monthly; periodically phone/email contact		
	Combination of diet and exercise	Diet and exercise	Yes	Nutrition Edu.; Help following programme end	Other	Yes	Group	Face to Face; Telephone; Internet	Community; Home	12	6	114	3/4/5 days/week exercise sessions; Weekly; biweekly nutrition sessions; Monthly; periodically phone/email contact	30 mins exercise sessions; duration of nutritional sessions not reported.	
Weinstock 2013[128]	Conference Call DPP	Diet and exercise	Yes	Nutrition Edu.	Nurse (General); Dietitian; Other	Yes	Group	Telephone; Print	Health Care	24	24	40 plus 6 optional	Educators: weekly-5 weeks, monthly-1 year; Coaches: Monthly (Year 1)		Yes
	Individual Call DPP	Diet and exercise	Yes	Nutrition Edu.	Nurse (General); Dietitian; Other	Yes	Individual	Telephone; Print	Health Care	24	24	40 + 6 optional	Educators: weekly-5 weeks, monthly-1 year; Coaches: Monthly (Year 1)		Yes
West 2007[129]	Attention control	Diet and exercise	Yes	Nutrition Edu.; Help following programme end	Nutritionist; Exercise physiologist; Health Trainer	No	Group	Face to Face		18	6	47	Weekly for 6m, Biweekly for 6m, and then monthly for 6m.	45	No
	Motivational interviewing	Diet and exercise	Yes	Nutrition Edu.; Help following programme end	Psychologist/ Counsellor; Nutritionist; Exercise physiologist; Health Trainer	No	Individual and Group	Face to Face		18	6	47	Weekly for 6m, Biweekly for 6m, and then monthly for 6m. Five individual motivational interviewing sessions were offered, with the first session before	45	Yes

Study ID [Ref No.*]	Groups:	Intervention type	Intervention faded in intensity	Features (meal replacements, nutrition education, financial incentives, intermittent fasting, content designed to help participants following programme end)	Provider [†]	Provider training received	Delivery		Intervention setting	Intervention timing (months)		Sessions			Intervention personalised, titrated or adapted
							Mode	Format		Last contact	End (step change in intensity)	N[§]	Frequency	Length per session with description for varying lengths. (minutes [‡])	
													starting group therapy and then at 3, 6, 9, and 12m.		
The Look AHEAD Research Group 2010[130]	Diabetes support and education	Diet and exercise	No	Nutrition Edu.; Help following programme end	Nurse (General); Dietitian; Health Trainer; Personal Trainer	Yes	Group	Face to Face; Telephone; Print; Other	Community		48	22	3 sessions annually for the first 4 years of follow-up; thereafter, one session was provided annually	60 – 90	No
	Intensive lifestyle intervention	Diet and exercise	Yes	MR-P; Nutrition Edu.; Help following programme end	Nurse (General); Physician; Psychologist/ Counsellor; Dietitian; Personal Trainer	Yes	Individual and Group	Face to Face; Telephone; Internet; Print; Other	Community	115	12	134	Months: 1-6: weekly; Months 7-12: 3/month; Years 2-4: Minimum of 1/month; Year 5+: Monthly recommended.	Months 1-6: Group sessions: 60 to 75 minutes; Individual sessions: 20 to 30 minutes.	Yes
Wing 1988[131]	Diet plus placebo exercise	Diet only	Yes	Nutrition Edu.; Fin. Incentives; Help following programme end			Group	Face to Face	Health Care; Home	8.5	2.5	26	Both groups participated in a behavioural weight control programme, with group meetings held twice a week for 10 weeks and monthly for the following 6m.	1 hour	No
	Diet plus moderate exercise	Diet and exercise	Yes	Nutrition Edu.; Fin. Incentives; Help following programme end			Group	Face to Face	Health Care; Home	8.5	2.5	26	Both groups participated in a behavioural weight control programme, with group meetings held twice a week for 10 weeks and monthly for the following 6m.	1 hour	No
Wing 1988b[1]	Diet only	Diet only	Yes	Nutrition Edu.; Fin. Incentives; ; Help	Psychologist/ Counsellor	No	Group	Face to Face	Health Care; Home	14	2.5	52	Both groups attended treatment	1 hour	No

Study ID [Ref No.*]	Groups:	Intervention type	Intervention faded in intensity	Features (meal replacements, nutrition education, financial incentives, intermittent fasting, content designed to help participants following programme end)	Provider [†]	Provider training received	Delivery		Intervention setting	Intervention timing (months)		Sessions			Intervention personalised, titrated or adapted
							Mode	Format		Last contact	End (step change in intensity)	N[§]	Frequency	Length per session with description for varying lengths. (minutes [‡])	
[31]				following programme end									sessions 3 times/week (versus 2 times/week in Study 1) for 10 weeks. After this intensive training period, subjects met weekly for an additional 10 weeks and then monthly for a year.		
	Diet plus exercise	Diet and exercise	Yes	Nutrition Edu.; Fin. Incentives; Help following programme end	Psychologist/ Counsellor	No	Group	Face to Face	Health Care; Home	14	2.5	52	Both groups attended treatment sessions 3 times/week (versus 2 times/week in Study 1) for 10 weeks. After this intensive training period, subjects met weekly for an additional 10 weeks and then monthly for a year.	1 hour	No
Wing 1991[132]	Behavior therapy alone	Diet and exercise	Yes	Nutrition Edu.	Psychologist/ Counsellor	No	Group	Face to Face	Inpatient; Home	17	5	25	Weekly meetings for 20 weeks, Maintenance meetings at 24, 28, 46, 72 weeks		No
	Behavior therapy plus VLCD	Diet and exercise	No	MR-P; Nutrition Edu.	Psychologist/ Counsellor	No	Group	Face to Face	Inpatient; Home	17	5	31	Weekly meetings for 20 weeks, Maintenance meetings at 24, 28, 46, 72 weeks PLUS biweekly meetings with the physician for 3m.		No
Wing 1998[133]	Control	Diet and exercise	No	Nutrition Edu.		No		Print							No

Study ID [Ref No.*]	Groups:	Intervention type	Intervention faded in intensity	Features (meal replacements, nutrition education, financial incentives, intermittent fasting, content designed to help participants following programme end)	Provider †	Provider training received	Delivery		Intervention setting	Intervention timing (months)		Sessions			Intervention personalised, titrated or adapted
							Mode	Format		Last contact	End (step change in intensity)	N§	Frequency	Length per session with description for varying lengths. (minutes ‡)	
	Diet	Diet only	No	Nutrition Edu.; Help following programme end	Psychologist/ Counsellor; Dietitian	No	Group	Face to Face	Community; Home	24	6	51	Weekly for the first 6m; Biweekly for the next 6m		No
	Exercise	Exercise only	No		Psychologist/ Counsellor; Exercise physiologist	No	Group	Face to Face	Community; Home	24	6	51	Weekly for the first 6m; Biweekly for the next 6m	50 – 60 min walk with the therapist at each of these weekly meetings.	No
	Diet plus exercise	Diet and exercise	No	Nutrition Edu.; Help following programme end	Psychologist/ Counsellor; Dietitian; Exercise physiologist	No	Group	Face to Face	Community; Home	24	6	51	Weekly for the first 6m; Biweekly for the next 6m		No
Yannakoulia 2008[134]	Usual care group	Control	No	Nutrition Edu.	Dietitian	No	Individual	Face to Face	Health Care			1			Yes
	Intensive care group	Diet and exercise	No	Nutrition Edu.	Dietitian	No	Individual	Face to Face	Health Care	2	2	5	Every two weeks		Yes
Yates 2009[135]	Control group	Control	No			No		Print				0			No
	PREPARE group	Diet and exercise	No	Nutrition Edu.	Health Trainer	No	Individual and Group	Face to Face	Community	6	6	3	1, 3 and 6m	The first session lasted 180 min and the follow-up review progress lasted 10 mins.	Yes
	PREPARE with pedometer	Diet and exercise	No	Nutrition Edu.	Health Trainer	No	Individual and Group	Face to Face	Community	6	6	3	1, 3 and 6m	The first session lasted 180 min and the follow-up review progress lasted 10 mins.	Yes
Yeh 2003[136]	Counseling based intervention	Diet and exercise	Yes	Nutrition Edu.; Help following programme end	Dietitian	No	Individual	Face to Face	Community	6	6	6	Monthly	2 x 1 hour. 4 x 30 min.	Yes
	Skills based intervention	Diet only	No	Nutrition Edu.; Help following programme end	Dietitian	No	Individual and Group	Face to Face; Telephone; Internet	Community; Home	6	6	7	Monthly	2 x 90 mins<br>2 x 2 hour (supermarket)<br>2 x 90 mins (restaurant)<br>1 x 2 hr (home)	Yes

Study ID Ref No.*	Groups:	Intervention type	Intervention faded in intensity	Features (meal replacements, nutrition education, financial incentives, intermittent fasting, content designed to help participants following programme end)	Provider †	Provider training received	Delivery		Intervention setting	Intervention timing (months)		Sessions			Intervention personalised, titrated or adapted
							Mode	Format		Last contact	End (step change in intensity)	N§	Frequency	Length per session with description for varying lengths. (minutes ‡)	
Yeh 2016[137]	Control group	Control	No			No									
	Intervention group	Diet and exercise	Yes	Nutrition Edu.	Health Trainer	No	Group	Face to Face	Community	12	6	18	Every second week the first six months and monthly during the second semester	1.5-2 hours	Yes
Zhang 2016[138]	Control	Control	Yes		Other	No	Group	Face to Face		12	12	18	Biweekly	All participants attended group health education sessions, which were held biweekly in the first 6m and monthly in the last 6m of the intervention.	No
	Moderate exercise	Exercise only	No		Other	No	Other - education sessions: group-based; moderate-exercise sessions unsupervised	Face to Face; Telephone	Home	12	12	18	Biweekly and weekly	Participants were instructed to briskly walk at approximately 120 steps per minute for 30 minutes per session and 5 sessions per week.<br><br>All participants attended group health education sessions, which were held biweekly in the first 6m and monthly in the last 6m of the intervention.<br><br>plus,<br>Participants in the moderate exercise	No

Study ID Ref No.*	Groups:	Intervention type	Intervention faded in intensity	Features (meal replacements, nutrition education, financial incentives, intermittent fasting, content designed to help participants following programme end)	Provider †	Provider training received	Delivery		Intervention setting	Intervention timing (months)		Sessions			Intervention personalised, titrated or adapted
							Mode	Format		Last contact	End (step change in intensity)	N§	Frequency	Length per session with description for varying lengths. (minutes ‡)	
														program were required to wear pedometers and record their daily exercise in a log, which was reviewed weekly by study staff.	
	Vigorous-moderate exercise	Exercise only	No		Physician; Other	No	Individual and Group	Face to Face; Telephone	Community; Home	12	12	138	Biweekly and weekly	Participants were required to participate in 5, 30-min., vigorous exercise sessions each week supervised by a study physician at a local community health center.<br><br>All participants attended group health education sessions, which were held biweekly in the first 6m and monthly in the last 6m of the intervention.<br><br>Participants were required to participate in 5 vigorous exercise sessions each week supervised by a study physician at a local community	No

Study ID [Ref No.*]	Groups:	Intervention type	Intervention faded in intensity	Features (meal replacements, nutrition education, financial incentives, intermittent fasting, content designed to help participants following programme end)	Provider [†]	Provider training received	Delivery		Intervention setting	Intervention timing (months)		Sessions			Intervention personalised, titrated or adapted
							Mode	Format		Last contact	End (step change in intensity)	N[§]	Frequency	Length per session with description for varying lengths. (minutes [‡])	
														health center.<br>After 6 months of vigorous exercise, participants switched to moderate exercise for another 6 months.<br><br>plus,<br>Participants in the moderate exercise program were required to wear pedometers and record their daily exercise in a log, which was reviewed weekly by study staff.	

*Ref No. Reference number in main paper. [†]See table footnotes for Provider category descriptions [‡]Unless otherwise stated; [§]Exercise sessions were assumed to be unsupervised and did not contribute to the number of sessions unless otherwise stated.

Approx.: Approximately; **Appt.**: Appointment/s; **Fin. Incentives;** Financial Incentives**; GP:** General Practitioner **Inter. Fasting:** Intermittent Fasting; **Min/s:** Minute/s **M/Mths:** month/s**; MR – F =** Meal replacement (Full); **MR – P =** Meal replacement (Partial); **N:** Number; **N/A:** Not applicable; **NR:** Nor reported; **Nutrition Edu.** = Nutrition Education; **PA:** Physical Activity**; SMS**: Short Message Service; **VLCD:** Very low-calorie diet

Provider	Provider descriptions as reported in included studies
Nurse (Specialist)	
Nurse (General)	Nurse educator;<br>RNS;
GP	General internists
Physician<br>(Any doctor not a GP)	Medical doctors; Specialists in endocrinology, and internal medicine; Clinicians; Endocrinologists; Graduates in medicine; Research cardiologist; Doctoral-level clinicians (with an average of 4.8 years of experience delivering behavioral weight loss treatment); Occupational doctor.
Psychologist/ Counsellor	Therapist; Masters-level counseling psychology students; MA in behavioural psychology; Lifestyle counsellor; Graduates In psychology; Psychology graduate students; Advanced degree in behavioral psychology; Mental health counsellor; Wellness counsellors; Professional Counsellor; Psychotherapist; Psychotherapists and masters students graduate students in clinical psychology; Clinical psychology graduate students; Lifestyle counsellor; Clinical psychology graduate students; Experienced behavioural weight control counsellors; Behavior therapist; Counsellor with a degree in nutrition or physical activity
Dietitian	Dietitian; Masters of Dietetics Students
Nutritionist	Provider described by authors as nutritionist; Nutrition technician; Graduates in nutrition; Advanced degree in nutrition; Nutritional interventionist; Nutritionist (MSc in nutrition); Nutrition/Diet interventionists; Two qualified or student clinical nutritionists

Study ID Ref No.*	Groups:	Intervention type	Intervention faded in intensity	Features (meal replacements, nutrition education, financial incentives, intermittent fasting, content designed to help participants following programme end)	Provider †	Provider training received	Delivery		Intervention setting	Intervention timing (months)		Sessions			Intervention personalised, titrated or adapted
							Mode	Format		Last contact	End (step change in intensity)	N§	Frequency	Length per session with description for varying lengths. (minutes ‡)	

Physiotherapist	Physical therapist; Physical/recreational therapists
Exercise physiologist	Exercise consultants; MA in exercise physiology; Graduates in physical activity and sport science (SPAS); Advanced degree in exercise physiology; Exercise counsellors
Other Allied Health Professionals	Occupational therapist; Pharmacist; Nurses/physician assistants; Hospital staff; Social worker with special competence in CT; Medical-assistant
Health trainer	Lifestyle coaches; Mindfulness meditation instructors; Community health educator; MA in health education; Behavioural consultant; Health educator; Telephone counsellors; Trained lifestyle coaches; Health Promotion coaches; Weight loss coaches; Wellness leader; Weight Watchers leader; Trained interventionists with expertise in both content area (i.e., physical activity and nutrition) and behavioral therapy; Food advisors recruited from local community; Community Health Workers; Lifestyle activity consultant; Trained lifestyle coaches; Lifestyle Coach/ medical assistant; Masters-level staff with extensive training in behavioral weight loss; Nutrition health educator; IHM health staff graduates; 6 trained CAMWEL advisors recruited from various occupational backgrounds including healthcare, in line with the NHS health trainers initiative; Weight loss group leaders supervised by an exercise physiologist; Study coordinator (with health/nutrition background) together with a peer leader/study coordinator (experienced in adult training and self-management programs); Health educator; Degree in health sciences; Trainers (for meal replacement group); Health coach and health practitioner backgrounds and trained by the senior psychologists; Diabetes educators; EuroFIT coaches; Program providers who were trained in nutrition, education, and behavioral interventions; Masters degree–level health educators delivered health education sessions; Behaviorist; Trained lay health educators (LHEs) (community volunteers or existing senior center staff); Peer health coach; Educators held an undergraduate degree in a relevant discipline (dietician, sports scientist)
Personal Trainer	Certified exercise trainer; Trained fitness instructor; Physical activity specialist; Football coaching staff; Physical Activity Counselor; Trained interventionist and exercise coaches who were skilled in exercise science; Exercise programme supervised by a professional trainer; Fitness professional; Exercise interventionists; Exercised in a supervised setting; Trained certified technicians assessed each participant; Sports therapist; Exercise specialists;
Health care professional (not specified)	Church health advisors (CHAs) were members of their respective church's health ministry (e.g., nurses, pharmacists, physicians) and were trained by a co-investigator certified to perform GLB training; Standard clinical care provider; Hospital based care; Primary care providers; Master's trained health professionals; health professional
Other	Research staff; Behavioral specialist; PhD-level interventionists; Doctoral level graduate students; Research assistant; Case manager; Coaches; YMCA staff; Peer leader; Teacher; Interventionist; Successful group members selected through interview; Varied, may be successful slimmers; Well-trained investigators; Research assistant; PhD holders or PhD candidates in at least their third year of study; BE WELL intervention staff; Physical activity, psychological support male researcher; The tutors; Study investigator; Ergonomist; Study coordinator; Interventionist; Cooperative Extension Service Family and Consumer Sciences Agents or individuals with bachelors or masters degrees in nutrition, exercise science, or psychology; Study partner; Trained interventionists; Group facilitator; External people representing diverse areas of expertise; Two experienced coleaders; Administrative study staff (not intervention staff); Trained graduate or undergraduate students; Had backgrounds in dietetics, psychology and/or exercise physiology; Primary investigator; Study staff
If it was an OR between providers, both were listed	

Table S7. Sensitivity analyses

Outcome	Analysis
Cholesterol (standardized mean)	80 arms from 60 studies (n = 13,994 participants) were classed as not being at high risk of bias. Removing studies at high risk of bias slightly increased both the estimate of average trend in standardised cholesterol over time for the random effects and the meta-regression model. For the random intercept model, mean change increased from 0.002 to 0.008 (95% CI -0.01 to 0.027) per month, and for the meta-regression model, average change increased from 0.0006 to 0.001 (95% CI -0.02 to 0.022) per month. The association between weight difference at last follow-up and difference in standardised cholesterol was strengthened with every Kg increase in weight difference leading to an average change (95% CI) in cholesterol of 0.11 (0.05 to 0.17). Removing studies at high risk of bias from the time-to-event model did not alter the estimate of the median time (12 months).
Glycaemic control (standardized mean)	97 arms from 71 studies (n = 15,997 participants) were classed as not being at high risk of bias. Removing studies at high risk of bias slightly increased both the estimate of average trend over time for the random effects and the meta-regression model. For the random intercept model, mean trend decreased from 0.004 to -0.011 (95% CI -0.022 to 0.001) per month, and for the meta-regression model, average change decreased from 0.0007 to -0.005 (95% CI -0.02 to 0.01) per month. After excluding studies at high risk of bias, the association between weight difference at last follow-up and difference in standardised glycaemic control was significant with every Kg increase in weight difference leading to an average change (95% CI) in glycaemic control of 0.07 (0.05 to 0.13). Removing studies at high risk of bias from the time-to-event model did not alter the estimate of the median time (18 months).
Systolic blood pressure	86 arms from 64 studies (n = 18,257 participants) were classed as not being at high risk of bias. Removing studies at high risk of bias increased both the estimate of average trend in standardised cholesterol over time for the random effects and the meta-regression model. For the random intercept model, mean trend increased from 0.024 to 0.045 (95% CI 0.0008 to 0.09) per month, and for the meta-regression model, average change increased from 0.03 to 0.037 (95% CI -0.02 to 0.01) per month. After excluding studies at high risk of bias, the association between weight difference at last follow-up and difference in blood pressure remained significant with every Kg increase in weight difference leading to an average change (95% CI) in SBP of 0.64 (0.50 to 0.79). Removing studies at high risk of bias from the time-to-event model did not alter the estimate of the median time (42 months).
